# Supplementary material for: Directing GABAergic neuronal identity from human iPSCs through developmental patterning
Source: Stem Cell Reports. 2026 Sep 24;21(10):103086. doi: 10.1016/j.stemcr.2026.103086 (PMC13681393; doi:10.1016/j.stemcr.2026.103086)
Supplement: Supplementary file 1 [file mmc1.pdf]

Stem Cell Reports, Volume *21*

Supplemental Information

Directing GABAergic neuronal identity from human iPSCs through developmental patterning

Ruiqi Hu, Linda L. Boshans, Yiran Tao, Bohan Zhu, Junlong Li, Xindi Li, Rui Dang, Marianna Liang, Peiwen Cai, Mark Youssef, Gizem Inak, Yingnan Song, Xuran Wang, Alexander M. Tsankov, Joseph D. Buxbaum, Peng Jiang, Zhiping P. Pang, Sai Ma, and Nan Yang

Figure S1. Generation of immature cortical GABAergic neurons from human PSCs using a directed differentiation protocol.
Related to Figure 1.

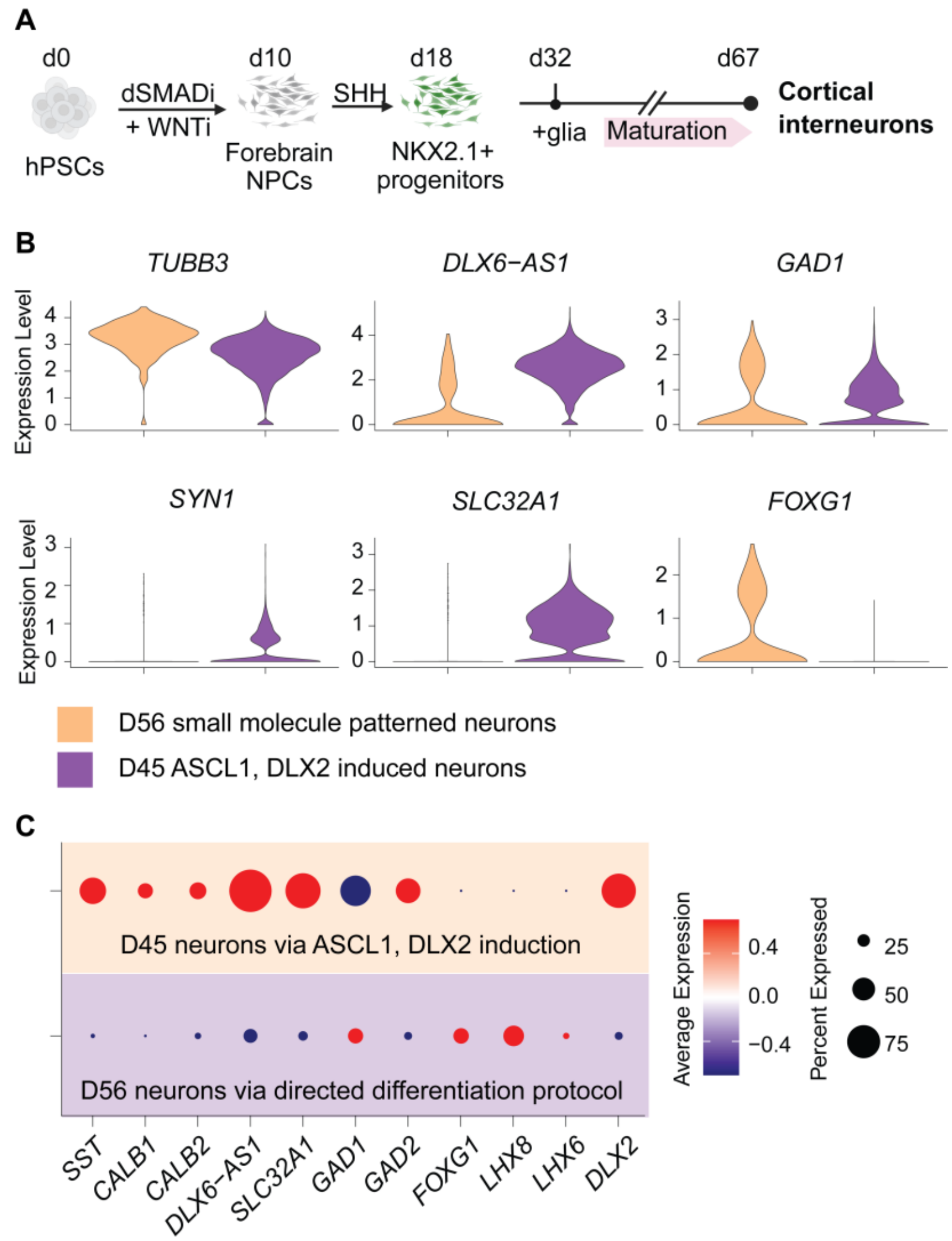

Figure S1. (**A**) Schematic overview and timeline of the small molecule method, followed by single-cell transcriptomics analysis. (**B-C**) Violin plots (**B**) and dot plots (**C**) show expression levels of neuronal identity and maturation-related marker genes in GABAergic neurons generated using either transcription factors (TFs), i.e., ASCL1 and DLX2, or small molecules.

Figure S2. Induction of neuronal fate using ASCL1/DLX2 and patterning factors.
Related to Figure 1.

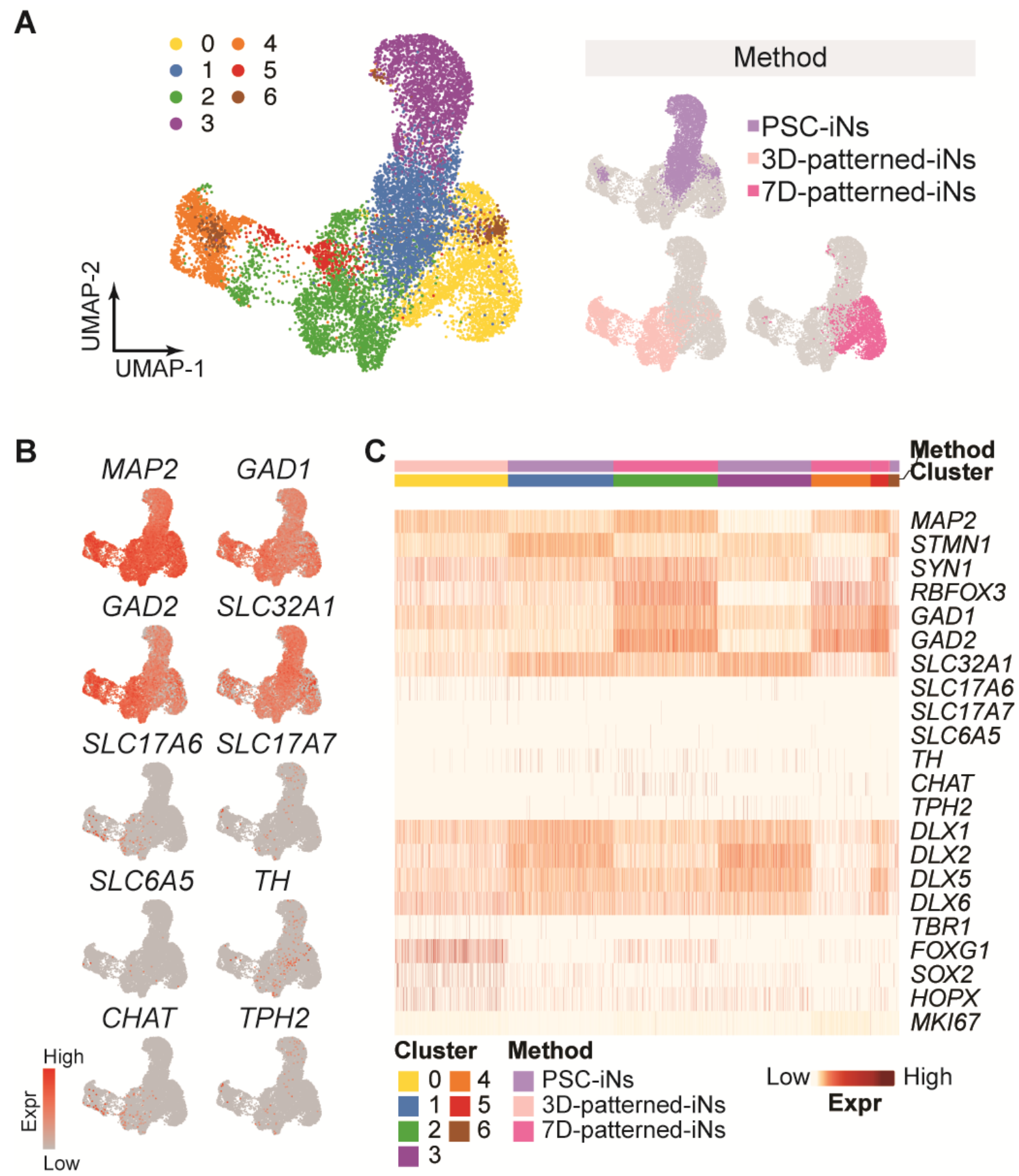

Figure S2. (**A**) UMAP embedding of 12,876 cells, color-coded by cluster identity (left panel) and differentiation method (right panel). (**B**) Feature plots for neuron subtype-specific markers. (**C**) Heatmap of cell type-specific marker gene expression across clusters. See also Table S1.

Figure S3. Expression of neuronal subtype, maturation, and brain regionality marker genes in GABAergic-induced neurons.

Related to Figure 1.

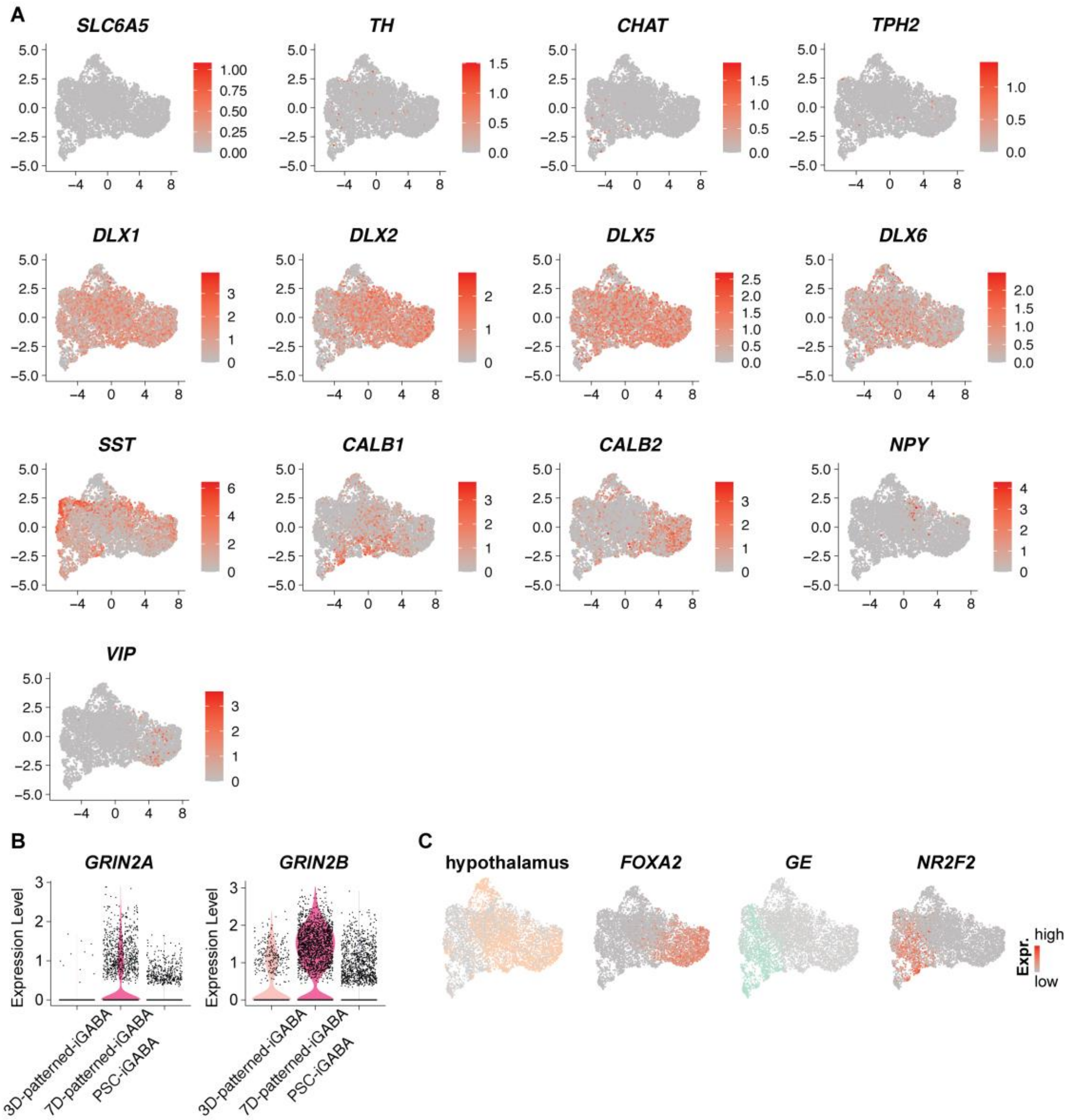

Figure S3. (A) Feature plots showing the expression of additional neuronal subtype marker genes in GABAergic-induced neurons. **(B)** Violin plots displaying the expression levels of NMDA receptor subunits in GABAergic neurons generated using different methods. **(C)** UMAP visualization depicting the respective brain regions and relevant TFs. See also Figure 1.

Figure S4. Comparison of interneuron subtype composition across differentiation methods.
Related to Figure 1.

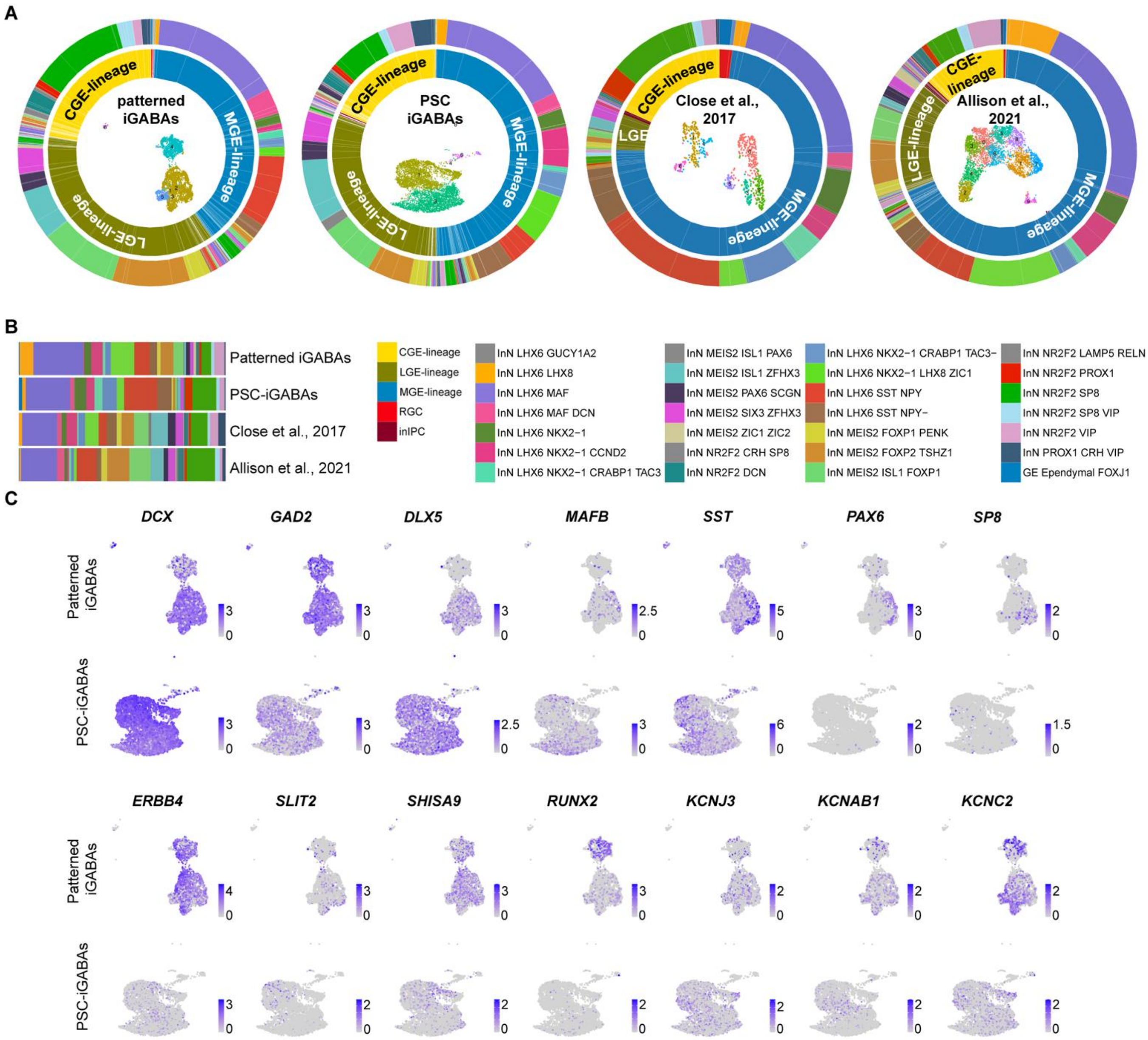

Figure S4. (**A**) Transcriptomic mapping of human PSC-derived interneurons generated using four protocols, patterned-iGABAs and PSC-iGABAs from this study, and patterning-factor–mediated differentiation datasets from Close et al. (2017) and Allison et al. (2021), to reference human brain interneuron datasets. The outer rings indicate the relative abundance of mapped interneuron subtypes. (**B**) Proportional representation of major interneuron lineage groups (MGE, CGE, LGE) across the different methods, highlighting their differing patterning tendencies. (**C**) Feature plots showing expression of representative GABAergic and interneuron-subtype marker genes (top: *DCX, GAD2, DLX5, MAFB, SST, PAX6, SP8*) and fast-spiking/PV-lineage–associated genes (bottom: *ERBB4, SLIT2, SHISA9,*

RUNX2, and the voltage-gated potassium-channel subunits *KCNJ3, KCNAB1, KCNC2*) in patterned-iGABAs and PSC-iGABAs. See also Figure 1.

Figure S5. Quantitative similarity and cross-line reproducibility of GABAergic subtype composition.
Related to Figures 1 and 2.

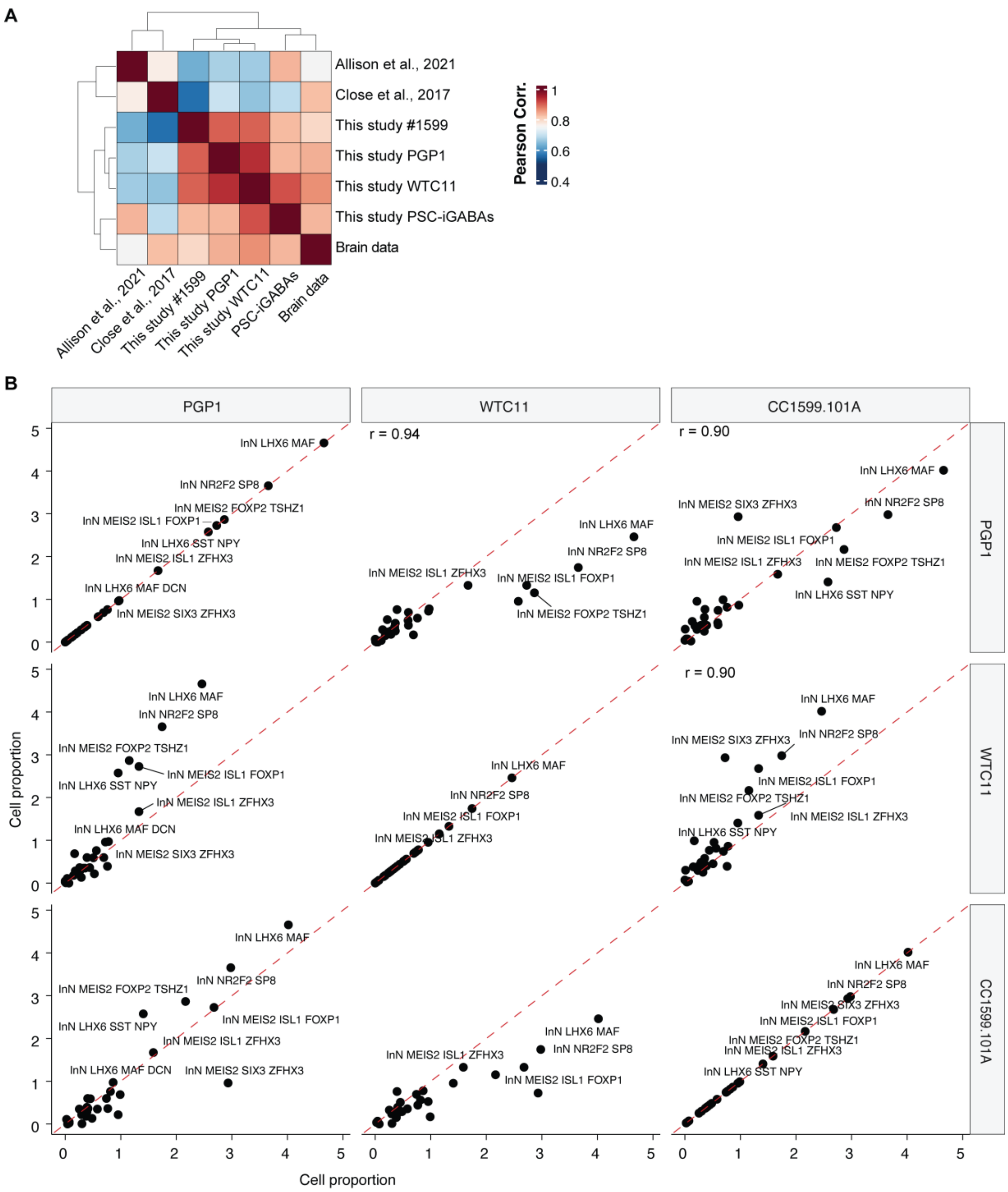

Figure S5. (**A**) Hierarchically clustered heatmap of pairwise Pearson correlations between interneuron subtype proportions, computed across GABAergic neurons generated in this study (patterned-iGABAs and PSC-iGABAs), two published protocols (Close et al., 2017; Allison et al., 2021), three iPSC lines

(PGP1, WTC11, #1599 (CC1599.101A)), and the human brain interneuron reference dataset (Feng et al., 2025). Warmer colors indicate higher correlation (greater similarity); patterned-iGABAs show the closest correspondence to the in vivo brain reference among the in vitro conditions. (**B**) Pairwise comparison of interneuron subtype proportions ("cell proportion") among the three genetically distinct iPSC lines (PGP1, WTC11, CC1599.101A). Each point represents one GABAergic subtype; the dashed line denotes identity (y = x). Pearson correlation coefficients are shown for each line pair (r = 0.90-0.94), indicating highly reproducible subtype composition across genetic backgrounds.

Figure S6. Functional comparison of patterned-iGABA and TF-only PSC-iGABA neurons.
Related to Figure 2.

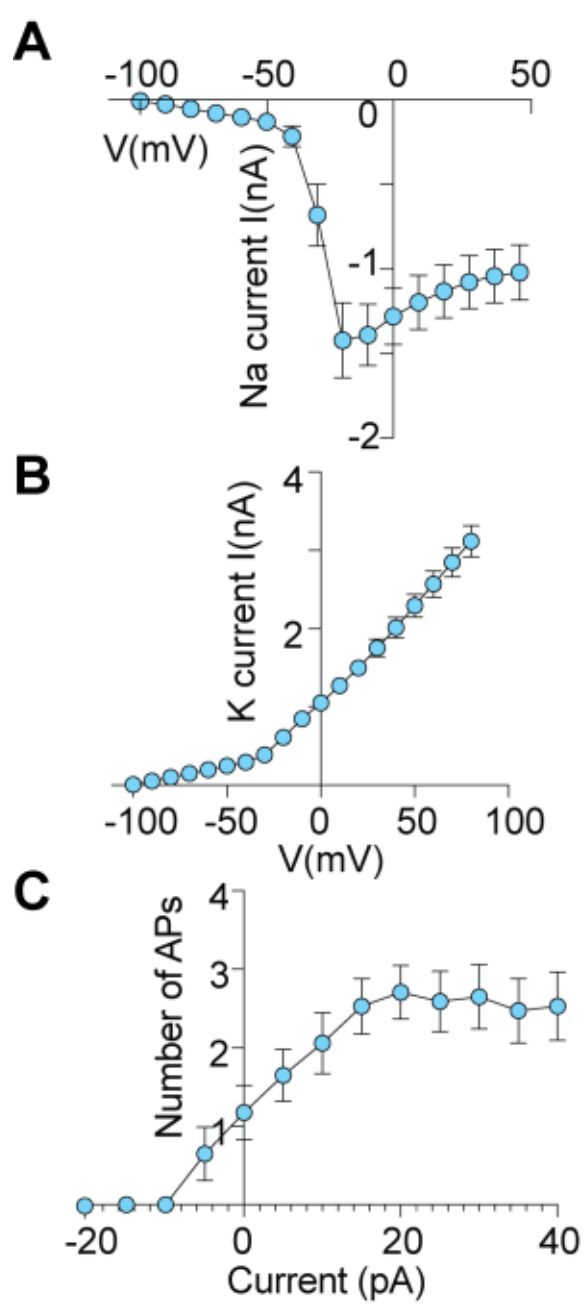

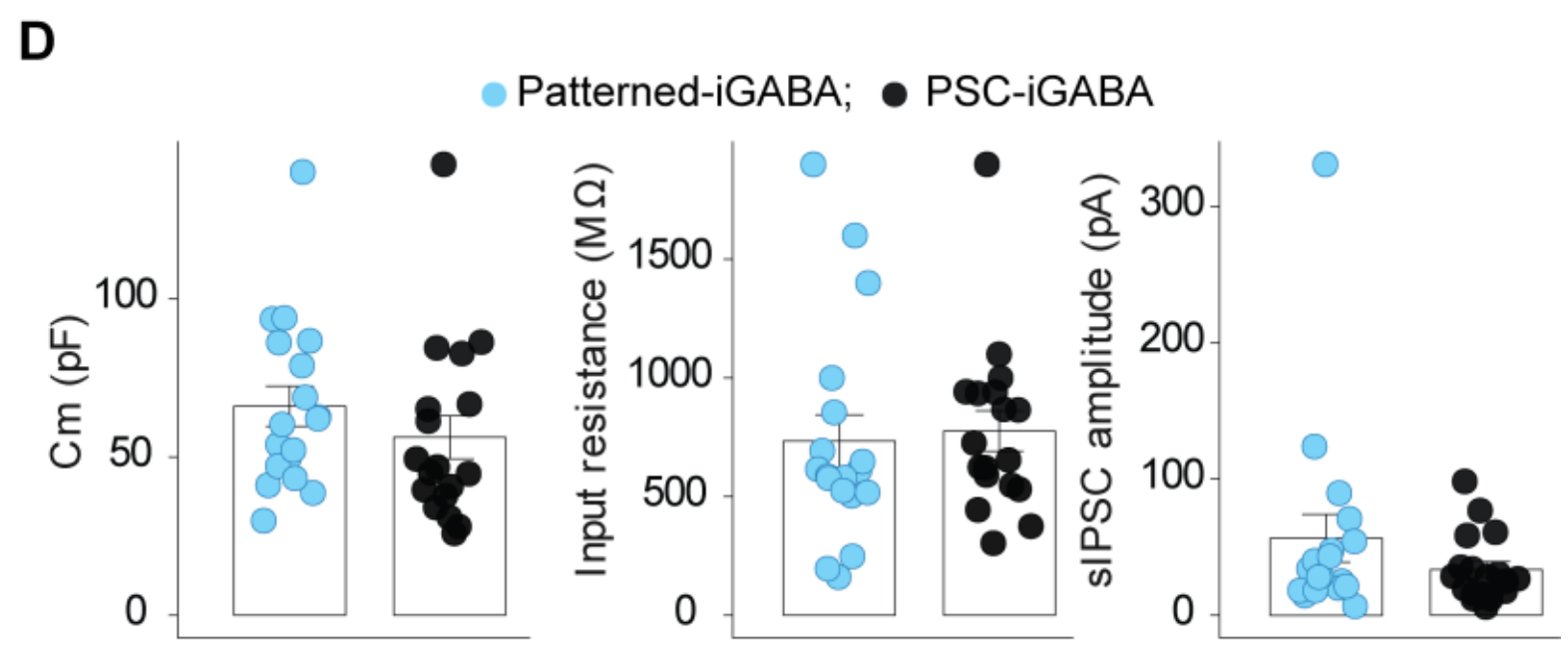

E

Parameter	Patterned iGABA (n=18)	TF-only (n=18) PSC-iGABA	Welch p
Cm (pF)	66.1 ± 6.3	56.3 ± 6.8	n.s.
Rm (MΩ)	734.6 ± 110.6	776.2 ± 85.1	n.s.
sIPSC amplitude (pA)	-56.4 ± 17.6	−33.5 ± 5.8	n.s.

Figure S6. Whole-cell patch-clamp recordings were performed on patterned-iGABA neurons after ~5 weeks of maturation on astrocytes (day 35). (**A**) Voltage-clamp current-voltage (I-V) relationship of voltage-gated sodium currents; peak inward current is plotted against step command potential (n = 20 cells). Neurons exhibited fast, voltage-dependent inward currents characteristic of functional voltage-gated Na^{+} channels. (**B**) Voltage-clamp I-V relationship of voltage-gated potassium currents; outward current is plotted against command potential (n = 20 cells). (**C**) Current-clamp input–output relationship: number of action potentials evoked by depolarizing current injections of increasing amplitude (n = 20 cells). Patterned-iGABA neurons fired repetitive, current-graded action potentials, confirming intrinsic excitability. (**D**) Comparison of intrinsic and synaptic properties between patterned-iGABA neurons (blue) and TF-only PSC-iGABA neurons (black); the latter generated with ASCL1/DLX2 alone and recorded by the same experimenter under matched conditions. Left, membrane capacitance (Cm); middle, input resistance (Rm); right, spontaneous IPSC (sIPSC) amplitude (shown as magnitude). Bars, mean ± SEM; circles, individual neurons. (**E**) Summary of the parameters in (**D**): mean ± SEM, number of cells, and P values (Welch's unpaired two-tailed t-test). No significant differences were detected between the two protocols for any measured parameter (n.s., P > 0.05), indicating similar basic membrane and inhibitory synaptic properties under the conditions tested.

Figure S7. In vivo survival and neuronal differentiation of transplanted patterned-iNs.
Related to Figure 3.

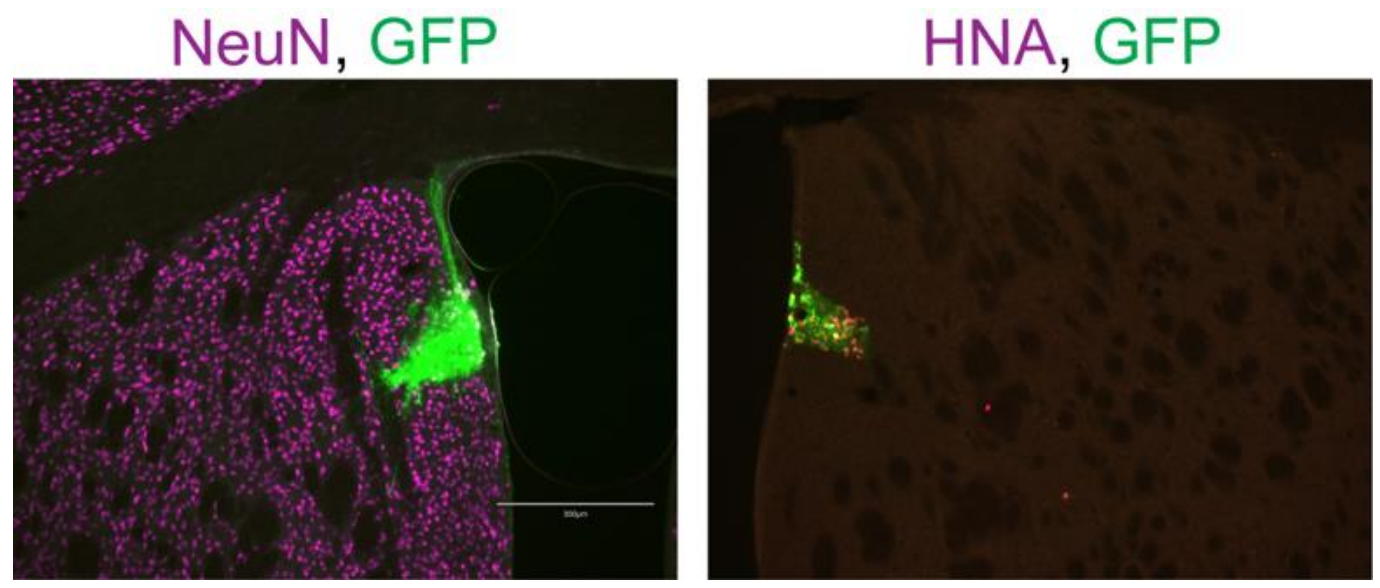

Figure S7. At 5 months post-transplantation, immunohistochemical analysis revealed that the majority of transplanted human cells, identified by human nuclear antigen (HNA) and GFP expression, differentiated into NeuN^{+} neurons. Most engrafted cells were detected in subcortical areas adjacent to the injection site, including the lateral septum and striatum. Scale bar: 300μm. These findings support long-term survival and neuronal differentiation of patterned-iNs after neonatal xenotransplantation. See also Figure 3.

Figure S8. Disease gene enrichment in GABAergic PSC-iGABAs and patterned-iGABAs.
Related to Figure 1.

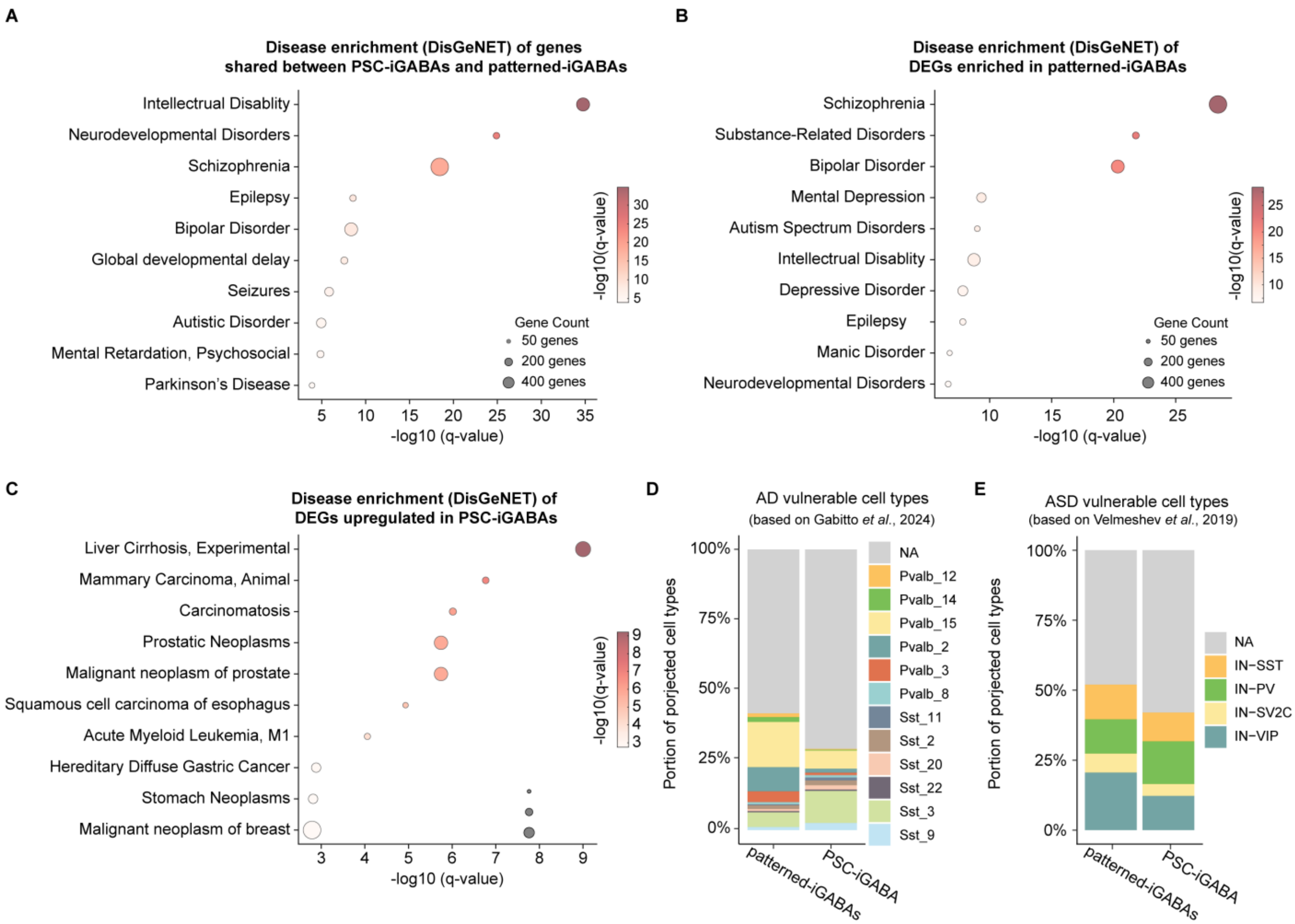

Figure S8. (A–C) Disease enrichment analysis of differentially expressed genes (DEGs) using the DisGeNET platform (Pinero et al., 2020). **(A)** Diseases associated with DEGs shared between PSC-induced GABAergic neurons (PSC-iGABAs) and patterned induced GABAergic neurons (patterned-iGABAs). **(B)** Diseases enriched in DEGs specific to patterned-iGABAs. **(C)** Diseases enriched in DEGs specific to PSC-iGABAs. The x-axis represents the gene count for each disease, and dot size indicates the number of genes associated, while color intensity reflects –log10(p-value) enrichment significance. **(D, E)** Assessment of disease-vulnerable neuronal subtypes represented in the two differentiation methods. **(D)** Cell-type overlap analysis of GABAergic neuron subtypes generated by patterned-iNs and PSC-iNs with AD-vulnerable cell types (based on Gabitto et al., 2024). **(E)** Similar analysis comparing subtype overlap ASD-vulnerable interneuron types (based on Velmeshev et al., 2019). These results demonstrate that both differentiation strategies can generate interneuron subtypes implicated in neurodevelopmental and neurodegenerative disorders, providing a foundation for disease modeling applications.

Figure S9. Gene regulatory network (GRN) analysis centered on method-specific modulators.
Related to Figure 4.

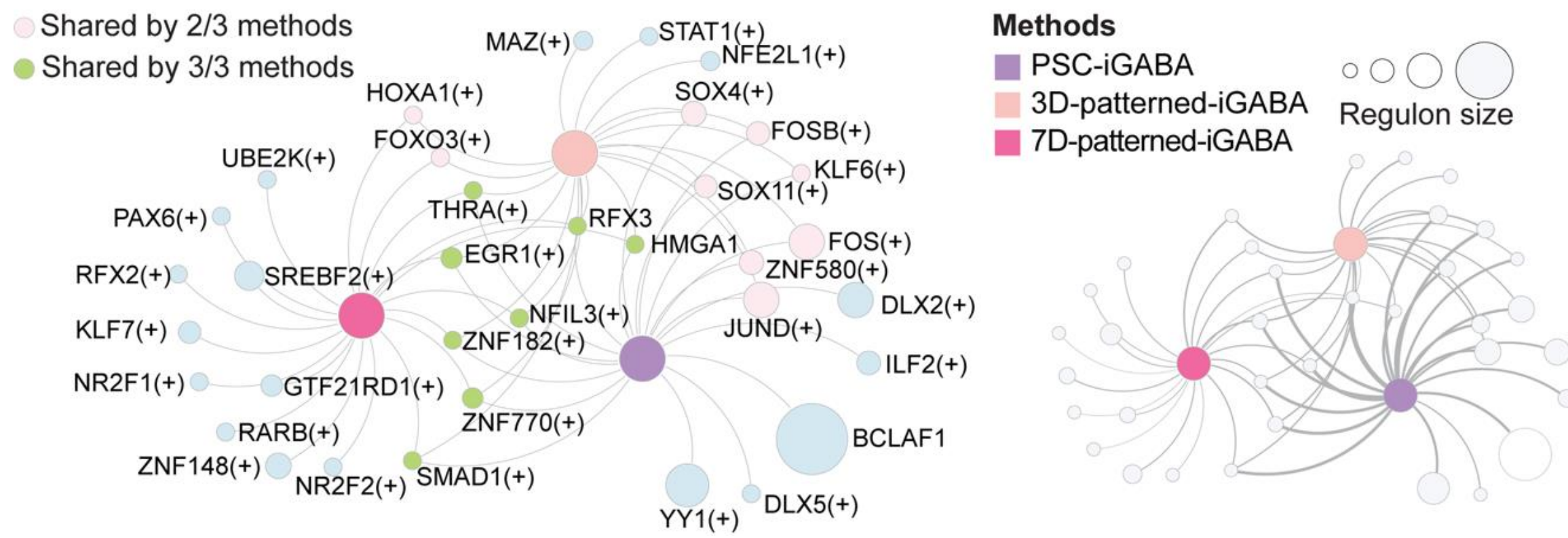

Figure S9. Left: Regulons are color-coded based on uniqueness, with node size proportional to regulon size, indicating the number of target genes regulated by each TF. Right: The same GRN with edge thickness represents the strength of the association between method modulators and regulons. See also Figure 4.

Figure S10. Differential expression of neural progenitor cell genes and their induction along the differentiation trajectory of PSC-iNs and patterned-iNs.
Related to Figure 5.

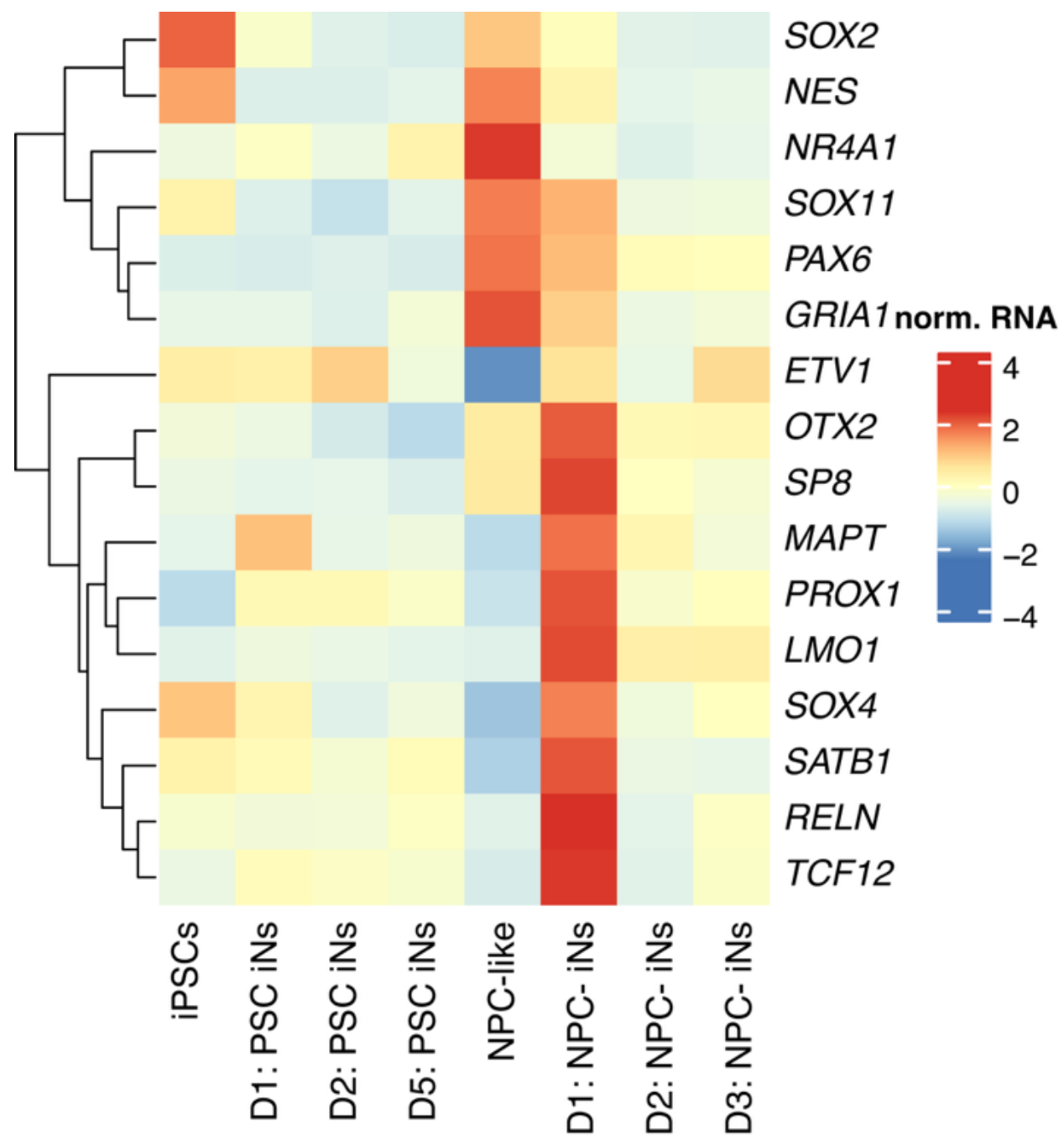

Figure S10. Heatmap showing gene expression across pseudobulk samples, aggregated from individual differentiation stages along the PSC-iNs and patterned iNs trajectory. See also Figure 5.

Figure S11. Non-interneuron lineage genes are minimally induced in GABAergic PSC-iNs and patterned-iNs.
Related to Figure 5.

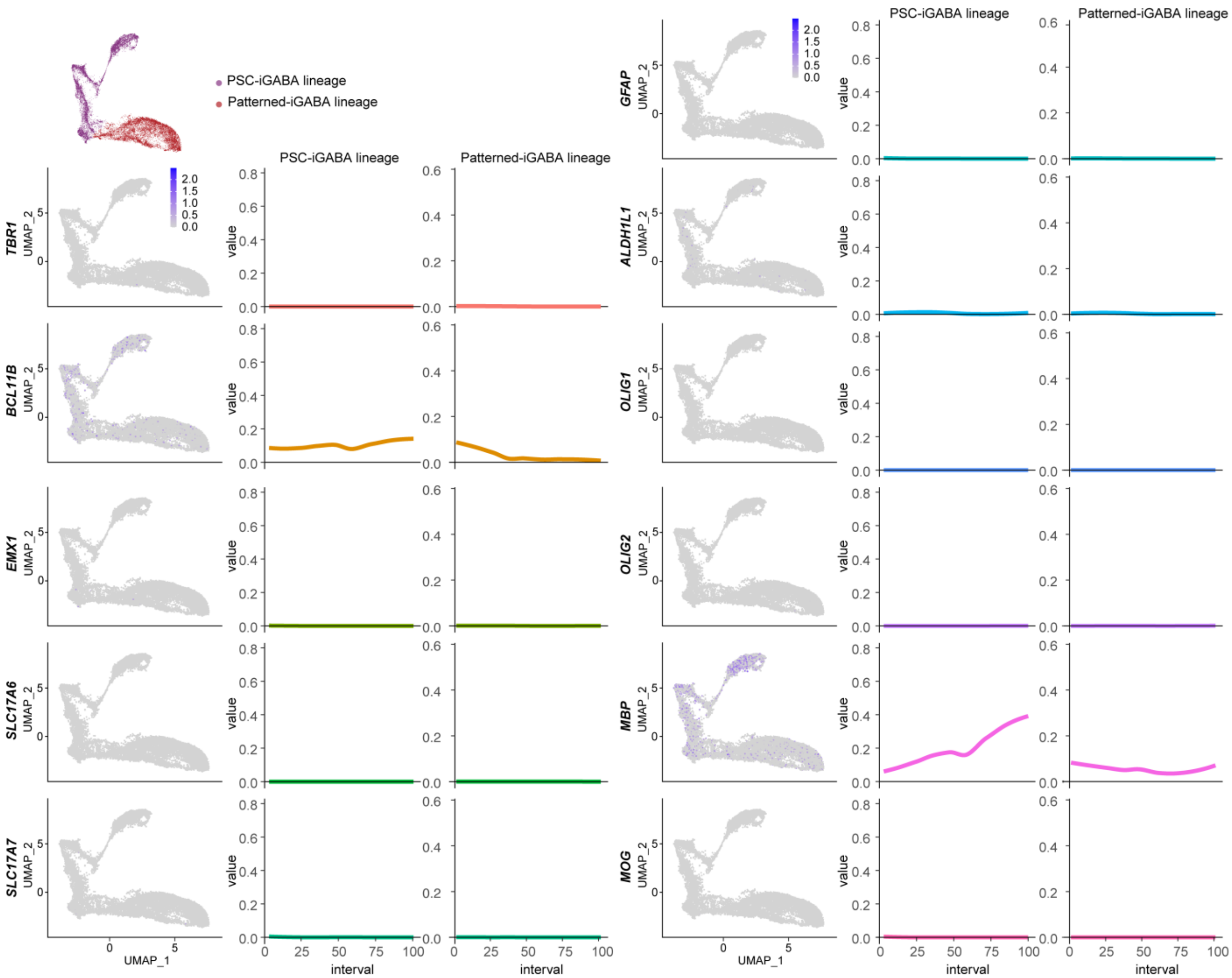

Figure S11. UMAP and pseudotime trajectory plots showing expression dynamics of non-GABAergic lineage markers in PSC-derived GABAergic neurons (PSC-iGABAs) and patterned induced GABAergic neurons (patterned-iGABAs). Expression of genes associated with alternative lineages, including *GFAP*, *ALDH1L1* (astrocyte), *TBR1*, *BCL11B*, *EMX1*, *SLC17A6*, *SLC17A7* (excitatory neurons), and *OLIG1*, *OLIG2*, *MBP*, *MOG* (oligodendrocyte), was analyzed across the differentiation trajectory. Expression of these non-interneuron markers remained low or undetectable across both lineages, indicating minimal contamination by alternative cell fates. Notably, MBP showed mild induction in the PSC-iGABA lineage but not in patterned-iGABAs, suggesting a slightly greater propensity for glial gene activation in PSC-derived cells. These data support the lineage specificity of both differentiation protocols, with rare off-target induction of non-interneuron identities. See also Figure 5.

Figure S12. Generation of iPSC lines with HA-tagged wild-type ADNP or ADNP$^{p.Tyr719*}$

Related to Figure 6.

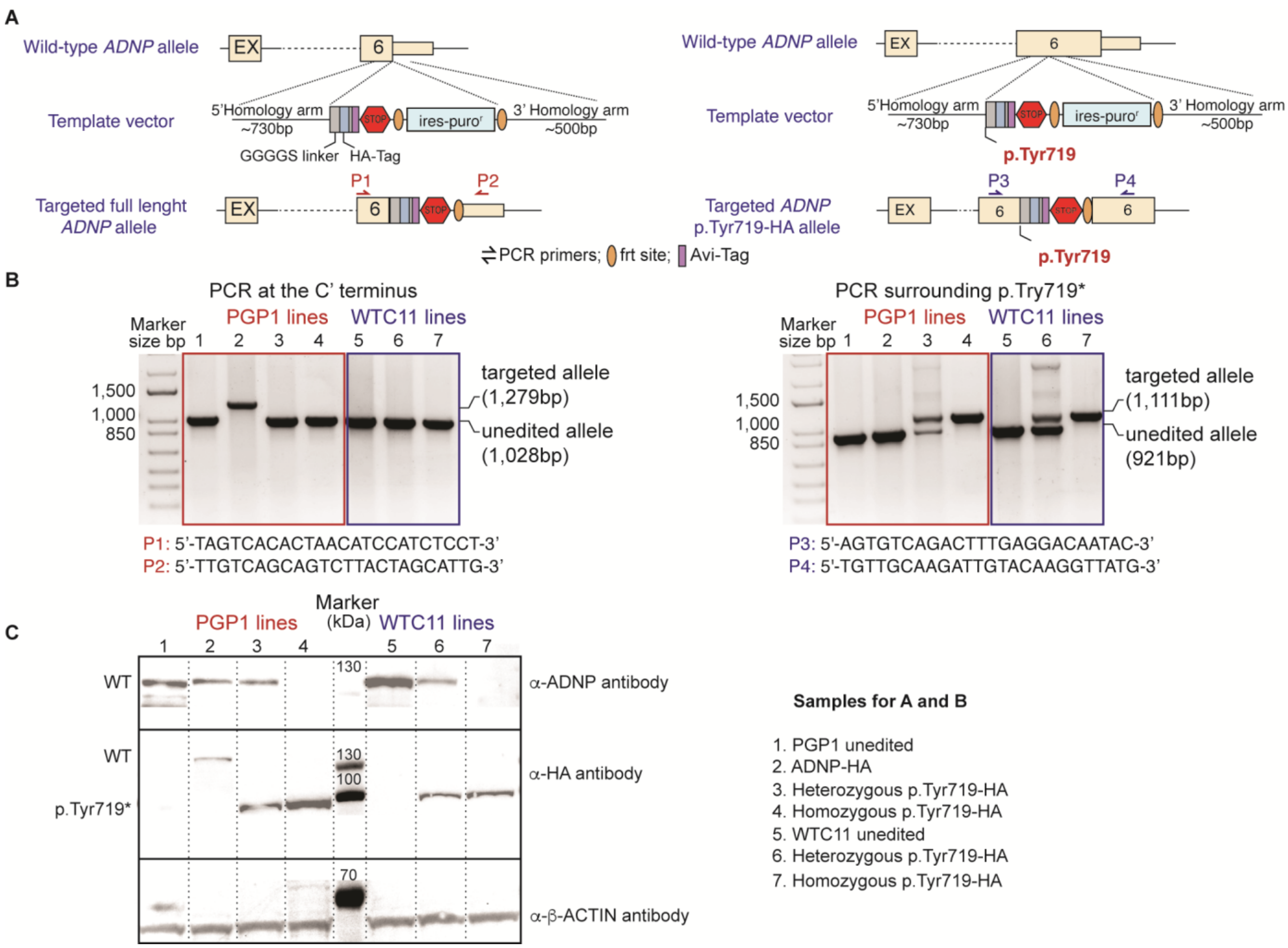

Figure S12. (**A**) Schematic representation of the generation of HA-tagged wild-type ADNP (left) and ADNP$^{p.Tyr719*}$ (right) iPSC lines. The targeting vectors included two homology arms flanking a selection cassette containing an internal ribosomal entry site (IRES), a puromycin resistance gene (puro), and a polyadenylation signal. The selection cassette was flanked by two flippase recognition target (FRT) sites, and FlipE recombinase was expressed to remove the selection cassette. (**B**) Validation of homologous recombination by PCR. The HA-tagged wild-type allele (left) produced a band of approximately 1.2 kb, while the wild-type allele produced a band of approximately 1 kb. The HA-tagged ADNPp.Tyr719* allele produced a band of approximately 1.1 kb, and the unedited allele produced a band of approximately 900 bp. Primer sequences are provided. (**C**) Immunoblotting analysis confirmed the expression of full-length ADNP and ADNP$^{p.Tyr719*}$. β-ACTIN was used as the loading control. See also Figure 6.

Figure S13. ***ADNP*** $^{p.Tyr719*}$ **mutation disrupts neurogenesis.**
Related to Figure 6.

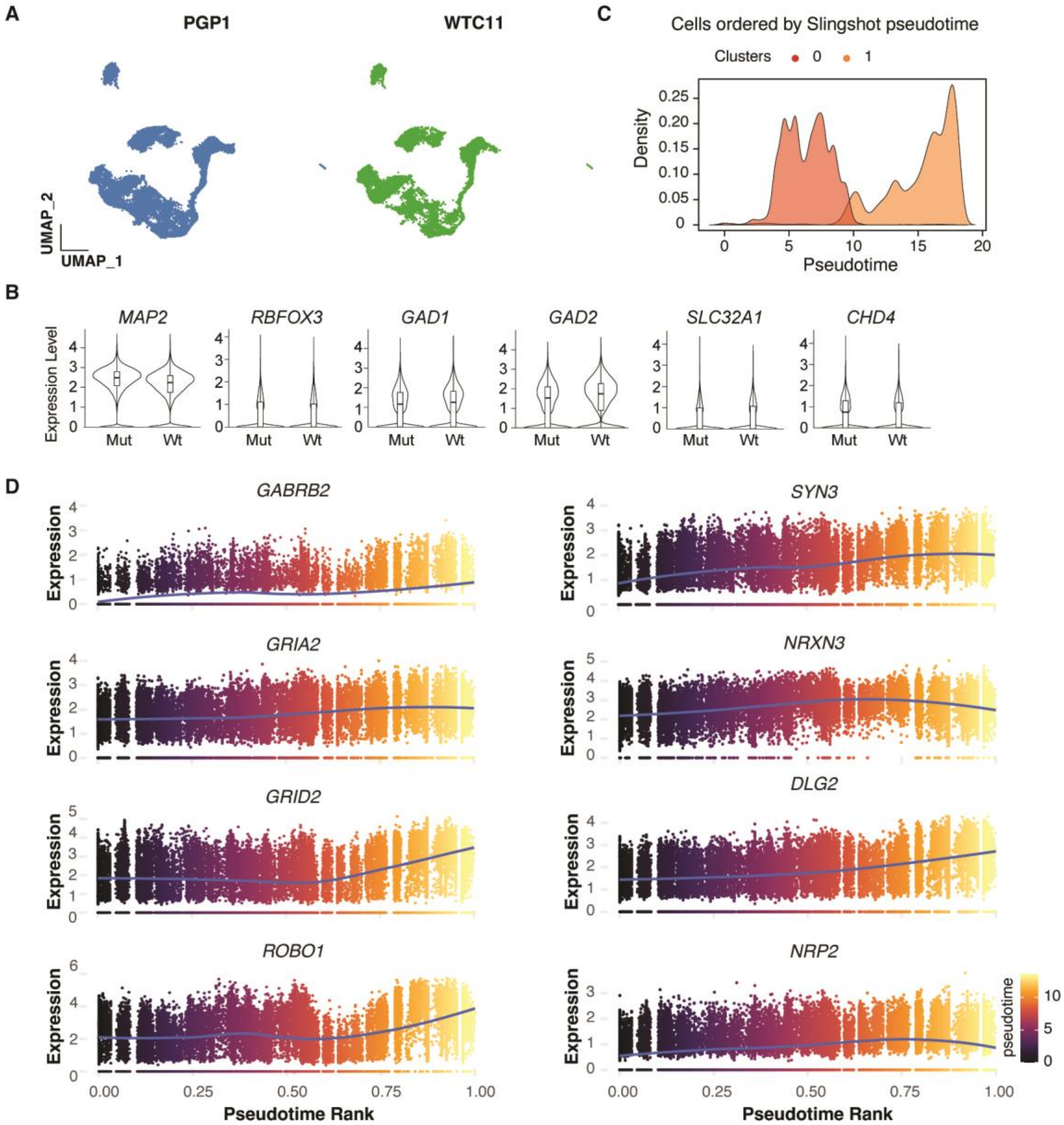

Figure S13. (**A**) Combined UMAP visualization of cells derived from different donor iPSCs. (**B**) Violin plots displaying the expression levels of pan-neuron and GABAergic neuron marker genes. (**C**) Inferred pseudotime analysis showing the progression from cluster 0 to cluster 1. The panel shows the density of each cluster across pseudotime. (**D**) Pseudotime expression plots show an increasing trend in genes related to neurite growth and synapse formation as cells progress along the pseudotime trajectory. See also Figure 6.

Figure S14. WGCNA reveals disruption of gene expression networks involved in neuronal development and function in mutant cells.
Related to Figure 6.

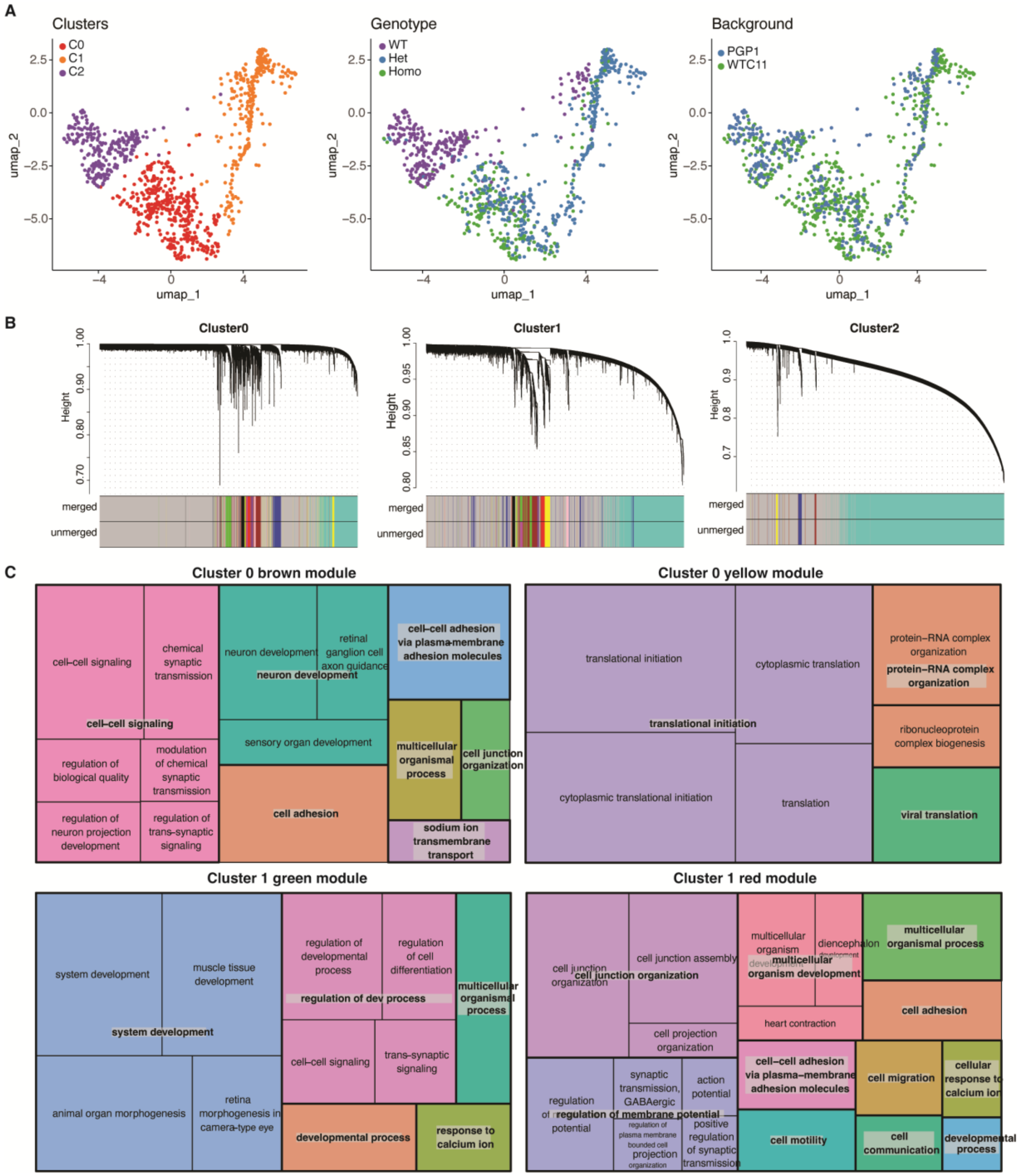

Figure S14. (A) Metacells maintain the cluster structure and accurately represent the genotypes and iPSC donor identities. **(B)** Gene expression modules identified within clusters 0, 1, and 2. **(C)** Treeplots displaying GO terms for biological processes associated with different modules in clusters consisting of mutant cells. See also Figure 6.

REFERENCES

Allison, T., Langerman, J., Sabri, S., Otero-Garcia, M., Lund, A., Huang, J., Wei, X., Samarasinghe, R.A., Polioudakis, D., Mody, I., et al. (2021). Defining the nature of human pluripotent stem cell-derived interneurons via single-cell analysis. Stem Cell Rep 16, 2548-2564. https://doi.org/10.1016/j.stemcr.2021.08.006.

Close, J.L., Yao, Z., Levi, B.P., Miller, J.A., Bakken, T.E., Menon, V., Ting, J.T., Wall, A., Krostag, A.R., Thomsen, E.R., et al. (2017). Single-Cell Profiling of an In Vitro Model of Human Interneuron Development Reveals Temporal Dynamics of Cell Type Production and Maturation. Neuron 93, 1035-1048.e5. https://doi.org/10.1016/j.neuron.2017.02.014.

Feng, X., Gao, Y., Chu, F., Shan, Y., Liu, M., Wang, Y., Zhu, Y., Lu, Q., and Li, M. (2025). Cortical arealization of interneurons defines shared and distinct molecular programs in developing human and macaque brains. Nat Commun 16, 672. https://doi.org/10.1038/s41467-025-56058-8.

Gabitto, M.I., Travaglini, K.J., Rachleff, V.M., Kaplan, E.S., Long, B., Ariza, J., Ding, Y., Mahoney, J.T., Dee, N., Goldy, J., et al. (2024). Integrated multimodal cell atlas of Alzheimer's disease. Nat Neurosci 27, 2366-2383. https://doi.org/10.1038/s41593-024-01774-5.

Pinero, J., Ramirez-Anguita, J.M., Sauch-Pitarch, J., Ronzano, F., Centeno, E., Sanz, F., and Furlong, L.I. (2020). The DisGeNET knowledge platform for disease genomics: 2019 update. Nucleic Acids Res 48, D845-D855. https://doi.org/10.1093/nar/gkz1021.

Velmeshev, D., Schirmer, L., Jung, D., Haeussler, M., Perez, Y., Mayer, S., Bhaduri, A., Goyal, N., Rowitch, D.H., and Kriegstein, A.R. (2019). Single-cell genomics identifies cell type-specific molecular changes in autism. Science 364, 685-689. https://doi.org/10.1126/science.aav8130.
