# Supplementary material for: Directing GABAergic neuronal identity from human iPSCs through developmental patterning
Source: Stem Cell Reports. 2026 Sep 24;21(10):103086. doi: 10.1016/j.stemcr.2026.103086 (PMC13681393; doi:10.1016/j.stemcr.2026.103086)
Supplement: Supplementary file 2 [file mmc12.pdf]

STEM CELL REPORTS ISSCR
Article
OPEN ACCESS

Directing GABAergic neuronal identity from human iPSCs through developmental patterning

Ruiqi Hu,[1,2,3,4,12] Linda L. Boshans,[1,2,3,4,12] Yiran Tao,[1,2,3,4] Bohan Zhu,[5] Junlong Li,[6,7] Xindi Li,[6,7] Rui Dang,[8] Marianna Liang,[1,2,3,4,9] Peiwen Cai,[5] Mark Youssef,[1,2,3,4] Gizem Inak,[1,2,3,4] Yingnan Song,[5,10,11] Xuran Wang,[5,10,11] Alexander M. Tsankov,[5] Joseph D. Buxbaum,[5,10,11] Peng Jiang,[8] Zhiping P. Pang,[6,7] Sai Ma,[5,*] and Nan Yang[1,2,3,4,13,*]

[1]Nash Family Department of Neuroscience, Friedman Brain Institute, Icahn School of Medicine at Mount Sinai, New York, NY 10029, USA
[2]Alper Center for Neurodevelopment and Regeneration, Icahn School of Medicine at Mount Sinai, New York, NY 10029, USA
[3]Institute for Regenerative Medicine, Icahn School of Medicine at Mount Sinai, New York, NY 10029, USA
[4]Black Family Stem Cell Institute, Icahn School of Medicine at Mount Sinai, New York, NY 10029, USA
[5]Department of Genetics and Genomic Sciences, Icahn School of Medicine at Mount Sinai, New York, NY 10029, USA
[6]Center for NeuroMetabolism, The Child Health Institute of New Jersey, Rutgers Robert Wood Johnson Medical School, New Brunswick, NJ 08901, USA
[7]Department of Neuroscience and Cell Biology, Rutgers Robert Wood Johnson Medical School, New Brunswick, NJ 08901, USA
[8]Department of Cell Biology and Neuroscience, Rutgers University, Piscataway, NJ 08854, USA
[9]Department of Neurology, Icahn School of Medicine at Mount Sinai, 1425 Madison Avenue, New York, NY 10029, USA
[10]Seaver Autism Center for Research and Treatment, Icahn School of Medicine at Mount Sinai, New York, NY 10029, USA
[11]Department of Psychiatry, Icahn School of Medicine at Mount Sinai, New York, NY 10029, USA
[12]These authors contributed equally
[13]Lead contact
*Correspondence: sai.ma@mssm.edu (S.M.), nan.yang1@mssm.edu (N.Y.)
https://doi.org/10.1016/j.stemcr.2026.103086

SUMMARY

GABAergic interneurons are implicated in numerous neurodevelopmental and neuropsychiatric disorders. Transcription factor (TF)-mediated induction of human pluripotent stem cells (PSCs) rapidly generates GABAergic neurons, yet their regional identity remains poorly defined. Here, we show that the chromatin state of the starting population constrains the neuronal identities specified by ASCL1 and DLX2. Brief neural patterning via dual-SMAD and WNT inhibition prior to TF induction biases PSCs toward GABAergic neurons with forebrain-associated identities, whereas TF induction of PSCs yields neurons with broader regional signatures, including hypothalamic and thalamic programs. Benchmarking multiple approaches against a human fetal brain atlas reveals distinct regional identity biases and disease gene enrichment profiles across methods, providing a framework for selecting differentiation strategies for disease modeling. Applying this approach to ADNP syndrome, we demonstrate that the recurrent p.Tyr719* mutation disrupts interneuron maturation and subtype-associated transcriptional programs and impairs inhibitory synaptic transmission in human GABAergic neurons.

INTRODUCTION

Inhibitory GABAergic (γ-aminobutyric acid, GABA) neurons comprise a highly diverse class of cells essential for neural circuit organization (Wamsley and Fishell, 2017). Forebrain interneurons originate from progenitor domains in the ventral telencephalon—the medial ganglionic eminence (MGE), caudal ganglionic eminence (CGE), and lateral ganglionic eminence (LGE)—which give rise to populations with diverse molecular and functional properties (Marin, 2012). MGE-derived interneurons predominantly mature into parvalbumin (PV)- or somatostatin-expressing (SST) cortical interneurons (Butt et al., 2008), whereas CGE generates vasoactive intestinal peptide (VIP)-, calretinin (CR)-, LAMP5-, and CCK-expressing populations (Miyoshi et al., 2010). LGE-derived interneurons also contribute to cortical inhibitory populations, particularly in frontal regions, and are marked by MEIS2, FOXP2, and PAX6 (Li et al., 2023; Schmitz et al., 2022).

Perturbations in interneuron specification and function have been implicated in autism spectrum disorder (ASD), epilepsy, schizophrenia, Alzheimer disease (AD), and other neurodevelopmental and psychiatric disorders (Inan et al., 2013; Katsarou et al., 2017; Marin, 2012). Importantly, interneurons arising from all three developmental domains contribute to disease-relevant phenotypes. MGE-derived PV^+ and SST^+ interneurons are linked to disrupted excitation/inhibition (E/I) balance across multiple conditions, while CGE-derived interneurons, proportionally expanded in humans and non-human primates (Hansen et al., 2013; Krienen et al., 2020; Yu et al., 2021), have been implicated across neuropsychiatric disorders (Goff and Goldberg, 2021; Skene et al., 2018) and are the primary source of tumors and lesions in tuberous sclerosis complex (TSC) (Eichmuller et al., 2022). These findings underscore the need for *in vitro* systems capable of generating interneurons representing diverse developmental origins.

Human pluripotent stem cell (PSC)-based approaches have enabled scalable generation of GABAergic neurons for mechanistic and disease-focused studies. Conventional differentiation strategies that rely on small-molecule patterning to recapitulate MGE development (Close et al.,

Published by Elsevier Inc. on behalf of International Society for Stem Cell Research.
This is an open access article under the CC BY license (http://creativecommons.org/licenses/by/4.0/).

2017; Maroof et al., 2013; Nicholas et al., 2013) can generate interneuron populations but often exhibit protracted maturation timelines, limited CGE/LGE representation, and variable efficiency across PSC lines. Direct reprogramming through forced expression of proneural transcription factors (TFs) offers a faster and more reproducible alternative (Pang et al., 2011; Zhang et al., 2013). In particular, we previously showed that co-expression of ASCL1 and DLX2 efficiently converts PSCs into GABAergic induced neurons (iNs) that express markers associated with multiple interneuron lineages and enable modeling of inhibitory synapse phenotypes (Marro et al., 2019; Sanchez-Priego et al., 2022; Yang et al., 2017). However, TF induction alone generates a limited spectrum of neuronal identities, and the cellular composition, regional correspondence, and heterogeneity of GABAergic iNs remain incompletely defined at single-cell resolution. Recent studies suggest that combining TF induction with developmental pre-patterning can improve regional specification. For example, dual-SMAD and WNT inhibition prior to NGN2 induction promotes cortical excitatory neuron identities, and a similar sequential strategy has been applied to lower motor neurons (Limone et al., 2023; Nehme et al., 2018). Whether comparable approaches can guide the specification of transcriptionally distinct GABAergic interneurons remains unexplored, and the mechanistic basis by which the chromatin state of the starting population shapes TF-mediated fate decisions remains unresolved.

To address these questions, we combined brief neural patterning via dual-SMAD and WNT inhibition with inducible expression of ASCL1 and DLX2. Using single-cell RNA sequencing (scRNA-seq) and joint chromatin accessibility profiling, we show that the chromatin state of the starting population constrains the neuronal identities that ASCL1 and DLX2 can specify: patterned progenitors give rise to GABAergic neurons with cortical and ganglionic eminence-associated molecular signatures, whereas naive PSCs yield neurons with mixed regional identities, including hypothalamic and thalamic signatures. We systematically compare these TF-based approaches with a conventional small-molecule protocol, benchmarking all methods against a human fetal brain atlas to reveal distinct regional identity biases and disease gene enrichment profiles. To demonstrate the disease-modeling utility of this framework, we apply it to *ADNP* (activity-dependent neuroprotective protein), one of the most frequently mutated genes in syndromic autism (Fu et al., 2022). Although prior studies have primarily examined ADNP function in excitatory neuron models, the consequences of *ADNP* mutations in human inhibitory neurons—a lineage central to E/I balance—remain poorly defined. Using patterned GABAergic iNs, we show that the recurrent p.Tyr719* mutation disrupts interneuron maturation and subtype-associated transcriptional programs and impairs inhibitory synaptic transmission, providing, to our knowledge, the first characterization of an ADNP mutation in human GABAergic neurons.

RESULTS

ASCL1 and DLX2 induce GABAergic neurons from patterned progenitors

Previously, we reported that forced expression of ASCL1 and DLX2 rapidly generates GABAergic neurons from human PSCs (Figure 1A). Although these PSC-derived iNs express canonical inhibitory markers, including GAD1/2, DLXs, and SLC32A1 (vGAT), their cellular heterogeneity and correspondence to *in vivo* interneuron populations remain unclear. To address this, we performed scRNA-seq on PSC-iNs 42 days after induction (Figure 1A). For comparison, we generated interneurons using a well-established directed differentiation protocol designed to specify ventral telencephalic progenitors through dual SMAD inhibition, WNT inhibition, and SHH activation (Maroof et al., 2013). After five weeks in astrocyte co-culture (Figure S1), these neurons expressed GABAergic markers such as *GAD1* and *DLX6-AS* but showed limited expression of synaptic genes, including *SLC32A1* and *SYN1*. In contrast to PSC-iNs, they expressed *FOXG1*, a defining marker of forebrain identity, suggesting that they resemble immature cortical interneurons (Figure S1). The absence of *FOXG1* in PSC-iNs indicates that they may correspond to more posterior GABAergic populations.

These observations prompted us to test whether extrinsic patterning cues could synergize with ASCL1 and DLX2 to generate inhibitory neurons associated with regionally biased identities. Human PSCs were first patterned toward neuroectoderm by inhibiting TGF-β and BMP signaling ca(SB431542, LDN193189) and by transient WNT inhibition (XAV939) (Ciceri et al., 2024). Following patterning, doxycycline (DOX) was applied to induce ASCL1 and DLX2 expression, together with the γ-secretase inhibitor DAPT, to promote neuronal differentiation. Cells were then dissociated and co-cultured with mouse astrocytes to support neuronal maturation and synapse formation. To assess whether the duration of TF exposure influences neuronal fate, DOX was withdrawn after either 3 or 7 days, generating three-dimensional (3D)-patterned-iNs and 7D-patterned-iNs, respectively. These neurons are collectively referred to as patterned-iNs, reflecting the integration of regional patterning with TF-driven specification. In parallel, PSC-iNs received continuous DOX for 14 days. Five weeks after astrocyte co-culture, $MAP2^{+}/DLX2^{+}$ GABAergic neurons were observed across all conditions (Figure 1A).

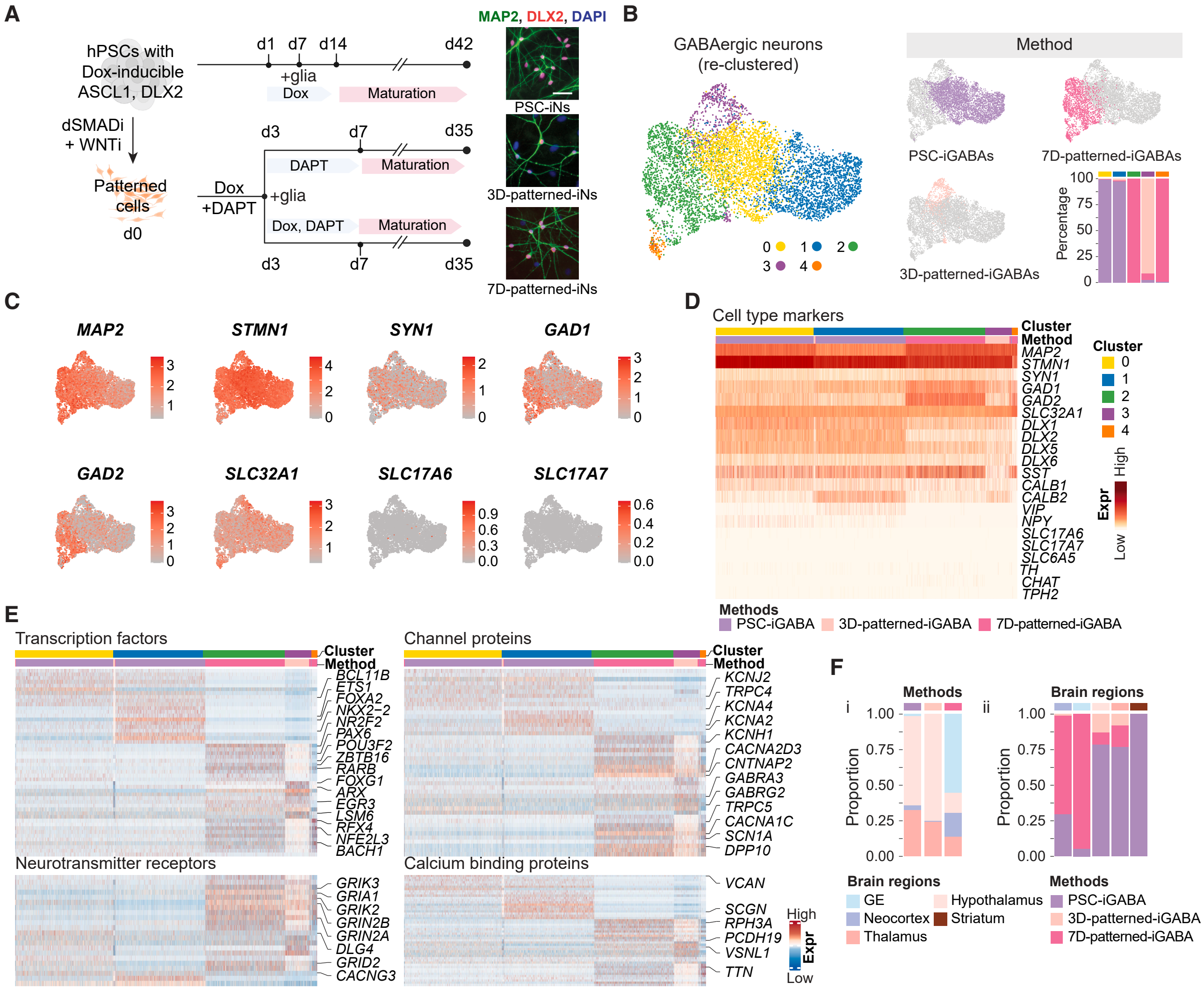

Figure 1. Generation and molecular profiling of GABAergic iNs derived using distinct differentiation strategies

(A) Schematic of PSC-iNs (TF-only) and 3D- or 7D-patterned-iNs (dual-SMAD inhibition [dSMADi] and WNT inhibition [WNTi] followed by ASCL1/DLX2 induction). Representative MAP2 and DLX2 immunostaining. Scale bars, 50 μm.

(B) UMAP of SLC32A1^{+} GABAergic iNs colored by cluster and method; right, cluster distribution and cell proportions by condition.

(C) Feature plots of inhibitory and excitatory marker genes.

(D and E) Heatmap of cluster-specific markers, TFs, ion channels, neurotransmitter receptors, and calcium-binding proteins, including genes implicated in ASD and epilepsy.

(F) Brain-region mapping using human fetal references: (i) proportion of cells from each method assigned to regional identities; (ii) contribution of each method to annotated regions. Ganglionic eminence (GE)- and cortex-like identities are enriched in 7D-patterned-iNs, whereas PSC-iNs preferentially map to hypothalamic and thalamic regions.

See also Figures S1–S8 and Tables S1 and S2.

To gain deeper insights into the cellular composition of these cultures, we performed scRNA-seq of 12,876 cells across various differentiation strategies, including 5,314 PSC-iNs, 2,899 3D-patterned-iNs, and 4,663 7D-patterned-iNs that passed quality control (Table S1). Uniform manifold approximation and projection (UMAP) analysis revealed seven transcriptionally distinct clusters segregating by differentiation method (Figure S2). Most cells expressed neuronal markers *MAP2*, *STMN1*, and *SYN1* (Figure S2), and 86.2% exhibited GABAergic identity, defined by GAD1, GAD2, SLC32A1, and DLX genes. A small fraction of cells was identified as glutamatergic, based on *SLC17A6/7* expression (3D-patterned-iNs: 42/2,899; 7D-patterned-iNs: 56/4,663; PSC-iNs: 10/5,314), while only

rare cells expressed CHAT (cholinergic, 0.02%) or TH (dopaminergic, 0.06%). Notably, *SLC32A1* expression was substantially lower in 3D-patterned-iNs than in 7D-patterned-iNs (19.4% vs. 50.9%), whereas 3D-patterned-iNs showed increased expression of progenitor-associated genes such as *SOX2* (7.0% vs. 0.94%). These findings suggest that 3 days of ASCL1/DLX2 induction after patterning are insufficient for complete neuronal and GABAergic conversion, whereas extended TF induction promotes a more advanced neuronal and GABAergic differentiation state. Together, these data indicate that TF induction duration and extrinsic patterning cues shape the differentiation state and cellular composition of induced GABAergic neuronal cultures.

Molecularly diverse GABAergic neurons generated by distinct differentiation strategies

To characterize heterogeneity among induced GABAergic neurons (iGABAs, we extracted *SLC32A1*$^+$ neurons, comprising 4,086 PSC-iGABAs, 574 3D-patterned-iGABAs, and 1,915 7D-patterned-iGABAs. Unsupervized clustering identified five major molecular clusters that segregated according to differentiation strategy (Figure 1B). All clusters expressed canonical GABAergic lineage markers and lacked markers of alternative neuronal identities (Figures 1C and S3). Clusters showed variable enrichment of inhibitory neuron subtype markers, including *SST*, *CALB1*, *CALB2*, *VIP*, and *NPY* (Figures 1C, 1D, and S3A). When examining the differentially expressed TFs across these clusters, we found *FOXG1* expressed in patterned-iGABAs (clusters 3 and 4). The gene expression signature of 7D-patterned-iGABAs (cluster 2) reflected transcriptional signatures associated with CGE- and LGE-derived interneurons, marked by *NR2F2* (*COUP-TFII*), *PAX6*, and *ISL1* (Feng et al., 2025; Schmitz et al., 2022). In contrast, PSC-iGABAs were enriched for *FOXA2* and *NKX2-2*, markers associated with hypothalamic inhibitory neurons (Kim et al., 2022). Clusters also differed in the expression of ion channels, neurotransmitter receptors, and calcium-binding proteins, highlighting the molecular diversity generated by distinct differentiation strategies (Figure 1E; Table S2). Notably, patterned neurons showed elevated expression of NMDAR subunits *GRIN2A* and *GRIN2B* (Figure S3B). *GRIN2B* is predominantly expressed during prenatal cortical development, whereas *GRIN2A* expression emerges largely postnatally (Traynelis et al., 2010). This pattern aligns with prior reports demonstrating that combining regional patterning with TF induction promotes synaptic maturation (Nehme et al., 2018).

To assign regional identities, we mapped iGABAs to human fetal brain scRNA-seq references (Bhaduri et al., 2021; Herb et al., 2023). iGABAs mapped across multiple forebrain regions, including the hypothalamus, thalamus, neocortex, and GE (Figure 1F). Although all strategies generated neurons spanning multiple regions, GE- and neocortex-associated populations were enriched in patterned conditions, whereas PSC-iGABAs preferentially mapped to hypothalamic and thalamic identities. These assignments were supported by regional marker expression, including *FOXA2* in hypothalamic clusters (Kim et al., 2022) and *NR2F2* in the GE-associated cluster (Figure S3C).

To benchmark interneuron diversity, we performed comparative single-cell transcriptomic analyses of 7D-patterned-iGABAs, PSC-iGABAs, and GABAergic neurons generated using published small-molecule-based protocols (Close et al., 2017; Allison et al., 2021). Using the human developmental brain atlas from Feng et al. (2025) for uniform annotation, PSC-derived GABAergic neurons across protocols mapped to MGE-, CGE-, and LGE-derived lineages (Figures S4A and S4B). Small-molecule patterning protocols were strongly biased toward MGE-derived interneuron lineages, with limited CGE/LGE representation. In contrast, PSC-iGABAs and patterned-iGABAs spanned multiple developmental origins, although their subtype-associated transcriptional states differed, indicating that differentiation strategy shapes molecular identity beyond broad lineage assignment. Correlation analysis showed that patterned-iGABAs most closely resembled the subtype distributions ($r = 0.83$), comparable to previously reported methods, which showed $r = 0.82$ (Close et al., 2017) and $r = 0.74$ (Allison et al., 2021) (Figure S5A). Reference mapping across three independent iPSC lines yielded highly concordant subtype distributions (Figure S5B; $r = 0.90$–0.94), indicating robust reproducibility across genetically distinct PSC lines.

Functional properties and synaptic integration of patterned GABAergic iNs

Because 3D-patterned GABAergic iNs retain immature transcriptomic features, subsequent analyses focused on 7D-patterned GABAergic iNs (hereafter referred to as GABAergic-patterned iNs or patterned-iGABAs). Functional properties were evaluated at day 35 using electrophysiology and immunofluorescence (Figure 2A). Voltage-clamp recordings revealed inward sodium currents in response to depolarizing steps, and current-clamp traces showed repetitive action potential firing in response to current injection (Figures 2B and 2C). Passive membrane properties were consistent with functionally active neurons (Figure 2D). Spontaneous inhibitory postsynaptic currents (sIPSCs) were detected in the presence of CNQX and abolished by picrotoxin, confirming GABAergic synaptic transmission (Figures 2E–2G). Immunostaining confirmed expression of markers associated with multiple interneuron lineages (Figure 2H); the proportions of MAP2$^+$ neurons expressing DLX2, DLX5, SST, calbidin (CB), and CR were reproducible

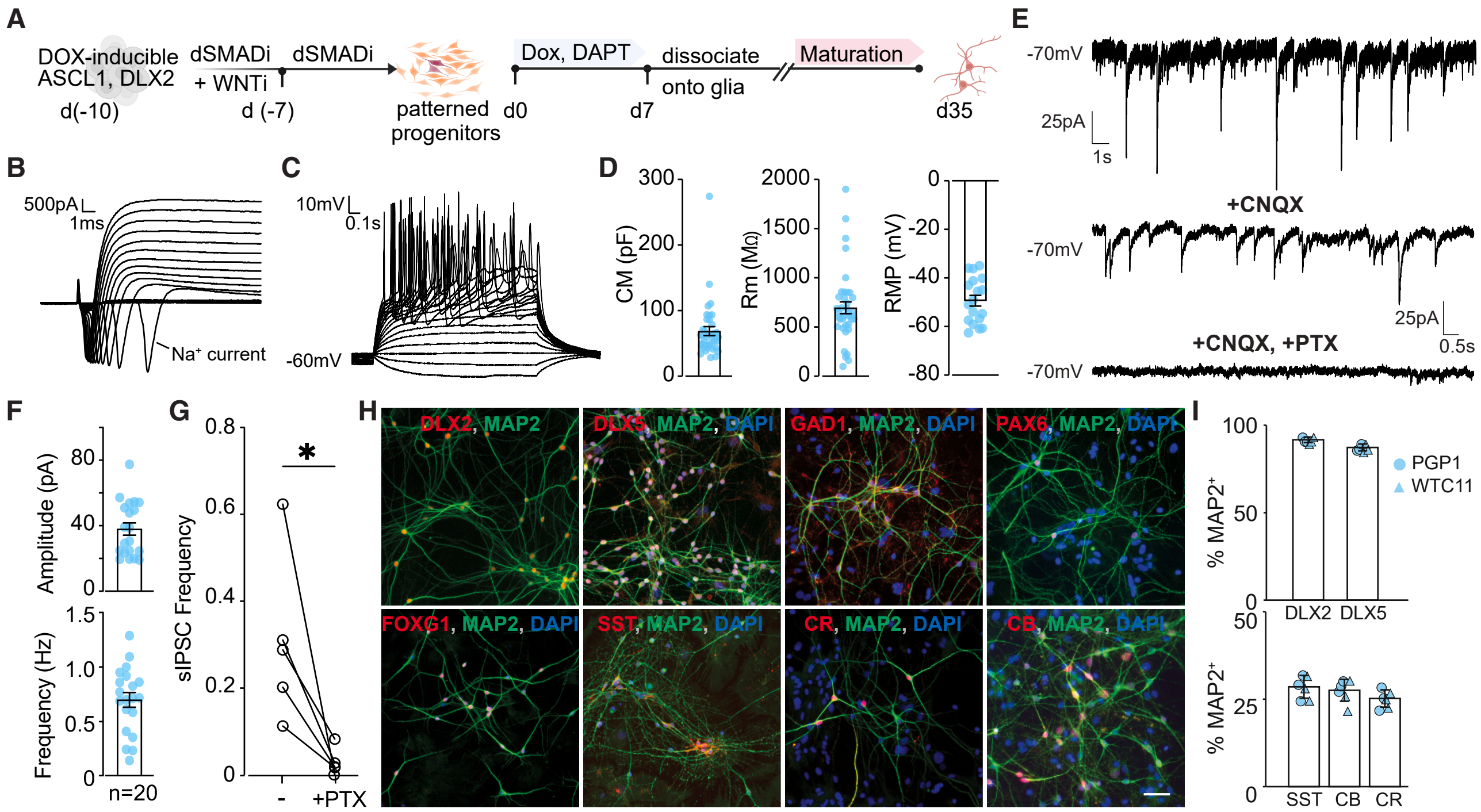

Figure 2. Functional and molecular characterization of patterned iNs

(A) Differentiation timeline.
(B) Voltage-clamp recording showing inward voltage-gated sodium currents.
(C) Current-clamp recording showing repetitive action-potential firing.
(D) Membrane capacitance (Cm; $n = 38$), input resistance (Rm; $n = 38$), and resting membrane potential (RMP; $n = 18$); each dot represents one neuron.
(E) Representative spontaneous IPSC (sIPSC) traces at −70 mV under baseline conditions, with the AMPA-receptor antagonist CNQX, and with CNQX plus the $GABA_a$-receptor antagonist picrotoxin (PTX).
(F) Quantification of sIPSC amplitude and frequency (n = 20 cells).
(G) IPSC frequency before and after PTX application (PTX significantly reduces event frequency; $p < 0.05$).
(H) Immunostaining of $MAP2^+$ neurons expressing DLX2, DLX5, GAD1, FOXG1, PAX6, SST, CR, and CB. Scale bars, 50 μm.
(I) Quantification of the proportion of $MAP2^+$ neurons expressing DLX2 and DLX5, and the subtype-associated markers SST, CB, and CR, across two iPSC donors, PGP1 and WTC11, with three independent differentiations per donor. Each point represents one independent differentiation. Data are shown as mean ± SEM, except in (I), where data are shown as mean ± SD across independent differentiations. See also Figures S4–S6.

across two iPSC lines and three independent differentiations per line (Figure 2I), providing protein-level validation of the transcriptomically defined GABAergic populations. Analysis of the scRNA-seq data identified a population with PV-lineage-associated molecular features, including expression of *ERBB4*, *SLIT2*, *SHISA9*, *RUNX2*, and fast-spiking-associated potassium channel subunits such as KCNJ3, *KCNAB1*, and *KCNC2* (Figure S4C). PV protein was not detected under these conditions. We therefore interpret the PV-associated transcriptional signature cautiously as a lineage-associated molecular feature rather than evidence of mature PV interneuron identity. This interpretation is consistent with prior reports that PV protein expression can be delayed or difficult to detect in human PSC-derived interneurons, even when cells acquire fast-spiking physiological properties (Bershteyn et al., 2025). Future three-dimensional morphological reconstructions may further clarify whether patterned iGABAs acquire dendritic architectures characteristic of specific interneuron subtypes. Together, these results indicate that patterned-iNs generate electrophysiologically active and synaptically competent GABAergic neurons within five weeks *in vitro*. Direct comparison with TF-only PSC-iGABA neurons revealed comparable membrane and synaptic properties (Figure S6), indicating that developmental patterning expands the molecular diversity of GABAergic neurons without altering core functional neuronal properties.

To determine whether patterned-iNs can survive, mature, and integrate into neural circuits *in vivo*, EGFP-labeled

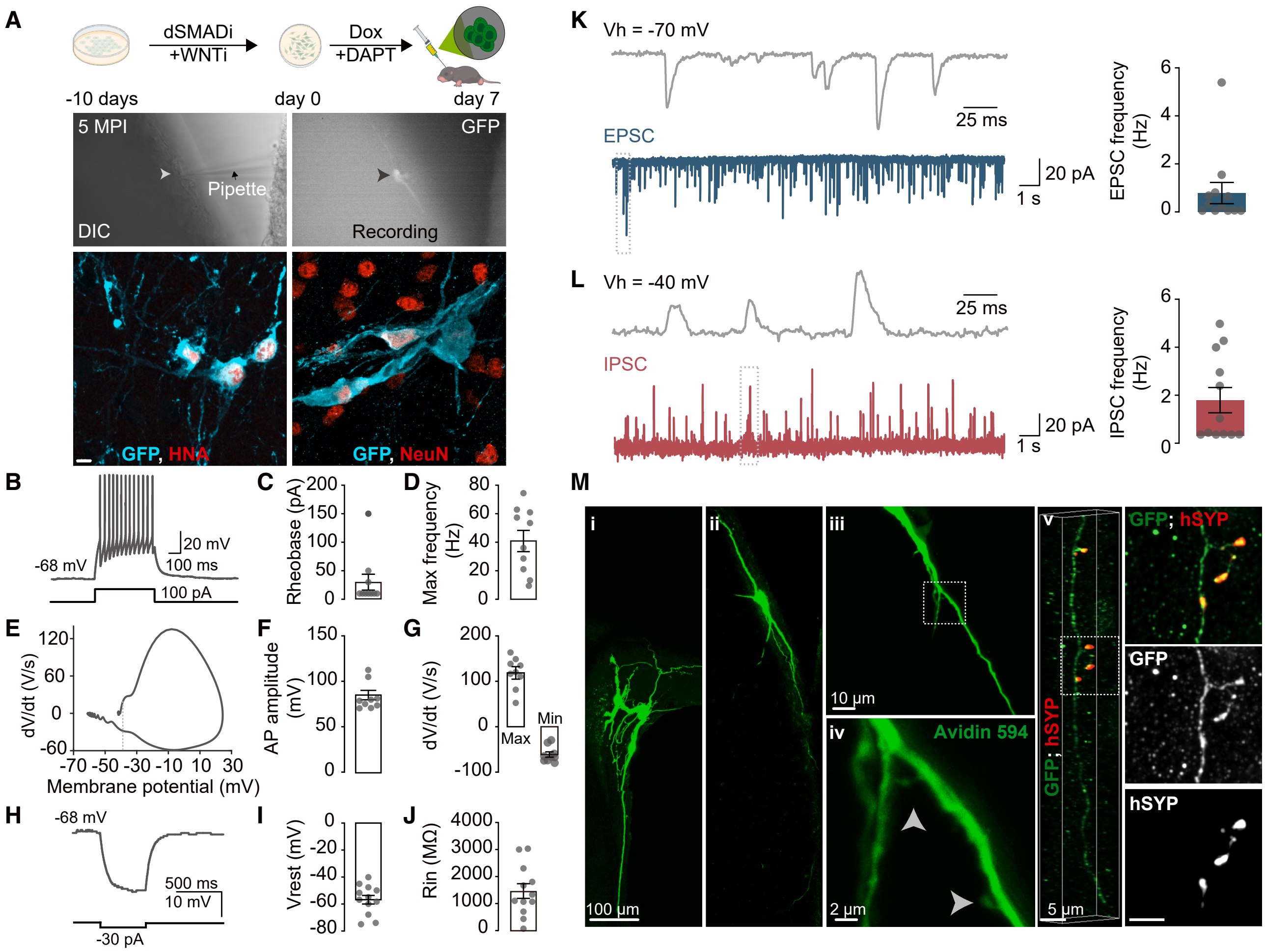

Figure 3. Functional and morphological characterization of transplanted human patterned-iNs

(A) Experimental timeline: patterned iPSCs (10 days dSMADi/WNTi, 7 days Dox/DAPT) were transplanted into P2 mouse brain and analyzed ~5 months post-injection (MPI). Representative differential interference contrast (DIC)/fluorescence images show patch-clamp recording from GFP$^+$ human neurons; HNA and NeuN confirm human neuronal identity. Scale bars, 5 μm.

(B–D) Current-clamp recordings demonstrating evoked action potentials, with quantification of rheobase and maximal firing frequency (*n* = 10 cells, 3 mice).

(E–G) Phase-plane analysis and group data for AP amplitude and dV/dt.

(H–J) Hyperpolarizing responses, resting membrane potential, and input resistance (*n* = 12 cells, 3 mice).

(K and L) Representative spontaneous EPSCs and IPSCs with frequency quantification (*n* = 12 cells each).

(M) Biocytin reconstruction of GFP$^+$ neurons and human synaptophysin puncta associated with GFP$^+$ neurites, consistent with presynaptic specialization. Scale bars as indicated.

Data shown as mean ± SEM. See also Figure S7 and Table S3.

patterned-iNs were transplanted into the periventricular region of neonatal Rag2$^{-/-}$ mice. At five months post-transplantation, human nuclear antigen (HNA)-positive cells were concentrated near the injection site and differentiated into NeuN$^+$ neurons (Figures 3A and S7). Whole-cell patch-clamp recordings from GFP$^+$ human neurons in acute cortical slices revealed robust electrophysiological activity. Across 12 recorded cells from three animals, depolarizing current injections elicited action potentials, with a mean rheobase of 30 ± 14 pA and firing frequencies up to 42 ± 8 Hz (Figures 3B–3D; Table S3). Passive membrane properties were consistent with functionally active, maturing interneurons (Figures 3E–3J). Importantly, transplanted neurons received spontaneous excitatory and inhibitory synaptic inputs, indicating that they can receive synaptic input from host cortical circuits (Figures 3K and 3L; Table S3). In addition, human-specific synaptophysin staining revealed presynaptic puncta associated with GFP$^+$ human neurons

(Figure 3M), suggestive of presynaptic specialization by transplanted neurons. Transplanted cells also exhibited complex morphologies with elaborated dendritic arbors.

Neuropsychiatric and neurological disease gene enrichment across GABAergic iN types

To evaluate the disease relevance of different GABAergic iN populations, we performed gene set enrichment analysis using DisGeNET (Pinero et al., 2020) on shared and condition-specific genes expressed in PSC- and patterned-iGABAs. Genes shared between PSC- and patterned-iGABAs were strongly enriched for risk genes associated with various brain disorders, including intellectual disability, neurodevelopmental disorders (NDDs), schizophrenia, epilepsy, and bipolar disorder (Figure S8A; Table S4; q value ≤ 0.05). Notably, genes specific to patterned iNs were also significantly enriched for psychiatric and neurological disease annotations, whereas genes specific to PSC-iNs were preferentially enriched for non-neural disease categories (Figures S8B and S8C). Consistent with this observation, patterned-iN-specific genes exhibited robust enrichment for ASD risk genes curated in the Simons Foundation Autism Research Initiative (SFARI) database (odds ratio = 2.50; Benjamini-Hochberg (BH)-corrected $p = 1.05 \times 10^{-13}$) (Abrahams et al., 2013). These findings indicate that although both PSC- and patterned iNs capture disease-associated gene networks, patterned iNs show closer alignment with neural lineages, suggesting advantages for modeling neurological disorders. To further assess disease relevance, we incorporated comparative transcriptomic mapping using scRNA-seq data from postmortem brain tissue of individuals with AD and ASD (Gabitto et al., 2024; Velmeshev et al., 2019). These analyses revealed overlap between patterned-iNs and disease-associated interneuron signatures observed in these datasets (Figures S8D and S8E), supporting the relevance of patterned-iNs for modeling disease-linked inhibitory neuron phenotypes. Together, these analyses indicate that regional specification of *in vitro*-derived neurons influences the disease-relevant gene networks represented, providing a framework for selecting differentiation strategies tailored to specific neurological or psychiatric disease contexts.

Gene regulatory networks underlying diverse GABAergic-induced neurons

To explore the regulatory mechanisms underlying GABAergic neuron identities generated by different differentiation strategies, we applied Single-Cell Regulatory Network Inference and Clustering (SCENIC), which infers TF-centered regulons and their target gene networks from scRNA-seq data (Aibar et al., 2017). This analysis identified both shared and strategy-specific regulons across PSC-iGABAs and patterned-iGABAs, revealing distinct regulatory architectures associated with each differentiation approach (Figure S9). At the global level, regulons associated with PSC-iGABAs included well-recognized determinants of interneuron specification and migration, such as DLX2 and DLX5. Regulons linked to 7D-patterned-iGABAs, such as NR2F1 (COUP-TFI), NR2F2 (COUP-TFII), and PAX6, are recognized for their roles in the differentiation of specific interneuron subtypes, including those originating from the LGE and CGE (Hu et al., 2017; Schmitz et al., 2022).

Because relatively few GABAergic neurons were recovered following 3-day DOX induction, subsequent analyses focused on PSC-iNs and 7D-patterned iNs. Neurons were subdivided into molecular clusters, and regulons were ranked using the Regulon Specificity Score (RSS) to identify cluster-enriched regulatory programs (Figures 4A and 4B). Within the PSC-iGABAs, prominent regulons included FOXA1, FOXA2, and PBX1, TFs associated with hypothalamic regionalization, neurogenesis, and neuroendocrine function. FOXA1 and FOXA2 emerged as primary regulons in cluster 1, consistent with their roles in embryonic hypothalamic development and metabolic regulation (Kim et al., 2022). PBX1 has similarly been implicated in hypothalamic pathways involved in hormonal regulation (Rodriguez-Zas et al., 2023). We also showed the expression patterns of these top-ranked regulators in corresponding neuronal clusters (Figure 4A). Consistent with our observations that subsets of PSC-iNs exhibit striatal-like features, several highly expressed TFs associated with striatal and hypothalamic differentiation and function were identified, including ONECUTs, MEIS2, and ISL1 (Fragkouli et al., 2009; Shi et al., 2021).

In contrast, subclusters of patterned-iGABAs were enriched for a distinct set of regulons associated with neural fate specification and migration, including *NR2F1/2*, *DLX6*, *RARB*, *POU2F2*, *TCF12*, and *PAX6* (Figure 4B). Notably, NR2F1, NR2F2, and PAX6 are expressed in GE and play central roles in the development of cortical and ventral forebrain inhibitory neurons. Compared with PSC-iNs, patterned-iNs exhibited more homogeneous regulatory programs across clusters, reflected by broader sharing of key regulons and reduced cluster-specific segregation (Figure 4C). Together, these findings demonstrate that differentiation strategy shapes GABAergic neuron identity through distinct gene regulatory networks, highlighting how TF-based induction and extrinsic patterning cues engage overlapping yet distinct regulatory programs.

Distinct lineage-specific gene regulatory networks induced by ASCL1 and DLX2 in PSCs and patterned progenitors

To investigate how the chromatin state of the starting population influences the neuronal identities specified by ASCL1 and DLX2, we performed simultaneous

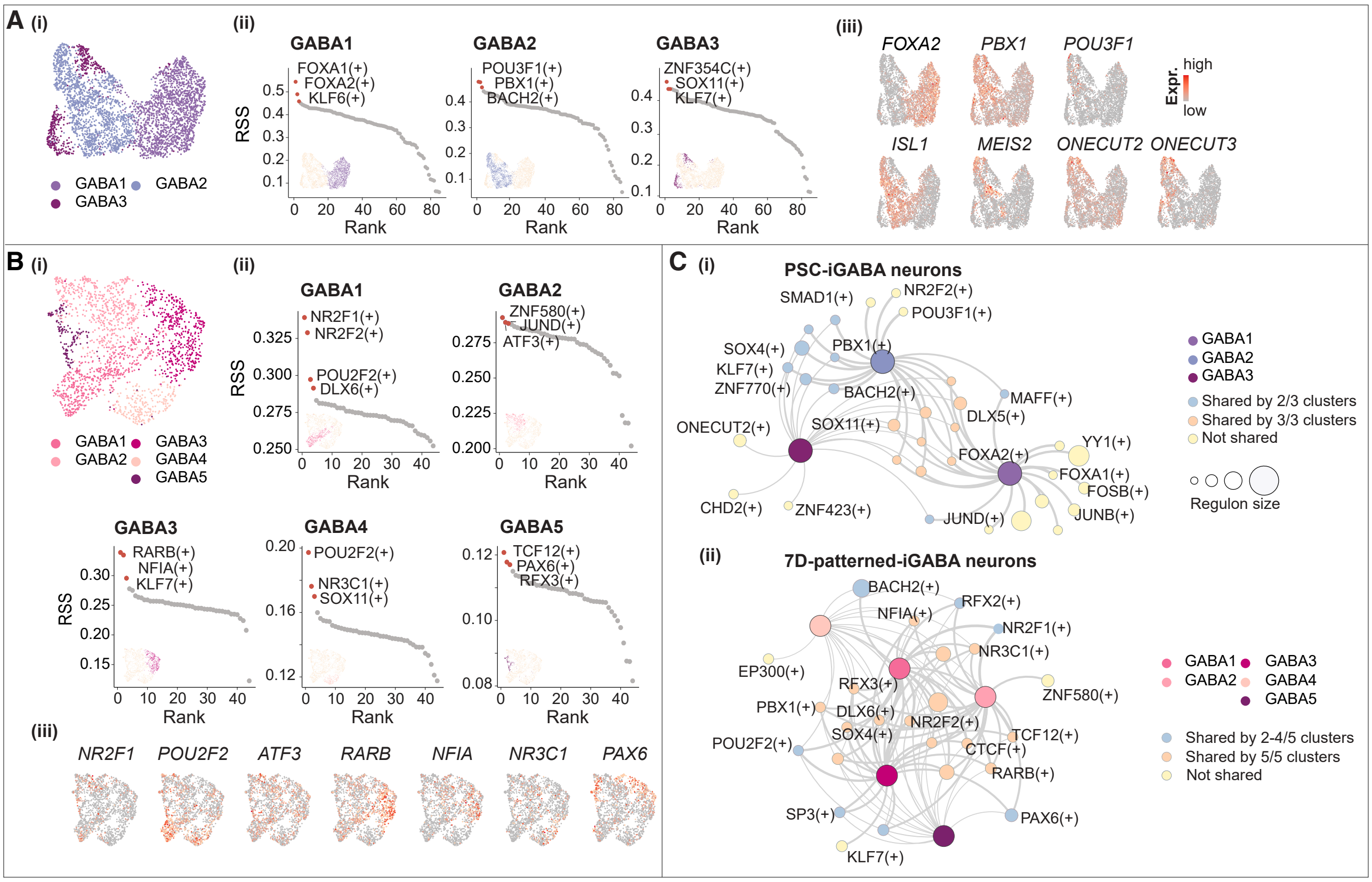

Figure 4. Gene regulatory networks (GRNs) of PSC- and patterned-iNs

(A and B) GRN analysis of PSC-iNs (A) and patterned-iNs (B), showing cluster identities, ranked regulons, and UMAP visualization of top TFs.

(C) Cluster-specific regulatory modules; node size reflects target number and edge thickness indicates association strength.

See also Figure S9.

high-throughput ATAC and RNA expression with sequencing (SHARE-seq) (Ma et al., 2020) on PSCs, patterned progenitors, and cells at early stages following ASCL1 and DLX2 induction, and obtained high-quality data from 23,434 cells (Figure 5A). This approach enabled simultaneous profiling of chromatin accessibility and gene expression within individual cells, providing a comprehensive view of the regulatory landscape underlying neuronal differentiation. Dimensionality reduction (latent semantic indexing [LSI] for assay for transposase-accessible chromatin using sequencing [ATAC-seq], principal component analysis [PCA] for RNA sequencing [RNA-seq]) and UMAP visualization showed strong concordance between chromatin accessibility and transcriptional states, revealing coherent trajectories that reflect the progression of cell states over time and segregate according to cell type of origin (Figure 5B).

Pseudotime reconstruction revealed distinct differentiation trajectories between neurons generated directly from PSCs and those generated after small-molecule patterning (Figure 5C). The patterned cells showed an intermediate neural progenitor-like state, marked by expression of *SOX2*, *NES*, *SOX11*, and *PAX6* (Figure S10). Following ASCL1/DLX2 induction, genes associated with interneuron development, such as *OTX2*, *PROX1*, *TCF12*, and *RELN*, were robustly induced in the patterned progenitors and, to a lesser extent, in PSCs. This transition progressed rapidly toward a GABAergic neuronal identity, marked by *GAD1*, *GAD2*, and *DLX6*, alongside induction of neuronal differentiation genes (e.g., *DCX*, *TUBB3*, *RBFOX3*) and neurotransmitter receptor genes. To complement these analyses and assess lineage specificity, we examined canonical markers of excitatory neuronal and glial lineages in both PSC-iNs and patterned-iNs. Canonical markers of excitatory neurons (*TBR1*, *EMX1*, *SLC17A6/7*) and glial lineages (*GFAP*, *AQP4*, *OLIG1/2*, *MBP*) were largely absent across iGABAs (Figure S11). *MBP* showed modest induction in PSC-iNs, suggesting a slightly higher propensity for glial gene activation in PSC-derived cells. Genes active at later

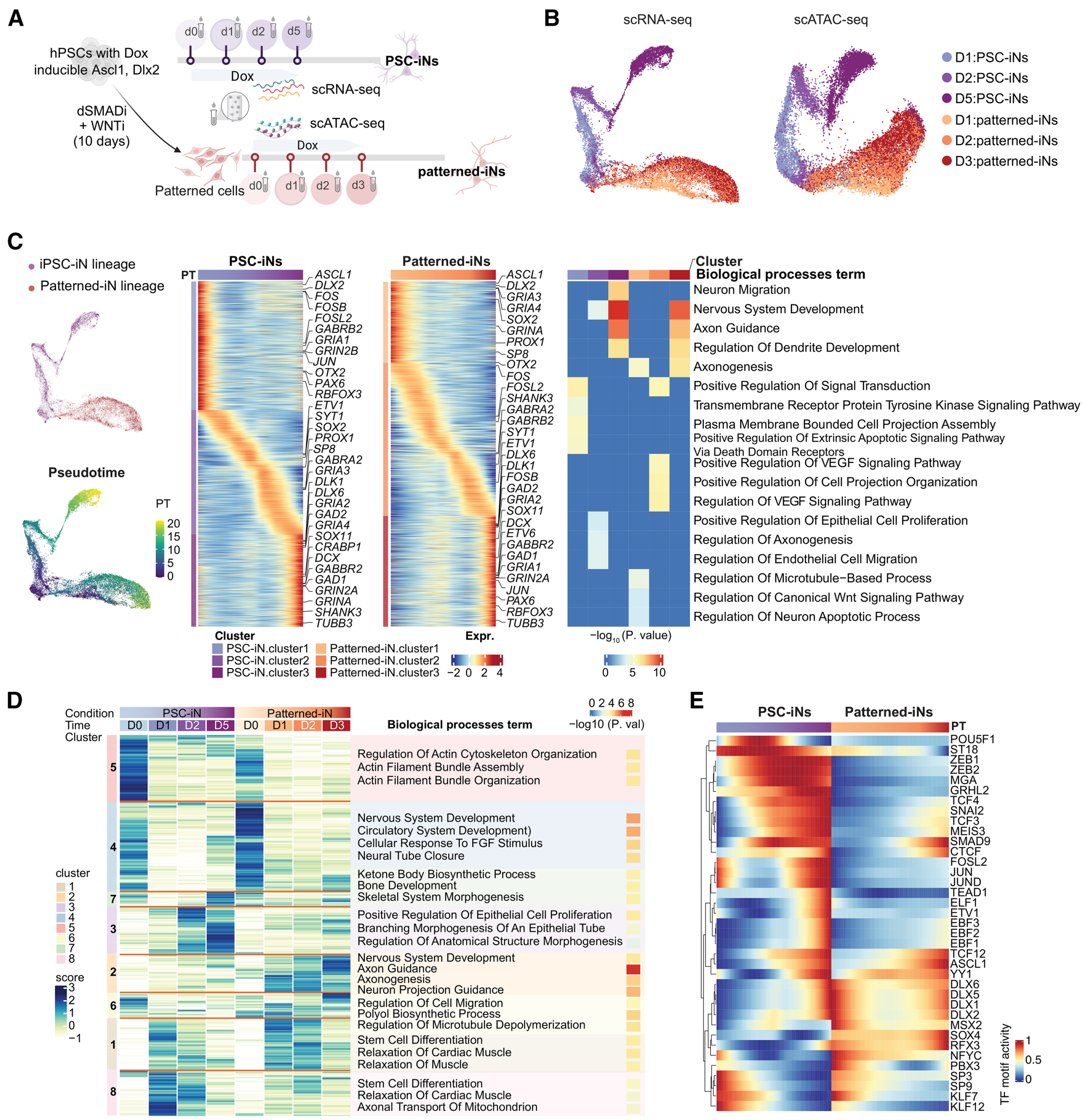

Figure 5. Chromatin and transcriptional dynamics of iN specification
(A) SHARE-seq workflow.
(B) UMAP projections based on gene expression (left) and chromatin accessibility (right), colored by stage and method.
(C) scRNA-seq trajectory with Slingshot pseudotime (left), gene expression heatmap across pseudotime (middle), and GO enrichment of stage-specific genes (right).
(D) Chromatin accessibility heatmap across pseudobulk samples with GO enrichment of cluster-associated accessible regions.
(E) TF motif activity across pseudotime bins.
See also Figures S10 and S11.

pseudotime stages were enriched for Gene Ontology (GO) terms related to neural morphogenesis, migration, and development (Figure 5C). In contrast, early genes in the PSC-iN trajectory were enriched for non-neuronal processes, whereas early patterned-iN genes were enriched for neurogenic signaling pathways, including WNT and VEGF signaling.

Analysis of chromatin accessibility revealed state-dependent remodeling during TF induction (Figure 5D). Regions accessible in PSCs and patterned NPC-like cells but rendered largely inaccessible after ASCL1 and DLX2 induction were primarily associated with non-neuronal processes (clusters 4, 5), whereas newly accessible regions were enriched for neurogenesis (cluster 2). Transiently

accessible regions were linked to stem cell differentiation (clusters 1, 8), while regions uniquely activated along the PSC-iN trajectory were occasionally associated with "off-target" regulator programs (clusters 3, 7). These findings indicate that patterned progenitors undergo more lineage-constrained chromatin remodeling, whereas PSCs exhibit broader regulatory plasticity during early differentiation, highlighting the nuanced interplay between epigenetic and transcriptional regulation in shaping cell fate decisions.

To further define regulatory dynamics, we correlated TF expression with motif accessibility across pseudotime (Figure 5E). This analysis identified 37 TF motifs across the differentiation trajectories. Both differentiation pathways showed strong activity of ASCL1 and DLX motifs, reinforcing their central roles in cell-fate determination. Motifs corresponding to TFs with established roles in neural development and function, including TCF12 (Singh et al., 2022), CTCF (Elbert et al., 2019), SP3 (Yamakawa et al., 2017), SP9 (Liu et al., 2019), and KLF7 (Laub et al., 2001; Moore et al., 2009b), were active in both trajectories. In contrast, motifs associated with pluripotency and lineage plasticity, such as POU5F1 (OCT4), MGA (Qin et al., 2021), SNAI2 (Zhou et al., 2019), TCF3 (Howard et al., 2014), and MEIS3 (Schulte and Geerts, 2019), were preferentially enriched in the PSC-iN trajectory. These results indicate that ASCL1 and DLX2 engage a more constrained transcriptional network in patterned progenitors, promoting a defined path toward GABAergic identity, whereas PSCs retain broader regulatory flexibility that permits activation of additional TF programs.

Disease modeling potential of patterned iGABAs

To determine whether patterned GABAergic iNs can reveal disease-relevant phenotypes, we modeled ADNP syndrome, an NDD frequently associated with ASD and caused by mutations in the ADNP gene (Breen et al., 2020; Helsmoortel et al., 2014). The recurrent p.Tyr719* variant is a truncating mutation in the terminal exon that escapes nonsense-mediated decay and produces a stable truncated protein. This truncated protein retains CHD4 and DNA-binding capacity but lacks interaction with HP1, consistent with a mechanism distinct from simple haploinsufficiency suggested by ADNP mouse studies (Ostapcuk et al., 2018). In patients, class II ADNP mutations, including p.Tyr719*, are associated with hypermethylation episignatures and more severe neurodevelopmental phenotypes (Bend et al., 2019; Breen et al., 2020), highlighting the importance of modeling the specific human mutation. Despite the central role of GABAergic interneurons in excitation-inhibition balance, the impact of ADNP mutations on human inhibitory neurons has not been directly examined.

To model the p.Tyr719* variant, we used CRISPR-Cas9 to introduce the mutation into two independent human iPSC lines (PGP1 and WTC11). In parallel, a hemagglutinin (HA) tag was inserted at the 3′ end of the endogenous *ADNP* locus or immediately following the p.Tyr719* truncation to enable detection of full-length and truncated ADNP proteins. As shown in Figure S12, an ADNP antibody recognizing a region between residues 1,050 and the C terminus detected full-length ADNP in both unedited and HA-tagged control cells but not the truncated p.Tyr719* protein, whereas the truncated protein was readily detected using an HA antibody. Consistent with heterozygosity, levels of full-length ADNP were reduced in heterozygous p.Tyr719* mutant lines relative to unedited controls.

Patterned-iNs were generated from heterozygous and homozygous p.Tyr719* iPSCs and corresponding isogenic controls (six lines total) and analyzed by scRNA-seq after five weeks of glial co-culture. Following quality control, the WTC11 homozygous sample was excluded for low library quality (see STAR Methods). Patterned-iNs derived from independent genetic backgrounds showed high reproducibility (Figure S13A). Transcriptomic profiles of heterozygous and homozygous mutants were highly correlated (Figures 6A and 6B), and mutant cells from both genotypes were therefore combined to increase statistical power. Neurons from both conditions robustly expressed MAP2 and RBFOX3 (NeuN) (Figure S13B), and the vast majority of neurons were GABAergic (98.62% in wild-type; 97.28% in mutants).

To assess cellular heterogeneity, we performed unsupervized clustering, which revealed clear segregation between mutant and wild-type neurons and significant transcriptional differences. (Figure 6C; Table S5). Clusters 0 and 1 were enriched for mutant cells, whereas cluster 2 was predominantly wild-type (Figure 6C). Pseudotime analysis using Monocle3 identified cluster 0 mutant neurons as the least mature population (Figure 6D), consistent with elevated expression of the progenitor marker NESTIN (*NES*). A continuous differentiation trajectory within the mutant population was also observed and independently validated using Slingshot (Figure S13C). Along this trajectory, mutant neurons progressively upregulated genes involved in synaptic signaling and neuronal maturation, including receptor subunits (*GABRB2*, *GRIA2*, *GRID2*), synaptic components (*SYN3*, *DLG2*, *NRXN3*), and guidance molecules (*ROBO1*, *NRP2*) (Figure S13D; Table S6).

To define molecular alterations associated with the mutation, we performed pseudobulk differential expression analysis between mature mutant and wild-type neurons (Figure 6E; Table S7). Mutant cells exhibited both downregulated and upregulated transcriptional programs. Notably, genes associated with CGE-derived interneurons (NR2F2, PROX1) and LGE-derived interneurons (FOXP2, CALB1)

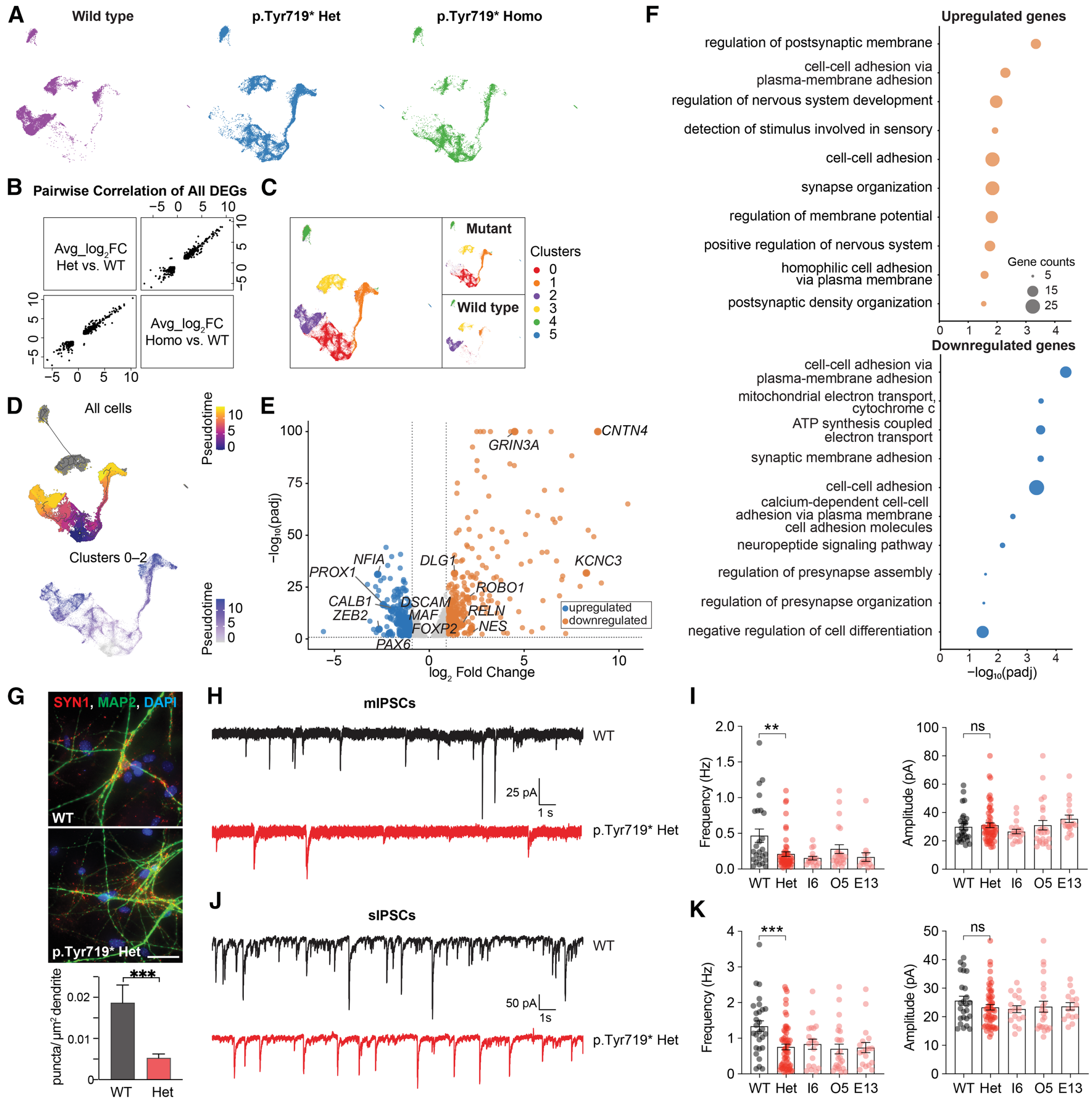

Figure 6. ADNP p.Tyr719* alters interneuron maturation and impairs inhibitory synaptic transmission
(A) UMAP colored by ADNP genotype (WT, heterozygous, homozygous).
(B) Correlation heatmap of differentially expressed genes (DEGs) between WT and ADNP p.Tyr719* samples.
(C) Combined UMAP highlighting genotype-enriched clusters; right, split by genotype.
(D) Pseudotime analysis of all cells and clusters 0–2.
(E) Volcano plot of DEGs (adjusted $p < 0.05$; $|\log_2 FC| > 1$).
(F) GO enrichment of DEGs.
(G) SYN1 immunostaining and puncta density quantification. Scale bars, 50 µm.
(H and I) mIPSC traces and quantification (WT $n = 25$; Het $n = 61$, three batches; clones I6, O5, E13 shown separately).
(J and K) sIPSC traces and pooled quantification (WT $n = 28$; Het $n = 61$). Data shown as mean ± SEM. Statistics by unpaired t test or one-way ANOVA, as appropriate. ADNP Het neurons from individual clones (I6, O5, E13) are also shown separately in (I) and (K).
See also Figures S12–S14 and Tables S5–S10.

were significantly downregulated, together with PAX6, which contributes to both CGE and LGE interneuron development. Conversely, *RELN*, produced by specific subsets of cortical and hippocampal GABAergic interneurons, was upregulated (Pesold et al., 1998). GO highlighted enrichment of pathways related to synapse organization, *trans*-synaptic signaling, membrane potential regulation, and neuronal projection development (Figure 6F; Table S8). To capture

coordinated transcriptional changes associated with the mutation, we performed weighted gene co-expression network analysis (WGCNA) across clusters 0–2. To reduce sparsity while preserving cellular heterogeneity, we first generated metacells (Baran et al., 2019), representing aggregated cells with similar transcriptional profiles (Figure S14A). Network analysis identified 25 gene co-expression modules (Figure S14B; Table S9). Modules associated with mutant neurons were enriched for processes including translational initiation, neuronal development, membrane potential regulation, and chemical synaptic transmission (Figure S14C).

Finally, we tested whether these transcriptional alterations corresponded to functional deficits using whole-cell patch-clamp recordings of patterned GABAergic interneurons derived from three independent mutant clones (I6, O5, E13) and matched controls. Mutant neurons exhibited significantly reduced spontaneous and miniature inhibitory postsynaptic current (sIPSC and mIPSC) frequencies, without changes in amplitude, indicating impaired presynaptic inhibitory transmission (Figures 6G–6K; Table S10). These deficits were consistent across three independent differentiation batches. Collectively, these results show that the ADNP p.Tyr719* mutation disrupts interneuron maturation and subtype-associated transcriptional programs and impairs inhibitory synaptic transmission in human patterned GABAergic neurons, highlighting patterned-iNs as a platform for identifying mutation-specific mechanisms in NDDs.

DISCUSSION

Differentiating human stem cells into interneuron populations with defined neurotransmitter and regional identities offers a promising platform for investigating disease mechanisms and therapeutic strategies. Here, we describe a rapid and reproducible method for generating GABAergic neurons by integrating our TF-mediated PSC differentiation approach with brief developmental patterning achieved by SMAD and WNT inhibition. Similar to direct TF-mediated conversion by ASCL1 and DLX2, this strategy efficiently produces cultures highly enriched for GABAergic neuronal identity. Using single-cell transcriptomic profiling, we systematically compared neurons generated through TF-only induction, small-molecule patterning, and combinatorial patterning plus TF approaches, revealing that each strategy produces GABAergic neurons with overlapping but distinct regional identities, developmental trajectories, and gene regulatory programs. Many neurodevelopmental and neuropsychiatric disorders are linked to dysfunction in particular interneuron subtypes and developmental programs. Consequently, selecting a differentiation strategy should consider the spatial, temporal, and cell-type-specific expression patterns of disease-associated genes. The increasing availability of human single-cell brain datasets further facilitates the rational selection of differentiation strategies by enabling benchmarking against *in vivo* references. In this framework, TF-only PSC-iGABAs may be useful when speed, scalability, and broad inhibitory neuron production are prioritized, whereas patterned-iGABAs may be preferred when forebrain/ganglionic-eminence-associated molecular programs or disease-relevant cortical interneuron signatures are central to the biological question. Consistent with this concept, we show that patterned GABAergic neurons capture disease-relevant transcriptional programs and can be used to investigate the pathobiology of ADNP syndrome.

SMAD and WNT inhibitors can induce forebrain lineages from human PSCs, including excitatory and inhibitory neurons. When combined with SHH signaling, these progenitors can be ventralized to produce cortical interneuron lineages, although their maturation typically requires extended differentiation. In contrast, induction of ASCL1 and DLX2 in PSCs exposed to SMAD and WNT inhibitors rapidly generates GABAergic neurons without requiring SHH activation. These neurons display signatures of multiple brain regions but are biased toward cortical lineages, whereas PSC-derived iGABAs generated without patterning show stronger hypothalamic identities and enrichment for TF regulons associated with hypothalamic and striatal development. Patterned-iGABAs, by contrast, show enrichment for regulons linked to neural fate determination and neuronal migration, reflecting distinct developmental trajectories between the two differentiation contexts. Following transplantation into neonatal mouse brains, patterned-iGABAs survived long term and differentiated into $NeuN^+$, functionally active neurons but remained largely localized near the injection site. This limited migration is consistent with previous observations of PSC-iGABAs (Yang et al., 2017) and suggests that rapid TF-mediated conversion bypasses the migratory developmental stages more faithfully captured by small-molecule MGE-patterning protocols, which can yield migratory pallial interneurons (Bershteyn et al., 2025). Our approach therefore prioritizes the rapid and reproducible generation of molecularly diverse GABAergic neurons over the faithful recapitulation of interneuron migratory development. The relatively high input resistance of patterned-iGABA neurons is intermediate between values reported for human fetal cortical neurons and mature adult human interneurons, consistent with an intermediate developmental stage rather than a functional defect (Moore et al., 2009a; Lee et al., 2023).

The differences observed between PSC-iGABAs and patterned-iGABAs likely reflect the distinct epigenetic and

transcriptional landscapes of their starting cell populations. PSCs possess a more open chromatin configuration, enabling broader TF binding and greater lineage plasticity, whereas NPC-like cells generated through SMAD and WNT inhibition exhibit chromatin states already primed for neural differentiation. Although ASCL1 acts as a pioneering factor capable of accessing closed chromatin, its activity is strongly influenced by cellular context. In PSCs, ASCL1 and DLX2 can activate a broader range of transcriptional programs, leading to more heterogeneous gene expression patterns, whereas in patterned progenitors, these factors primarily reinforce existing neural programs, resulting in more streamlined differentiation trajectories. Consistent with single-cell analyses of developing interneurons *in vivo*, patterned-iGABAs expressing subtype-associated markers appear to exist along a transcriptomic continuum rather than as sharply defined populations, with subtype boundaries becoming progressively refined during maturation and circuit integration (Lim et al., 2018; Mayer et al., 2018; Shi et al., 2021). Accordingly, atlas mapping in this study reflects transcriptomic resemblance to reference populations rather than definitive subtype assignment. The molecular heterogeneity observed in our cultures likely reflects both diversity within the starting progenitor population and variability in how ASCL1 and DLX2 engage the accessible chromatin landscape during fate conversion.

Importantly, molecular heterogeneity in this system was reproducible rather than stochastic. Reference-based mapping across three independent iPSC lines showed highly concordant subtype-associated population structure, and protein-level quantification across two iPSC lines and independent differentiations confirmed consistent generation of DLX2/DLX5-positive GABAergic neurons and SST-, CR-, and CB-marker-positive populations. We therefore interpret SST-, CR-, CB-, and PV-lineage-associated signatures as evidence of subtype-level molecular programs, rather than as definitive assignments to mature interneuron subtypes.

Finally, we applied patterned human GABAergic neurons to model ADNP syndrome, focusing on the recurrent p.Tyr719* truncating mutation. ADNP functions as a chromatin-associated regulator that interacts with multiple partners. Together with CHD4 and HP1γ, ADNP forms the CHD4-ADNP-HP1 (ChAHP) complex, which represses transcription at specific genomic loci (Ostapcuk et al., 2018). In mouse models, ADNP loss disrupts neuronal specification and synaptic development (Cho et al., 2023; Ostapcuk et al., 2018). However, many pathogenic human mutations occur in the terminal exon and are predicted to escape nonsense-mediated decay. Using endogenous HA tagging, we directly demonstrated stable expression of the truncated ADNP protein from the native locus, supporting the idea that terminal truncating variants may not be fully modeled by simple haploinsufficiency. Consistent with this possibility, the p.Tyr719* mutation altered interneuron subtype-associated gene expression and impaired inhibitory synaptic transmission in patterned GABAergic neurons. These frequency reductions, recorded directly from GABAergic neurons, contrast with the amplitude changes reported in excitatory neuron recordings from ADNP mouse models, suggesting an interneuron-autonomous presynaptic mechanism. Notably, heterozygous and homozygous mutant neurons showed highly similar transcriptional profiles, suggesting that the truncated protein may interfere with full-length ADNP function rather than simply reducing gene dosage. The observed upregulation of CHD4 in mutant cells further raises the possibility that truncated ADNP perturbs chromatin regulatory complexes through altered protein interactions (Figure S12B). Although a complete mechanistic dissection will require additional studies, these findings provide a proof-of-principle demonstration that human stem cell-derived interneuron models can reveal mutation-specific cellular phenotypes not captured in traditional loss-of-function systems.

Overall, our study expands the toolkit for modeling human brain disorders by demonstrating that PSC-iNs and patterned-iNs represent complementary neuronal models with distinct regional and developmental properties. Although definitive subtype classification will require future studies integrating detailed morphology, circuit-level connectivity mapping, and subtype-specific electrophysiology, these systems provide a flexible platform for dissecting the molecular mechanisms underlying diverse neurodevelopmental and neuropsychiatric diseases. Accordingly, patterned-iGABAs should be regarded as molecularly patterned human GABAergic neurons rather than as fully mature interneuron-subtype equivalents.

RESOURCE AVAILABILITY

Lead contact

Further information and requests for resources and reagents should be directed to and will be fulfilled by the Lead Contact, Nan Yang (nan.yang1@mssm.edu).

Materials availability

The plasmids and cell lines generated in this study will be made available on request upon the completion of a material transfer agreement (MTA).

Data and code availability

The scRNA-seq and SHARE-seq data have been deposited at GEO and are publicly available as of the date of publication. Accession numbers are listed in the key resources table. This paper does not report original code. Any additional information required to reanalyze the data reported in this paper is available from the lead contact upon request.

ACKNOWLEDGMENTS

This work was supported by NIH awards 5R21MH119580 (to N.Y.), 5R01NS116057 (to N.Y.), R21MH131600 (to J.D.B. and N.Y.), 5R01AA023797 (to Z.P.P), and 5R01MH125528 (to Z.P.P). This study was supported in part by a grant from the Eagles Autism Foundation (to N.Y.), Simons Foundation (SFI-AN-AR-Pilot-00009949) (to J.D.B.), Oxford-Harrington Rare Disease Scholar award (to J.D.B.), ADNP Foundation, and Seattle Foundation. X.W. is supported as a Seaver Foundation Fellow and by NARSAD young investigator grants (31904). R.H. is a Seaver postdoctoral fellow. L.L.B. was supported by the Training Program in Stem Cell Biology fellowship from the New York State Department of Health (NYSTEM-C32561GG). M.Y. is supported by a diversity supplement (3R01MH129372-03S1). M.L. is supported by the NIH F31 Ruth L. Kirschstein National Research Service award (F31NS137751).

This work was partly supported by the computational and data resources and staff expertise provided by Scientific Computing and Data at the Icahn School of Medicine at Mount Sinai, and funded by the clinical and translational science awards (CTSA) grant UL1TR004419 from the National Center for Advancing Translational Sciences. Research reported in this publication also received support from the Office of Research Infrastructure of the National Institutes of Health under award numbers S10OD026880 and S10OD030463. The content is solely the responsibility of the authors and does not necessarily reflect the official views of the National Institutes of Health.

AUTHOR CONTRIBUTIONS

Conceptualization, R.H., L.L.B., J.D.B., S.M., and N.Y.; methodology, R.H., L.L.B., and Y.T.; investigation, R.H., Y.T., B.Z., J.L., X.L., R.D., M.L., G.I., and N.Y.; formal analysis and data visualization, L.L.B., M.Y., P.C., Y.S., J.L., X.L., X.W., A.M.T., and S.M.; writing, R.H., L.L.B., J.L., X.L., X.W., S.M., and N.Y.; supervision, X.W., A.M.T., J.D.B., P.J., Z.P.P., S.M., and N.Y.

DECLARATION OF INTERESTS

The authors declare no competing interests.

DECLARATION OF GENERATIVE AI AND AI-ASSISTED TECHNOLOGIES IN THE WRITING PROCESS

During the preparation of this work, the author(s) used ChatGPT (OpenAI) to assist with improving the readability and language clarity of portions of the manuscript text. After using this tool/service, the author(s) reviewed and edited the content as needed and take(s) full responsibility for the content of the published article.

STAR★METHODS

Detailed methods are provided in the online version of this paper and include the following:

- KEY RESOURCES TABLE
- EXPERIMENTAL MODEL AND STUDY PARTICIPANT DETAILS
  - Animal model
  - Human PSC lines and cell culture
  - Lentivirus production
  - Small molecule-mediated GABAergic neuron generation
  - TF-mediated induced GABAergic neuron generation
  - Patterned induced GABAergic neuron generation
- METHOD DETAILS
  - Primary mouse astrocytes
  - Transplantation and brain slice preparation
  - Electrophysiological recordings from brain slices
  - Whole-cell recordings from cultured neurons
  - Immunofluorescence and imaging
  - Immunoblotting
  - 10× single-cell RNA-seq library preparation and sequencing
  - SHARE-seq library preparation and sequencing
- QUANTIFICATION AND STATISTICAL ANALYSIS
  - 10× single-cell RNA-sequencing data processing and analysis
  - SHARE-seq data processing and analysis
  - Inferring regulatory networks
  - Gene ontology, pathway enrichment and disease-vulnerable cell type analysis
  - WGCNA analysis
  - Statistical analyses

SUPPLEMENTAL INFORMATION

Supplemental information can be found online at https://doi.org/10.1016/j.stemcr.2026.10308.

Received: March 13, 2026
Revised: August 25, 2026
Accepted: August 26, 2026
Published: September 24, 2026

REFERENCES

Abrahams, B.S., Arking, D.E., Campbell, D.B., Mefford, H.C., Morrow, E.M., Weiss, L.A., Menashe, I., Wadkins, T., Banerjee-Basu, S., and Packer, A. (2013). SFARI Gene 2.0: a community-driven knowledgebase for the autism spectrum disorders (ASDs). Mol. Autism *4*, 36. https://doi.org/10.1186/2040-2392-4-36.

Aibar, S., González-Blas, C.B., Moerman, T., Huynh-Thu, V.A., Imrichova, H., Hulselmans, G., Rambow, F., Marine, J.C., Geurts, P., Aerts, J., et al. (2017). SCENIC: single-cell regulatory network inference and clustering. Nat. Methods *14*, 1083–1086. https://doi.org/10.1038/nmeth.4463.

Allison, T., Langerman, J., Sabri, S., Otero-Garcia, M., Lund, A., Huang, J., Wei, X., Samarasinghe, R.A., Polioudakis, D., Mody, I., et al. (2021). Defining the nature of human pluripotent stem cell-derived interneurons via single-cell analysis. Stem Cell Rep. *16*, 2548–2564. https://doi.org/10.1016/j.stemcr.2021.08.006.

Amezquita, R.A., Lun, A.T.L., Becht, E., Carey, V.J., Carpp, L.N., Geistlinger, L., Marini, F., Rue-Albrecht, K., Risso, D., Soneson, C., et al. (2020). Orchestrating single-cell analysis with Bioconductor. Nat. Methods *17*, 137–145. https://doi.org/10.1038/s41592-019-0654-x.

Baran, Y., Bercovich, A., Sebe-Pedros, A., Lubling, Y., Giladi, A., Chomsky, E., Meir, Z., Hoichman, M., Lifshitz, A., and Tanay, A. (2019). MetaCell: analysis of single-cell RNA-seq data using K-nn graph partitions. Genome Biol. *20*, 206. https://doi.org/10.1186/s13059-019-1812-2.

Bend, E.G., Aref-Eshghi, E., Everman, D.B., Rogers, R.C., Cathey, S.S., Prijoles, E.J., Lyons, M.J., Davis, H., Clarkson, K., Gripp, K.W., et al. (2019). Gene domain-specific DNA methylation episignatures highlight distinct molecular entities of ADNP syndrome. Clin. Epigenet. *11*, 64. https://doi.org/10.1186/s13148-019-0658-5.

Bershteyn, M., Zhou, H., Fuentealba, L., Chen, C., Subramanyam, G., Cherkowsky, D., Sevilla, E.S., Hampel, P., Salvatierra, J., Sezan, M., et al. (2025). Human stem cell-derived GABAergic interneuron development reveals early emergence of subtype diversity and gradual electrochemical maturation. Neuron *113*, 3162–3184.e10. https://doi.org/10.1016/j.neuron.2025.06.010.

Bhaduri, A., Sandoval-Espinosa, C., Otero-Garcia, M., Oh, I., Yin, R., Eze, U.C., Nowakowski, T.J., and Kriegstein, A.R. (2021). An atlas of cortical arealization identifies dynamic molecular signatures. Nature *598*, 200–204. https://doi.org/10.1038/s41586-021-03910-8.

Breen, M.S., Garg, P., Tang, L., Mendonca, D., Levy, T., Barbosa, M., Arnett, A.B., Kurtz-Nelson, E., Agolini, E., Battaglia, A., et al. (2020). Episignatures Stratifying Helsmoortel-Van Der Aa Syndrome Show Modest Correlation with Phenotype. Am. J. Hum. Genet. *107*, 555–563. https://doi.org/10.1016/j.ajhg.2020.07.003.

Butler, A., Hoffman, P., Smibert, P., Papalexi, E., and Satija, R. (2018). Integrating single-cell transcriptomic data across different conditions, technologies, and species. Nat. Biotechnol. *36*, 411–420. https://doi.org/10.1038/nbt.4096.

Butt, S.J.B., Sousa, V.H., Fuccillo, M.V., Hjerling-Leffler, J., Miyoshi, G., Kimura, S., and Fishell, G. (2008). The requirement of Nkx2-1 in the temporal specification of cortical interneuron subtypes. Neuron *59*, 722–732. https://doi.org/10.1016/j.neuron.2008.07.031.

Cao, J., Spielmann, M., Qiu, X., Huang, X., Ibrahim, D.M., Hill, A.J., Zhang, F., Mundlos, S., Christiansen, L., Steemers, F.J., et al. (2019). The single-cell transcriptional landscape of mammalian organogenesis. Nature *566*, 496–502. https://doi.org/10.1038/s41586-019-0969-x.

Chen, E.Y., Tan, C.M., Kou, Y., Duan, Q., Wang, Z., Meirelles, G.V., Clark, N.R., and Ma'ayan, A. (2013). Enrichr: interactive and collaborative HTML5 gene list enrichment analysis tool. BMC Bioinf. *14*, 128. https://doi.org/10.1186/1471-2105-14-128.

Chen, J., Bardes, E.E., Aronow, B.J., and Jegga, A.G. (2009). ToppGene Suite for gene list enrichment analysis and candidate gene prioritization. Nucleic Acids Res. *37*, W305–W311. https://doi.org/10.1093/nar/gkp427.

Cho, H., Yoo, T., Moon, H., Kang, H., Yang, Y., Kang, M., Yang, E., Lee, D., Hwang, D., Kim, H., et al. (2023). Adnp-mutant mice with cognitive inflexibility, CaMKIIalpha hyperactivity, and synaptic plasticity deficits. Mol. Psychiatr. *28*, 3548–3562. https://doi.org/10.1038/s41380-023-02129-5.

Ciceri, G., Baggiolini, A., Cho, H.S., Kshirsagar, M., Benito-Kwiecinski, S., Walsh, R.M., Aromolaran, K.A., Gonzalez-Hernandez, A.J., Munguba, H., Koo, S.Y., et al. (2024). An epigenetic barrier sets the timing of human neuronal maturation. Nature *626*, 881–890. https://doi.org/10.1038/s41586-023-06984-8.

Close, J.L., Yao, Z., Levi, B.P., Miller, J.A., Bakken, T.E., Menon, V., Ting, J.T., Wall, A., Krostag, A.R., Thomsen, E.R., et al. (2017). Single-Cell Profiling of an In Vitro Model of Human Interneuron Development Reveals Temporal Dynamics of Cell Type Production and Maturation. Neuron *93*, 1035–1048.e5. https://doi.org/10.1016/j.neuron.2017.02.014.

Di Tommaso, P., Chatzou, M., Floden, E.W., Barja, P.P., Palumbo, E., and Notredame, C. (2017). Nextflow enables reproducible computational workflows. Nat. Biotechnol. *35*, 316–319. https://doi.org/10.1038/nbt.3820.

Dobin, A., Davis, C.A., Schlesinger, F., Drenkow, J., Zaleski, C., Jha, S., Batut, P., Chaisson, M., and Gingeras, T.R. (2013). STAR: ultrafast universal RNA-seq aligner. Bioinformatics *29*, 15–21. https://doi.org/10.1093/bioinformatics/bts635.

Dull, T., Zufferey, R., Kelly, M., Mandel, R.J., Nguyen, M., Trono, D., and Naldini, L. (1998). A third-generation lentivirus vector with a conditional packaging system. J. Virol. *72*, 8463–8471. https://doi.org/10.1128/JVI.72.11.8463-8471.1998.

Eichmüller, O.L., Corsini, N.S., Vértesy, Á., Morassut, I., Scholl, T., Gruber, V.E., Peer, A.M., Chu, J., Novatchkova, M., Hainfellner, J.A., et al. (2022). Amplification of human interneuron progenitors promotes brain tumors and neurological defects. Science *375*, eabf5546. https://doi.org/10.1126/science.abf5546.

Elbert, A., Vogt, D., Watson, A., Levy, M., Jiang, Y., Brûlé, E., Rowland, M.E., Rubenstein, J., and Bérubé, N.G. (2019). CTCF Governs the Identity and Migration of MGE-Derived Cortical Interneurons. J. Neurosci. *39*, 177–192. https://doi.org/10.1523/JNEUROSCI.3496-17.2018.

Feng, X., Gao, Y., Chu, F., Shan, Y., Liu, M., Wang, Y., Zhu, Y., Lu, Q., and Li, M. (2025). Cortical arealization of interneurons defines shared and distinct molecular programs in developing human and macaque brains. Nat. Commun. *16*, 672. https://doi.org/10.1038/s41467-025-56058-8.

Fragkouli, A., van Wijk, N.V., Lopes, R., Kessaris, N., and Pachnis, V. (2009). LIM homeodomain transcription factor-dependent specification of bipotential MGE progenitors into cholinergic and GABAergic striatal interneurons. Development *136*, 3841–3851. https://doi.org/10.1242/dev.038083.

Fu, J.M., Satterstrom, F.K., Peng, M., Brand, H., Collins, R.L., Dong, S., Wamsley, B., Klei, L., Wang, L., Hao, S.P., et al. (2022). Rare coding variation provides insight into the genetic architecture and phenotypic context of autism. Nat. Genet. *54*, 1320–1331. https://doi.org/10.1038/s41588-022-01104-0.

Gabitto, M.I., Travaglini, K.J., Rachleff, V.M., Kaplan, E.S., Long, B., Ariza, J., Ding, Y., Mahoney, J.T., Dee, N., Goldy, J., et al. (2024). Integrated multimodal cell atlas of Alzheimer's disease. Nat. Neurosci. *27*, 2366–2383. https://doi.org/10.1038/s41593-024-01774-5.

Goff, K.M., and Goldberg, E.M. (2021). A Role for Vasoactive Intestinal Peptide Interneurons in Neurodevelopmental Disorders. Dev. Neurosci. *43*, 168–180. https://doi.org/10.1159/000515264.

Goulburn, A.L., Alden, D., Davis, R.P., Micallef, S.J., Ng, E.S., Yu, Q.C., Lim, S.M., Soh, C.L., Elliott, D.A., Hatzistavrou, T., et al. (2011). A targeted NKX2.1 human embryonic stem cell reporter

line enables identification of human basal forebrain derivatives. Stem Cell. *29*, 462–473. https://doi.org/10.1002/stem.587.

Hansen, D.V., Lui, J.H., Flandin, P., Yoshikawa, K., Rubenstein, J.L., Alvarez-Buylla, A., and Kriegstein, A.R. (2013). Non-epithelial stem cells and cortical interneuron production in the human ganglionic eminences. Nat. Neurosci. *16*, 1576–1587. https://doi.org/10.1038/nn.3541.

Helsmoortel, C., Vulto-van Silfhout, A.T., Coe, B.P., Vandeweyer, G., Rooms, L., van den Ende, J., Schuurs-Hoeijmakers, J.H.M., Marcelis, C.L., Willemsen, M.H., Vissers, L.E.L.M., et al. (2014). A SWI/SNF-related autism syndrome caused by de novo mutations in ADNP. Nat. Genet. *46*, 380–384. https://doi.org/10.1038/ng.2899.

Herb, B.R., Glover, H.J., Bhaduri, A., Colantuoni, C., Bale, T.L., Siletti, K., Hodge, R., Lein, E., Kriegstein, A.R., Doege, C.A., and Ament, S.A. (2023). Single-cell genomics reveals region-specific developmental trajectories underlying neuronal diversity in the human hypothalamus. Sci. Adv. *9*, eadf6251. https://doi.org/10.1126/sciadv.adf6251.

Hockemeyer, D., Soldner, F., Cook, E.G., Gao, Q., Mitalipova, M., and Jaenisch, R. (2008). A drug-inducible system for direct reprogramming of human somatic cells to pluripotency. Cell Stem Cell *3*, 346–353. https://doi.org/10.1016/j.stem.2008.08.014.

Howard, J.M., Nuguid, J.M., Ngole, D., and Nguyen, H. (2014). Tcf3 expression marks both stem and progenitor cells in multiple epithelia. Development *141*, 3143–3152. https://doi.org/10.1242/dev.106989.

Hu, J.S., Vogt, D., Lindtner, S., Sandberg, M., Silberberg, S.N., and Rubenstein, J.L.R. (2017). Coup-TF1 and Coup-TF2 control subtype and laminar identity of MGE-derived neocortical interneurons. Development *144*, 2837–2851. https://doi.org/10.1242/dev.150664.

Inan, M., Petros, T.J., and Anderson, S.A. (2013). Losing your inhibition: linking cortical GABAergic interneurons to schizophrenia. Neurobiol. Dis. *53*, 36–48. https://doi.org/10.1016/j.nbd.2012.11.013.

Katsarou, A.M., Moshé, S.L., and Galanopoulou, A.S. (2017). Interneuronopathies and Their Role in Early Life Epilepsies and Neurodevelopmental Disorders. Epilepsia Open *2*, 284–306. https://doi.org/10.1002/epi4.12062.

Kim, D.W., Place, E., Chinnaiya, K., Manning, E., Sun, C., Dai, W., Groves, I., Ohyama, K., Burbridge, S., Placzek, M., and Blackshaw, S. (2022). Single-cell analysis of early chick hypothalamic development reveals that hypothalamic cells are induced from prethalamic-like progenitors. Cell Rep. *38*, 110251. https://doi.org/10.1016/j.celrep.2021.110251.

Krienen, F.M., Goldman, M., Zhang, Q., C H Del Rosario, R., Florio, M., Machold, R., Saunders, A., Levandowski, K., Zaniewski, H., Schuman, B., et al. (2020). Innovations present in the primate interneuron repertoire. Nature *586*, 262–269. https://doi.org/10.1038/s41586-020-2781-z.

Langfelder, P., and Horvath, S. (2008). WGCNA: an R package for weighted correlation network analysis. BMC Bioinf. *9*, 559. https://doi.org/10.1186/1471-2105-9-559.

Langmead, B., and Salzberg, S.L. (2012). Fast gapped-read alignment with Bowtie 2. Nat. Methods *9*, 357–359. https://doi.org/10.1038/nmeth.1923.

Laub, F., Aldabe, R., Friedrich, V., Jr., Ohnishi, S., Yoshida, T., and Ramirez, F. (2001). Developmental expression of mouse Kruppel-like transcription factor KLF7 suggests a potential role in neurogenesis. Dev. Biol. *233*, 305–318. https://doi.org/10.1006/dbio.2001.0243.

Lee, B.R., Dalley, R., Miller, J.A., Chartrand, T., Close, J., Mann, R., Mukora, A., Ng, L., Alfiler, L., Baker, K., et al. (2023). Signature morphoelectric properties of diverse GABAergic interneurons in the human neocortex. Science *382*, eadf6484. https://doi.org/10.1126/science.adf6484.

Li, Y., Li, Z., Wang, C., Yang, M., He, Z., Wang, F., Zhang, Y., Li, R., Gong, Y., Wang, B., et al. (2023). Spatiotemporal transcriptome atlas reveals the regional specification of the developing human brain. Cell *186*, 5892–5909.e22. https://doi.org/10.1016/j.cell.2023.11.016.

Liao, Y., Smyth, G.K., and Shi, W. (2014). featureCounts: an efficient general purpose program for assigning sequence reads to genomic features. Bioinformatics *30*, 923–930. https://doi.org/10.1093/bioinformatics/btt656.

Lim, L., Mi, D., Llorca, A., and Marín, O. (2018). Development and Functional Diversification of Cortical Interneurons. Neuron *100*, 294–313. https://doi.org/10.1016/j.neuron.2018.10.009.

Limone, F., Guerra San Juan, I., Mitchell, J.M., Smith, J.L.M., Raghunathan, K., Meyer, D., Ghosh, S.D., Couto, A., Klim, J.R., Joseph, B.J., et al. (2023). Efficient generation of lower induced motor neurons by coupling Ngn2 expression with developmental cues. Cell Rep. *42*, 111896. https://doi.org/10.1016/j.celrep.2022.111896.

Liu, Z., Zhang, Z., Lindtner, S., Li, Z., Xu, Z., Wei, S., Liang, Q., Wen, Y., Tao, G., You, Y., et al. (2019). Sp9 Regulates Medial Ganglionic Eminence-Derived Cortical Interneuron Development. Cereb. Cortex *29*, 2653–2667. https://doi.org/10.1093/cercor/bhy133.

Longo, P.A., Kavran, J.M., Kim, M.S., and Leahy, D.J. (2013). Transient mammalian cell transfection with polyethylenimine (PEI). Methods Enzymol. *529*, 227–240. https://doi.org/10.1016/B978-0-12-418687-3.00018-5.

Ma, S., Zhang, B., LaFave, L.M., Earl, A.S., Chiang, Z., Hu, Y., Ding, J., Brack, A., Kartha, V.K., Tay, T., et al. (2020). Chromatin Potential Identified by Shared Single-Cell Profiling of RNA and Chromatin. Cell *183*, 1103–1116.e20. https://doi.org/10.1016/j.cell.2020.09.056.

Marín, O. (2012). Interneuron dysfunction in psychiatric disorders. Nat. Rev. Neurosci. *13*, 107–120. https://doi.org/10.1038/nrn3155.

Maroof, A.M., Keros, S., Tyson, J.A., Ying, S.W., Ganat, Y.M., Merkle, F.T., Liu, B., Goulburn, A., Stanley, E.G., Elefanty, A.G., et al. (2013). Directed differentiation and functional maturation of cortical interneurons from human embryonic stem cells. Cell Stem Cell *12*, 559–572. https://doi.org/10.1016/j.stem.2013.04.008.

Marro, S.G., Chanda, S., Yang, N., Janas, J.A., Valperga, G., Trotter, J., Zhou, B., Merrill, S., Yousif, I., Shelby, H., et al. (2019). Neuroligin-4 Regulates Excitatory Synaptic Transmission in Human Neurons. Neuron *103*, 617–626.e6. https://doi.org/10.1016/j.neuron.2019.05.043.

Mayer, C., Hafemeister, C., Bandler, R.C., Machold, R., Batista Brito, R., Jaglin, X., Allaway, K., Butler, A., Fishell, G., and Satija, R. (2018). Developmental diversification of cortical inhibitory interneurons. Nature *555*, 457–462. https://doi.org/10.1038/nature25999.

McGinnis, C.S., Murrow, L.M., and Gartner, Z.J. (2019). DoubletFinder: Doublet Detection in Single-Cell RNA Sequencing Data Using Artificial Nearest Neighbors. Cell Syst. *8*, 329–337.e4. https://doi.org/10.1016/j.cels.2019.03.003.

Miyoshi, G., Hjerling-Leffler, J., Karayannis, T., Sousa, V.H., Butt, S.J.B., Battiste, J., Johnson, J.E., Machold, R.P., and Fishell, G. (2010). Genetic fate mapping reveals that the caudal ganglionic eminence produces a large and diverse population of superficial cortical interneurons. J. Neurosci. *30*, 1582–1594. https://doi.org/10.1523/JNEUROSCI.4515-09.2010.

Moore, A.R., Filipovic, R., Mo, Z., Rasband, M.N., Zecevic, N., and Antic, S.D. (2009a). Electrical excitability of early neurons in the human cerebral cortex during the second trimester of gestation. Cereb. Cortex *19*, 1795–1805. https://doi.org/10.1093/cercor/bhn206.

Moore, D.L., Blackmore, M.G., Hu, Y., Kaestner, K.H., Bixby, J.L., Lemmon, V.P., and Goldberg, J.L. (2009b). KLF family members regulate intrinsic axon regeneration ability. Science *326*, 298–301. https://doi.org/10.1126/science.1175737.

Mullins, N., Forstner, A.J., O'Connell, K.S., Coombes, B., Coleman, J.R.I., Qiao, Z., Als, T.D., Bigdeli, T.B., Børte, S., Bryois, J., et al. (2021). Genome-wide association study of more than 40,000 bipolar disorder cases provides new insights into the underlying biology. Nat. Genet. *53*, 817–829. https://doi.org/10.1038/s41588-021-00857-4.

Nehme, R., Zuccaro, E., Ghosh, S.D., Li, C., Sherwood, J.L., Pietilainen, O., Barrett, L.E., Limone, F., Worringer, K.A., Kommineni, S., et al. (2018). Combining NGN2 Programming with Developmental Patterning Generates Human Excitatory Neurons with NMDAR-Mediated Synaptic Transmission. Cell Rep. *23*, 2509–2523. https://doi.org/10.1016/j.celrep.2018.04.066.

Nicholas, C.R., Chen, J., Tang, Y., Southwell, D.G., Chalmers, N., Vogt, D., Arnold, C.M., Chen, Y.J.J., Stanley, E.G., Elefanty, A.G., et al. (2013). Functional maturation of hPSC-derived forebrain interneurons requires an extended timeline and mimics human neural development. Cell Stem Cell *12*, 573–586. https://doi.org/10.1016/j.stem.2013.04.005.

Ostapcuk, V., Mohn, F., Carl, S.H., Basters, A., Hess, D., Iesmantavicius, V., Lampersberger, L., Flemr, M., Pandey, A., Thomä, N.H., et al. (2018). Activity-dependent neuroprotective protein recruits HP1 and CHD4 to control lineage-specifying genes. Nature *557*, 739–743. https://doi.org/10.1038/s41586-018-0153-8.

Pang, Z.P., Yang, N., Vierbuchen, T., Ostermeier, A., Fuentes, D.R., Yang, T.Q., Citri, A., Sebastiano, V., Marro, S., Südhof, T.C., and Wernig, M. (2011). Induction of human neuronal cells by defined transcription factors. Nature *476*, 220–223. https://doi.org/10.1038/nature10202.

Pantazis, C.B., Yang, A., Lara, E., McDonough, J.A., Blauwendraat, C., Peng, L., Oguro, H., Kanaujiya, J., Zou, J., Sebesta, D., et al. (2022). A reference human induced pluripotent stem cell line for large-scale collaborative studies. Cell Stem Cell *29*, 1685–1702.e22. https://doi.org/10.1016/j.stem.2022.11.004.

Pesold, C., Impagnatiello, F., Pisu, M.G., Uzunov, D.P., Costa, E., Guidotti, A., and Caruncho, H.J. (1998). Reelin is preferentially expressed in neurons synthesizing gamma-aminobutyric acid in cortex and hippocampus of adult rats. Proc. Natl. Acad. Sci. USA *95*, 3221–3226. https://doi.org/10.1073/pnas.95.6.3221.

Piñero, J., Ramírez-Anguita, J.M., Saüch-Pitarch, J., Ronzano, F., Centeno, E., Sanz, F., and Furlong, L.I. (2020). The DisGeNET knowledge platform for disease genomics: 2019 update. Nucleic Acids Res. *48*, D845–D855. https://doi.org/10.1093/nar/gkz1021.

Qin, J., Wang, C., Zhu, Y., Su, T., Dong, L., Huang, Y., and Hao, K. (2021). Mga safeguards embryonic stem cells from acquiring extraembryonic endoderm fates. Sci. Adv. *7*, eabe5689. https://doi.org/10.1126/sciadv.abe5689.

Rodriguez-Zas, S.L., Southey, N.L., Rund, L., Antonson, A.M., Nowak, R.A., and Johnson, R.W. (2023). Prenatal and postnatal challenges affect the hypothalamic molecular pathways that regulate hormonal levels. PLoS One *18*, e0292952. https://doi.org/10.1371/journal.pone.0292952.

RStudio Team (2020). RStudio (Integrated Development for R (RStudio, PBC)).

Sanchez-Priego, C., Hu, R., Boshans, L.L., Lalli, M., Janas, J.A., Williams, S.E., Dong, Z., and Yang, N. (2022). Mapping cis-regulatory elements in human neurons links psychiatric disease heritability and activity-regulated transcriptional programs. Cell Rep. *39*, 110877. https://doi.org/10.1016/j.celrep.2022.110877.

Schep, A.N., Wu, B., Buenrostro, J.D., and Greenleaf, W.J. (2017). chromVAR: inferring transcription-factor-associated accessibility from single-cell epigenomic data. Nat. Methods *14*, 975–978. https://doi.org/10.1038/nmeth.4401.

Schmitz, M.T., Sandoval, K., Chen, C.P., Mostajo-Radji, M.A., Seeley, W.W., Nowakowski, T.J., Ye, C.J., Paredes, M.F., and Pollen, A.A. (2022). The development and evolution of inhibitory neurons in primate cerebrum. Nature *603*, 871–877. https://doi.org/10.1038/s41586-022-04510-w.

Schulte, D., and Geerts, D. (2019). MEIS transcription factors in development and disease. Development *146*, dev174706. https://doi.org/10.1242/dev.174706.

Shi, Y., Wang, M., Mi, D., Lu, T., Wang, B., Dong, H., Zhong, S., Chen, Y., Sun, L., Zhou, X., et al. (2021). Mouse and human share conserved transcriptional programs for interneuron development. Science *374*, eabj6641. https://doi.org/10.1126/science.abj6641.

Singh, A., Mahesh, A., Noack, F., Cardoso de Toledo, B., Calegari, F., and Tiwari, V.K. (2022). Tcf12 and NeuroD1 cooperatively drive neuronal migration during cortical development. Development *149*, dev200250. https://doi.org/10.1242/dev.200250.

Skene, N.G., Bryois, J., Bakken, T.E., Breen, G., Crowley, J.J., Gaspar, H.A., Giusti-Rodriguez, P., Hodge, R.D., Miller, J.A., Muñoz-Manchado, A.B., et al. (2018). Genetic identification of brain cell types underlying schizophrenia. Nat. Genet. *50*, 825–833. https://doi.org/10.1038/s41588-018-0129-5.

Street, K., Risso, D., Fletcher, R.B., Das, D., Ngai, J., Yosef, N., Purdom, E., and Dudoit, S. (2018). Slingshot: cell lineage and pseudotime inference for single-cell transcriptomics. BMC Genom. *19*, 477. https://doi.org/10.1186/s12864-018-4772-0.

Supek, F., Bošnjak, M., Škunca, N., and Šmuc, T. (2011). REVIGO summarizes and visualizes long lists of gene ontology terms. PLoS One *6*, e21800. https://doi.org/10.1371/journal.pone.0021800.

Traynelis, S.F., Wollmuth, L.P., McBain, C.J., Menniti, F.S., Vance, K.M., Ogden, K.K., Hansen, K.B., Yuan, H., Myers, S.J., and Dingledine, R. (2010). Glutamate receptor ion channels: structure, regulation, and function. Pharmacol. Rev. *62*, 405–496. https://doi.org/10.1124/pr.109.002451.

Van de Sande, B., Flerin, C., Davie, K., De Waegeneer, M., Hulselmans, G., Aibar, S., Seurinck, R., Saelens, W., Cannoodt, R., Rouchon, Q., et al. (2020). A scalable SCENIC workflow for single-cell gene regulatory network analysis. Nat. Protoc. *15*, 2247–2276. https://doi.org/10.1038/s41596-020-0336-2.

Velmeshev, D., Schirmer, L., Jung, D., Haeussler, M., Perez, Y., Mayer, S., Bhaduri, A., Goyal, N., Rowitch, D.H., and Kriegstein, A.R. (2019). Single-cell genomics identifies cell type-specific molecular changes in autism. Science *364*, 685–689. https://doi.org/10.1126/science.aav8130.

Wamsley, B., and Fishell, G. (2017). Genetic and activity-dependent mechanisms underlying interneuron diversity. Nat. Rev. Neurosci. *18*, 299–309. https://doi.org/10.1038/nrn.2017.30.

Wang, Y., Dye, C.A., Sohal, V., Long, J.E., Estrada, R.C., Roztocil, T., Lufkin, T., Deisseroth, K., Baraban, S.C., and Rubenstein, J.L.R. (2010). Dlx5 and Dlx6 regulate the development of parvalbumin-expressing cortical interneurons. J. Neurosci. *30*, 5334–5345. https://doi.org/10.1523/JNEUROSCI.5963-09.2010.

Wickham, H. (2016). ggplot2: Elegant Graphics for Data Analysis (Springer-Verlag).

Yamakawa, H., Cheng, J., Penney, J., Gao, F., Rueda, R., Wang, J., Yamakawa, S., Kritskiy, O., Gjoneska, E., and Tsai, L.H. (2017). The Transcription Factor Sp3 Cooperates with HDAC2 to Regulate Synaptic Function and Plasticity in Neurons. Cell Rep. *20*, 1319–1334. https://doi.org/10.1016/j.celrep.2017.07.044.

Yang, N., Chanda, S., Marro, S., Ng, Y.H., Janas, J.A., Haag, D., Ang, C.E., Tang, Y., Flores, Q., Mall, M., et al. (2017). Generation of pure GABAergic neurons by transcription factor programming. Nat. Methods *14*, 621–628. https://doi.org/10.1038/nmeth.4291.

Yu, G., Wang, L.G., Han, Y., and He, Q.Y. (2012). clusterProfiler: an R package for comparing biological themes among gene clusters. OMICS *16*, 284–287. https://doi.org/10.1089/omi.2011.0118.

Yu, Y., Zeng, Z., Xie, D., Chen, R., Sha, Y., Huang, S., Cai, W., Chen, W., Li, W., Ke, R., and Sun, T. (2021). Interneuron origin and molecular diversity in the human fetal brain. Nat. Neurosci. *24*, 1745–1756. https://doi.org/10.1038/s41593-021-00940-3.

Zhang, B., and Horvath, S. (2005). A general framework for weighted gene co-expression network analysis. Stat. Appl. Genet. Mol. Biol. *4*, Article17. https://doi.org/10.2202/1544-6115.1128.

Zhang, Y., Liu, T., Meyer, C.A., Eeckhoute, J., Johnson, D.S., Bernstein, B.E., Nusbaum, C., Myers, R.M., Brown, M., Li, W., and Liu, X.S. (2008). Model-based analysis of ChIP-Seq (MACS). Genome Biol. *9*, R137. https://doi.org/10.1186/gb-2008-9-9-r137.

Zhang, Y., Pak, C., Han, Y., Ahlenius, H., Zhang, Z., Chanda, S., Marro, S., Patzke, C., Acuna, C., Covy, J., et al. (2013). Rapid single-step induction of functional neurons from human pluripotent stem cells. Neuron *78*, 785–798. https://doi.org/10.1016/j.neuron.2013.05.029.

Zhang, Y., Sloan, S.A., Clarke, L.E., Caneda, C., Plaza, C.A., Blumenthal, P.D., Vogel, H., Steinberg, G.K., Edwards, M.S.B., Li, G., et al. (2016). Purification and Characterization of Progenitor and Mature Human Astrocytes Reveals Transcriptional and Functional Differences with Mouse. Neuron *89*, 37–53. https://doi.org/10.1016/j.neuron.2015.11.013.

Zhou, W., Gross, K.M., and Kuperwasser, C. (2019). Molecular regulation of Snai2 in development and disease. J. Cell Sci. *132*, jcs235127. https://doi.org/10.1242/jcs.235127.

STAR★METHODS

KEY RESOURCES TABLE

REAGENT or RESOURCE	SOURCE	IDENTIFIER
**Antibodies**		
Anti-ADNP	Bethyl Laboratories, Inc	Cat#A300-104A; RRID: AB_242522
Anti-HA	Roche	Cat#11867423001; RRID: AB_390918
Anti-CTCF	Cell Signaling Technology	Cat#3418S; RRID: AB_2086791
Anti-HP1γ	Cell Signaling Technology	Cat#2619S; RRID: AB_2070984
Anti-CHD4	Bethyl Laboratories, Inc	Cat#A301-081A; RRID: AB_873001
Anti-MAP2	Abcam	Cat#ab5392; RRID: AB_2138153
Anti-Synapsin	Synaptic Systems	Cat#106 002; RRID: AB_887804
Anti-FOXG1	Takara Bio	Cat#M227; RRID: AB_2827749
Anti-Sox2	R&D Systems, Inc.	Cat#AF2018; RRID: AB_355110
Anti-Pax6	BioLegend	Cat#901301; RRID: AB_2565003
Anti-H3K27ac	Abcam	Cat#AB4729; RRID: AB_2118291
Anti-IgG	Abcam	Cat#AB46540; RRID: AB_2614925
Anti-IgG	Novus Biologicals, LLC	Cat#NBP1-72763; RRID:AB_11024108
Anti-FOS	Abcam	Cat#AB208942; RRID: AB_2747772
Anti-GAPDH	Cell Signaling Technology	Cat#2118; RRID: AB_561053
Anti-β-actin	Sigma-Aldrich	Cat#A5441; RRID: AB_476744
Anti-Dlx5	John L.R. Rubenstein; Wang et al. (2010)	N/A
Anti-Dlx2	Santa Cruz Biotechnology, Inc	Cat#sc-393879; RRID:AB_3678657
Anti-GAD67	Millipore	Cat#MAB5406; RRID: AB_2278725
Anti-CALB2	Agilent	Cat#M7245; RRID: AB_2068519
Anti-Calbindin	Swant	Cat#300; RRID: AB_10000347
Anti-Pax6	Covance	Cat#PRB-278P; RRID: AB_291612
Anti-Somatostatin	Novus	Cat#NBP2-37447; RRID: AB_2890053
Anti-Human Nuclear Antigen	Millipore	Cat#MAB1281; RRID: AB_94090
Anti-SP8	Santa Cruz Biotechnology	Cat#sc-104661; RRID: AB_2194626
Anti-Synaptophysin	Thermo Fisher Scientific	Cat#14-6525-82; RRID: AB_10670280
Anti-NeuN	Millipore	Cat#MAB377; RRID: AB_2298772
Peroxidase-AffiniPure Donkey Anti-Rabbit IgG	Jackson ImmunoResearch Labs	Cat#711-035-152; RRID: AB_10015282
IRDye 680RD Donkey anti-Mouse IgG	LI-COR Biosciences	Cat#926–68072; RRID: AB_10953628
IRDye 800CW Goat anti-Rat IgG	LI-COR Biosciences	Cat#926–32219; RRID: AB_1850025

(*Continued on next page*)

Continued		
**REAGENT or RESOURCE**	**SOURCE**	**IDENTIFIER**
Donkey anti-Mouse IgG 488	Thermo Fisher Scientific	Cat#A-21202; RRID: AB_141607
Donkey anti-Mouse IgG 555	Thermo Fisher Scientific	Cat#A-31570; RRID: AB_2536180
Donkey anti-Rabbit IgG 555	Thermo Fisher Scientific	Cat#A-31572; RRID: AB_162543
Donkey anti-Rabbit IgG 488	Thermo Fisher Scientific	Cat#A-21206; RRID: AB_2535792
Donkey anti-Chicken IgG 647	Thermo Fisher Scientific	Cat#A78952; RRID: AB_2921074
**Chemicals, peptides, and recombinant proteins**		
Stemflex supplement	Gibco; Thermo Fisher Scientific	Cat#A33492
Stemflex basal medium	Gibco; Thermo Fisher Scientific	Cat#A33493-01
MEM medium	Gibco; Thermo Fisher Scientific	Cat#11090-081
Neurobasal medium	Gibco; Thermo Fisher Scientific	Cat#21103-049
Neurobasal A medium	Gibco; Thermo Fisher Scientific	Cat#10888-022
DMEM/F12 medium	Gibco; Thermo Fisher Scientific	Cat#11320-033
DMEM medium	Gibco; Thermo Fisher Scientific	Cat#11965-092
Knockout DMEM	Gibco; Thermo Fisher Scientific	Cat#10829018
B27 supplement	Gibco; Thermo Fisher Scientific	Cat#17504-044
N2 supplement	Gibco; Thermo Fisher Scientific	Cat#17502-048
KnockOut™ Serum Replacement	Gibco; Thermo Fisher Scientific	Cat#10828028
MEM NEAA (100×)	Gibco; Thermo Fisher Scientific	Cat#11140-050
Glutamax (100×)	Gibco; Thermo Fisher Scientific	Cat#35050-061
Sodium Pyruvate (100×)	Gibco; Thermo Fisher Scientific	Cat#11360-070
β-Mercaptoethanol	Sigma-Aldrich	Cat#M3148
Cosmic Calf™ Serum	Cytiva	Cat#SH3008704
Fetal Bovine Serum	Sigma-Aldrich	Cat#F6178
D-PBS tablets	Millipore	Cat#524650-1EA
GelTrex	Gibco; Thermo Fisher Scientific	Cat#A14133-02
Poly-L-ornithine hydrobromide	Sigma-Aldrich	Cat#P3655
Accutase	Innovative Cell Technologies	Cat#AT104500
Trypsin 0.25%	Gibco; Thermo Fisher Scientific	Cat#25200-114
Puromycin dihydrochloride	Sigma-Aldrich	Cat#P8833
Hygromycin B (50 mg/mL)	Gibco; Thermo Fisher Scientific	Cat#10687010
Cytosine β-D-arabinofuranoside	Sigma-Aldrich	Cat#C1768
Doxycycline (DOX)	Sigma-Aldrich	Cat#D9891
Bovine Serum Albumin	Equitech-Bio Inc	Cat#BAH65-0500
Thiazovivin	Santa Cruz Biotechnology, Inc	Cat#SC-361380
Y-27632	Tocris Bioscience	Cat#1254

(*Continued on next page*)

Continued

REAGENT or RESOURCE	SOURCE	IDENTIFIER
Chroman1	MedChemExpress LLC	Cat#HY-15392
LDN193189	Stemgent	Cat#04-0074
SB431542	Tocris Bioscience	Cat#1614
XAV939	Stemgent	Cat#04-0046
DAPT	Tocris Bioscience	Cat#2634
SHH	R&D Systems	Cat#1845-SH
Purmorphamine	R&D Systems	Cat#SML0868
Dimethyl sulfoxide	Sigma-Aldrich	Cat#D2438
Paraformaldehyde solution	Affymetrix	Cat#19943
Halt Protease and Phosphatase Inhibitor Cocktail	Thermo Fisher Scientific	Cat#78440
**Critical commercial assays**		
Pierce RIPA Buffer	Thermo Fisher Scientific	Cat#89900
Bolt™ 4 to 12%, Bis-Tris, Protein Gel	Thermo Fisher Scientific	Cat#MW04125BOX
iBlot™ 2 Transfer Stacks, PVDF	Thermo Fisher Scientific	Cat#IB24001
Western Lightning Plus, Chemiluminescent Substrate	Revvity Health Sciences Inc	Cat#NEL104001EA
Pierce™ BCA Protein Assay Kits	Thermo Fisher Scientific	Cat#23225
Intercept® (PBS) Blocking Buffer	LI-COR Biosciences	Cat#927-70001
Chromium Next GEM Single Cell 3′ GEM Kit v3.1	10× Genomics	Cat#PN-1000123
Chromium Next GEM Single Cell 3′ Gel Bead Kit v3.1	10× Genomics	Cat#PN-1000122
Chromium Next GEM Chip G Single Cell Kit	10× Genomics	Cat#PN-1000120
**Deposited data**		
Raw single-cell sequencing data and normalized counts	This paper	GEO: GSE277602; GEO: GSE277604
**Experimental models: Cell lines**		
Human iPSC line: PGP1	Coriell	Cat#GM23338; RRID: CVCL_F182
Human iPSC line: WTC11	Coriell	Cat#GM25256; RRID: CVCL_Y803
Human iPSC line: CC1599.101A (female control)	This paper	N/A
Human ESC line: HES-3 NKX2.1GFP/w	Goulburn et al. (2011)	RRID: CVCL_A5HB
Human cell line: HEK293T	ATCC	Cat#11268; RRID: CVCL_1926
**Experimental models: Organisms/strains**		
Mouse: CD1	Charles River	Cat#CRL:022; RRID: IMSR_CRL:022

(*Continued on next page*)

Continued

REAGENT or RESOURCE	SOURCE	IDENTIFIER
**Recombinant DNA**		
FUW-M2rtTA	Hockemeyer et al. (2008)	Addgene plasmid #20342; RRID: Addgene_20342
TetO-Ascl1-puro	Yang et al. (2017)	Addgene plasmid #97329; RRID: Addgene_97329
TetO-Dlx2-Hygro	Yang et al. (2017)	Addgene plasmid #97330; RRID: Addgene_97330
pMD2.G	Addgene	Addgene plasmid #12259; RRID: Addgene_12259
pRSV-rev	Addgene	Addgene plasmid #12253; RRID: Addgene_12253
pMDLg/pRRE	Addgene	Addgene plasmid #12251; RRID: Addgene_12251
PB-TO-ASCL1-DLX2	Addgene	Addgene plasmid #182307; RRID: Addgene_182307
**Software and algorithms**		
Bowtie 2	Langmead and Salzberg (2012)	RRID: SCR_016368; https://bowtie-bio.sourceforge.net/bowtie2/
Cell Ranger v7.2.0	10× Genomics	RRID: SCR_023221; https://www.10xgenomics.com/support/software/cell-ranger
chromVAR	Schep et al. (2017)	RRID: SCR_026570; https://github.com/GreenleafLab/chromVAR
clusterProfiler v4.8.3	Yu et al. (2012)	RRID: SCR_016884; https://bioconductor.org/packages/clusterProfiler/
DoubletFinder v2.0.4	McGinnis et al. (2019)	RRID: SCR_018771; https://github.com/chris-mcginnis-ucsf/DoubletFinder
Enrichr	Chen et al. (2013)	RRID: SCR_001575; https://maayanlab.cloud/Enrichr/
featureCounts (Subread)	Liao et al. (2014)	RRID: SCR_012919; https://subread.sourceforge.net/
Gephi v0.10.1	Gephi	RRID: SCR_004293; https://gephi.org/
ggplot2 v3.4.3	Wickham (2016)	RRID: SCR_014601; https://ggplot2.tidyverse.org/
MACS2	Zhang et al. (2008)	RRID: SCR_013291; https://github.com/macs3-project/MACS
MetaCell	Baran et al. (2019)	RRID: SCR_017013; https://github.com/tanaylab/metacell
Monocle3 v1.3.7	Cao et al. (2019)	RRID: SCR_018685; https://cole-trapnell-lab.github.io/monocle3/
Nextflow	Di Tommaso et al. (2017)	RRID: SCR_024135; https://www.nextflow.io/

(*Continued on next page*)

Continued

REAGENT or RESOURCE	SOURCE	IDENTIFIER
ZEISS ZEN Blue v3.7.4	ZEISS	RRID: SCR_013672; https://www.zeiss.com/microscopy/en/products/software/zeiss-zen.html
pySCENIC v0.12.1	Van de Sande et al. (2020)	RRID: SCR_025802; https://github.com/aertslab/pySCENIC
R v4.3.1	R Core Team	https://www.r-project.org/
REVIGO v1.8.1	Supek et al. (2011)	RRID: SCR_005825; https://revigo.irb.hr/
RStudio v1.3.1093	RStudio Team (2020)	RRID: SCR_000432; https://posit.co/downloads/
SCENIC v1.1.2	Aibar et al. (2017)	RRID: SCR_017247; https://github.com/aertslab/SCENIC
SCopeLoomR v0.13.0	Aerts laboratory	N/A; https://github.com/aertslab/SCopeLoomR
Seurat v4.0.1	Butler et al. (2018)	RRID: SCR_016341; https://satijalab.org/seurat/
SingleCellExperiment v1.22.0	Amezquita et al. (2020)	https://bioconductor.org/packages/SingleCellExperiment/
Slingshot v2.8.0	Street et al. (2018)	RRID: SCR_017012; https://bioconductor.org/packages/slingshot/
STAR	Dobin et al. (2013)	RRID: SCR_004463; https://github.com/alexdobin/STAR
ToppGene Suite	Chen et al. (2009)	RRID: SCR_005726; https://toppgene.cchmc.org/
WGCNA	Langfelder and Horvath (2008)	RRID: SCR_003302; https://cran.r-project.org/package=WGCNA
**Other**		
Bio-Rad ChemiDoc MP Imaging System	Bio-Rad	RRID: SCR_019037
Chromium iX	10× Genomics	Cat#PN-1000328; RRID: SCR_024536
ZEISS LSM 980 with Airyscan 2 Microscope	ZEISS	RRID: SCR_025048
EVOS M5000 Imaging System	Thermo Fisher Scientific	Cat#AMF5000; RRID: SCR_023650

EXPERIMENTAL MODEL AND STUDY PARTICIPANT DETAILS

Animal model

All animal experiments were approved by the Institutional Animal Care and Use Committee and were conducted in compliance with the relevant ethical regulations. Mice were maintained in social cages on a 12-h light/dark cycle with free access to food and water; animals were monitored daily for food and water intake. Wild-type CD1 mice were used to isolate primary cell cultures on postnatal day 3 (P03). Rag2$^{-/-}$ mice were used as neonatal recipients for transplantation experiments. Animals of both sexes were used in the analyses.

Human PSC lines and cell culture

Human PSCs were maintained on Geltrex-coated plates in Stemflex medium and a 5% CO2 environment at 37°C. Cultures were visually inspected during routine medium changes for evidence of bacterial or fungal contamination to ensure that only contamination-free cultures were used for experiments. Cells were passaged using Accutase in Stemflex

supplemented with Chroman1 (2 μM), which was removed from the medium the following day. Cells were cryopreserved in BAMBANKER cryopreservation medium and recovered in StemFlex medium supplemented with Chroman1. PSC lines used for differentiation were maintained between passages 35 and 45. Pluripotency was routinely assessed by flow cytometry for SSEA-4 and TRA-1-60, with >95% of cells positive for these markers prior to differentiation. Cultures were tested monthly for mycoplasma contamination by PCR and were negative prior to experimentation.

To generate genetically modified PGP1 and WTC11 iPSCs with the *ADNP* p.Tyr719* variant, we altered the nucleotide sequence NM_015339.4:c.2157C>G. Additionally, we inserted hemagglutinin (HA) tags immediately after Tyr719 to detect the resulting truncated proteins. In parallel, as a control, we inserted HA tags at the 3′ end of the endogenous ADNP gene in the PGP1 line. sgRNAs targeting proximal regions were designed using the CHOPCHOP online tool (https://chopchop.cbu.uib.no/) and cloned into the lentiCRISPRv1 plasmid (Addgene Plasmid #49535) using the Golden Gate Assembly protocol. The gene-targeting template plasmid used to generate the HA-tagged *ADNP* p.Tyr719* allele includes a puromycin-resistance cassette flanked by two flippase recognition target (FRT) sites for FlipE recombinase mediated deletion, positioned after the HA tag sequence, and the 5′ and 3′ DNA homology arms. A similar gene-targeting template was used to generate the HA-tagged *ADNP* allele. The day before transfection, human iPSCs were dissociated into single cells with Accutase and plated at 250,000 cells per well in six-well plates containing StemFlex medium with Chroman1. For transfection, the template vector (1 μg) and the CRISPRv1-gRNA (0.5 μg) were incubated with Lipofectamine Stem Transfection Reagent (10 μL) and OptiMEM (250 μL, Invitrogen) for 10 min, then added dropwise to the iPSCs. Selection with puromycin (1 μg/mL) in StemFlex medium was initiated 24–48 h after transfection and continued for approximately 10 days until stable colonies formed. Individual colonies were picked with a P200 pipette tip under a microscope. Heterozygous and homozygous ADNP p.Tyr719-HA* iPSC clones, as well as heterozygous ADNP-HA iPSC clones, were confirmed by PCR. The puromycin-resistance cassette was removed using FlipE recombinase.

To generate iPSCs with stable inducible ASCL1-DLX2 integration, we took advantage of a bi-directional tetracycline-inducible promoter via the PiggyBac transposon system, as previously described (Pantazis et al., 2022). The iPSCs were transfected with the PB-TO-ASCL1-DLX2 plasmid and the EF1a-transposase vector at a 2:1 ratio using Lipofectamine™ Stem Transfection Reagent. Selection with puromycin started 24–48 h post-transfection and continued for 2 weeks. Successfully modified human iPSCs harboring stable TO-ASCL1-DLX2 integration were either cryopreserved for future applications or directly differentiated into neuronal cells. PB-TO-ASCL1-DLX2 (Addgene plasmid #182307) and the EF1a-transposase plasmid were gifts from the iPSC Neurodegenerative Disease Initiative (iNDI) & Michael Ward.

Research using human-derived samples was conducted in accordance with protocols approved by the institutional review boards of the Icahn School of Medicine at Mount Sinai.

Lentivirus production

Infectious lentiviral particles were produced in HEK293T cells using the third-generation lentiviral packaging plasmids: pMD2.G (Addgene plasmid # 12259), pRSV-rev (Addgene plasmid #12253), and pMDLg/pRRE (Addgene plasmid #12251) (Dull et al., 1998). Lentiviruses were produced in HEK293T cells using polyethylenimine (PEI) according to established protocols (Longo et al., 2013). The lentiviral supernatant was centrifuged at 1,000g for 5 min to remove cellular debris. Lentiviral particles were then ultra-centrifuged, resuspended overnight with gentle shaking in DMEM, aliquoted, and stored at −80°C. Only virus preparations with >90% infection efficiency were used for experiments, as determined by GFP expression or antibiotic resistance.

Small molecule-mediated GABAergic neuron generation

Human PSCs were differentiated into cortical inhibitory neurons using an established protocol based on dual SMAD inhibition, WNT inhibition, and SHH activation (Maroof et al., 2013; Nicholas et al., 2013) with the following modifications. NKX2.1(GFP/w) cells were dissociated into single cells using Accutase and plated at 430,000 cells/cm^2 onto Geltrex-coated wells in KSR medium (415mL Knockout DMEM, 75 mL knockout serum replacement, 500 μL 2-mercaptoethanol, 5 mL NEAA, and 5 mL GlutaMAX) supplemented with 10 μM Y-27632. From days 1–10, the cells underwent daily complete medium changes with LDN193189 (100 nM), SB431542 (10 μM), and XAV939 (2 μM). During days 11–18, N2 medium supplemented with 1 μM Purmorphamine and 5 μM recombinant SHH was used to induce ventral identity. On day 32, NKX2.1-GFP cells were dissociated, collected via FACS, and replated with mouse glial cells. The cells were maintained in Neurobasal A medium supplemented with 1×B27, 1×Glutamax, 1% fetal bovine serum, and 2-4 μM AraC and harvested on day 67 for further analyses.

TF-mediated induced GABAergic neuron generation

We used our established protocol to generate GABAergic neurons through lentiviral-mediated overexpression of ASCL1 and DLX2, as previously described (Yang et al., 2017). Briefly, PSCs were transduced with FUW-TetO-Ascl1-T2A-puromycin, FUW-TetO-Dlx2-IRES-hygromycin, and FUW-rtTA lentiviruses. Twenty-four hours later, the medium was replaced with N2 medium (1xN2 supplement, 1xNEAA in DMEM-F12) supplemented with 2 μg/mL doxycycline to induce transgene expression. The transduced cells were selected for three days by applying hygromycin (200 μg/mL) and puromycin (1 μg/mL). Subsequently, the medium was replaced with N2 medium containing 4 μM AraC for an additional three days. On day 8, GABAergic neurons were co-cultured with mouse glial cells at a density of 104,000 cells/cm^2 on Geltrex-coated plates. Two weeks post-transgene induction, doxycycline was withdrawn, and the culture was maintained in Neurobasal A medium supplemented with 1× B27, 1× Glutamax, and 1% fetal bovine serum. Mature neurons were used for various assays on day 35 after co-culture.

To generate GABAergic neurons from PSC lines with stable inducible ASCL1-DLX2 integration, PSCs were dissociated using Accutase and plated at a density of 100,000 cells/cm^2 on GelTrex-coated plates in StemFlex Medium supplemented with Chroman1. The following day, the medium was replaced with N2 medium containing 2 μg/mL doxycycline to induce ASCL1 and DLX2 expression. After three days, 4 μM AraC was added to the N2 medium. On day 8, the neurons were replated with mouse glial cells. All subsequent procedures followed the previously described protocol for generating GABAergic neurons using lentiviral vectors.

Patterned induced GABAergic neuron generation

PSC lines with stable inducible ASCL1-DLX2 integration were dissociated into single cells using Accutase and plated at 430,000 cells/cm^2 onto Geltrex-coated wells in KSR medium supplemented with 2 μM Chroman1. From days 1–10, the cells underwent daily full medium changes with LDN193189 (100 nM), SB431542 (10 μM), and XAV939 (2 μM). Starting on day 11, N2 medium supplemented with 2 μg/mL doxycycline and 10 μM DAPT (Tocris Bioscience, 2634) was used to induce ASCL1 and DLX2 expression and promote synchronized neural differentiation. Doxycycline was discontinued either 3 or 7 days later, and DAPT was removed after 7 days. Neurons were then dissociated and replated with mouse glial cells. Subsequent steps followed the established protocols used for other GABAergic neuron differentiation methods.

METHOD DETAILS

Primary mouse astrocytes

Mouse glial cultures were generated from cortical hemispheres at postnatal day 3 (P03). The cortices were incubated in 3 mL of 20 Units/mL Papain, 0.5 μM EDTA, and 1 μM $CaCl_2$ in HBSS for 15 min. After incubation, the tissues were manually dissociated by forceful trituration. The resulting cells were grown at 37°C with 5% CO_2 in DMEM medium containing 1×NEAA, 1× sodium pyruvate, 10% Cytiva HyClone™ Cosmic Calf™ Serum (CCS), and 0.008% β-mercaptoethanol.

Transplantation and brain slice preparation

Patterned-iNs were transplanted into neonatal Rag2$^{-/-}$ recipient mice at postnatal day 2 (P2). Concentrated cell suspensions ($\sim 1 \times 10^5$ cells/μL) were loaded into beveled glass micropipettes (Drummond Scientific). Each recipient received two injections of 0.5 μL into the forebrain at coordinates 2.0 mm posterior, 1.0 mm lateral, and 1.5 mm ventral to the skin surface, targeting the periventricular region. Stereotaxic coordinates were zeroed anteriorly at the inner corner of the eye, laterally at the midpoint between the eyes, and vertically at the skin surface. Following injection, pups were placed on a heating pad until fully warmed and active, then returned to their mothers. Animals were maintained with the dam until weaning at postnatal day 21 (P21).

Acute brain slices were prepared from approximately 5-month-old mice. Animals were deeply anesthetized with Euthasol and decapitated. Brains were rapidly removed and immersed in ∼0°C aerated (95% O_2 and 5% CO_2) sucrose-based slicing solution containing (in mM): 213 sucrose, 2.5 KCl, 2 $MgSO_4$, 2 $CaCl_2$, 26 $NaHCO_3$, 1.25 NaH_2PO_4, and 25 dextrose (315 mOsm, pH 7.4). Coronal slices 300 μm thick were cut in this solution using a vibratome (Leica VT1200S). Slices were cut from both female and male mouse brain and then incubated in aerated artificial cerebrospinal fluid (ACSF) containing (in mM): NaCl 126, KCl 2.5, NaH_2PO_4 1.25, $NaHCO_3$ 26, dextrose 25, $MgSO_4$ 2 and $CaCl_2$ 2 (315 mOsm, pH 7.4) at ∼34°C for 50 min and then maintained at room temperature until recording.

Electrophysiological recordings from brain slices

Slices were transferred to a recording chamber and perfused with oxygenated ACSF at 25°C. GFP-positive human neurons were identified in acute brain slices using an infrared differential interference contrast (IR-DIC) microscope (BX51WI, Olympus) equipped with a 40× water-immersion objective and were targeted for whole-cell recording. Images were captured with a CMOS camera (C11440-36U, Hamamatsu) and displayed in real time. GFP-expressing human neurons were identified using 470-nm LED excitation. To assess intrinsic membrane properties, whole-cell current-clamp recordings were performed using 500-ms current steps of increasing amplitude. Patch pipettes (~5 MΩ) were filled with a K-gluconate–based internal solution containing (in mM): 140 K-Gluconate, 3 KCl, 2 $MgCl_2$, 0.2 EGTA, 10 HEPES and 2 Na_2ATP (287 mOsm, pH 7.22), supplemented with 0.2% biocytin for post hoc morphological analysis. Voltage signals were filtered at 10 kHz and sampled at 40 kHz, while synaptic currents were low-pass filtered at 3 kHz and sampled at 40 kHz using a Multiclamp 700B amplifier and Clampex 11.2 software (Molecular Devices).

Whole-cell recordings from cultured neurons

Functional properties of iNs were assessed using whole-cell patch-clamp electrophysiology as previously described (Pang et al., 2011; Yang et al., 2017). Spontaneous inhibitory postsynaptic currents (sIPSCs) were recorded at a holding potential of −70 mV in the presence of 20 μM 6-cyano-7-nitroquinoxaline-2,3-dione (CNQX). Miniature inhibitory postsynaptic currents (mIPSCs) were recorded at −70 mV in the presence of 1 μM tetrodotoxin (TTX) and 20 μM CNQX. Patch pipettes were filled with a Cs-based internal solution containing (in mM): 40 CsCl, 3.5 KCl, 10 HEPES, 0.05 EGTA, 90 K-gluconate, 1.8 NaCl, 1.7 $MgCl_2$, 2 Mg-ATP, 0.4 Na-GTP, and 10 phosphocreatine. The external recording solution contained (in mM): 140 NaCl, 5 KCl, 10 HEPES, 2 $CaCl_2$, 2 $MgCl_2$, and 10 glucose (pH 7.4). All recordings were performed at room temperature.

Immunofluorescence and imaging

Cultured cells were fixed with 4% paraformaldehyde in D-PBS at room temperature for 5–10 min. Cells were rinsed 3 times with D-PBS and subsequently permeabilized for 5 min at room temperature with 0.2% Triton X-100 in D-PBS. After incubation in blocking buffer (4% bovine serum albumin (BSA) and 1% Cosmic Calf™ Serum in D-PBS) for 3 h, primary antibodies were added for incubation overnight at 4°C. After 3 rinses, cells were incubated with secondary antibodies in the blocking buffer for 3 h at room temperature. Images were acquired using the EVOS M5000 imaging system (Life Technologies). For marker quantification in Figure 2I, the proportion of $MAP2^+$ neurons co-expressing each marker was calculated by manual counting across two iPSC lines, PGP1 and WTC11, with three independent differentiations analyzed per line. For each differentiation, marker-positive cells were expressed as a percentage of $MAP2^+$ neurons, and each independent differentiation was treated as one biological replicate. Data are reported as mean ± SD across independent differentiations. Donor-associated differences were evaluated using two-sided Welch's t-tests. Inter-differentiation reproducibility was assessed descriptively from the distribution of the three independent differentiation-level measurements within each donor.

Images of injected human neurons in mouse brain sections were acquired at the Microscopy and Advanced Bioimaging CoRE at the Icahn School of Medicine at Mount Sinai. Imaging was performed using ZEN Blue software (v3.7.4) on a Zeiss LSM980 confocal microscope equipped with a 32-channel Airyscan 2 GaAsP-PMT detector, operated in super-resolution (SR) mode with Multiplex 4Y. Images were collected using a 100×/1.46 NA α-Plan-Apochromat oil-immersion objective with a minimum optical zoom of 1.7. Frame size was set to "optimal" to ensure Nyquist sampling for the selected field of view. Images were acquired at 16-bit depth, with scan speed set to "maximum" and a frame average of 1. Excitation was provided by an argon 488-nm laser for GFP and a 561-nm diode laser for Alexa Fluor 561. Emission was collected using a main beam splitter (MBS) 488/561, a secondary beam splitter (SBS) SP615, and dual emission filters (42 BP0–480 and 49 BP5–550 for GFP; 57 BP0–620 and LP645 for Alexa Fluor 561). For z stack acquisition, the z-step size was set to half of the axial resolution (0.4 μm per step) to ensure adequate sampling. For each experimental cohort, positive control samples were used to establish excitation and detection parameters, including laser power and detector gain. Settings were optimized to maximize signal-to-noise ratio while using approximately 50% of the detector's dynamic range and avoiding saturation. Detector gain was maintained below 850 V to ensure linear response, and laser power rarely exceeded 1%. Once established, these parameters were held constant for all samples within each cohort. Image processing was performed using the ZEN Airyscan processing module.

Immunoblotting

Cells were washed three times with cold PBS, harvested using CELLTREAT cell lifters, and lysed with the RIPA Lysis and Extraction Buffer (supplemented with 1× Halt Protease and Phosphatase Inhibitor Cocktail and 5 mM EDTA). Cells were incubated on ice for 15 min with occasional vortexing, sonicated twice (1 s pulse followed by 1 min on ice), incubated on ice for another 15 min with occasional vortexing, and then centrifuged at 21,000 × g for 20 min with the supernatant collected for quantifications using the BCA assay. Samples were denatured with 1×NuPAGE LDS loading buffer (supplemented with 50 mM DTT) and incubated at 70°C for 10 min.

Approximately 25 μg of total proteins were loaded onto each well of the 4–12% Bis-Tris gel and run at 170 V for 30 min in 1× MOPS Buffer. Samples were then transferred onto PVDF membrane at 20 V for 1 h at 4°C in cold 1×Transfer Buffer (25 mM Tris, 200 mM Glycine, 15% MeOH in ddH2O), blocked with either the LICOR Blocking Buffer or 5% milk in TBST for 1 h at RT, then incubated overnight at 4°C on the rocking shaker with the addition of primary antibodies diluted in the corresponding blocking buffer supplemented with 0.1% Tween 20. Membranes were washed four times with TBST (5 min at RT on the shaker each time), incubated at RT for 1 h with secondary antibodies (diluted in the corresponding blocking buffer and supplemented with 0.1% Tween 20 and 0.1% SDS), washed four additional times with TBST (for HRP antibodies, treated with the ECL buffer at A:B = 1:1 in dark for 5 min at RT before imaging), and imaged using the Bio-Rad ChemiDoc MP Imaging System.

10× single-cell RNA-seq library preparation and sequencing

We used a Papain solution to create single-cell suspensions based on a published method (Zhang et al., 2016). Neurons were detached from the plate using Accutase supplemented with 0.4% DNase (Worthington), collected and further dissociated with a Papain solution. This solution was prepared by adding 328 μL of Papain to each 20 mL Enzyme Stock Solution containing 1×EBSS, 0.46% D(+) Glucose, 26 mM $NaHCO_3$, and 0.5 mM EDTA. Additionally, 0.004 g of L-cysteine was added before use. The solution was activated for 15 min at 37°C, followed by an additional 1.5-h incubation at 37°C. Prepare the Inhibitor Stock Solution (0.46% D(+) Glucose and 25 mM $NaHCO_3$ in 1×EBSS), Low Ovo (10×) solution (3 g BSA (Sigma A8806), and 3 g Trypsin inhibitor (Worthington LS003086) in 200 mL D-PBS, adjusting the pH to 7.4) and High Ovo (5×) solution (6 g BSA and 6 g Trypsin inhibitor in 200 mL D-PBS, pH 7.4). The single-cell suspension was washed four times, alternating between low Ovo solution (9 mL inhibitor stock solution and 1 mL Low Ovo (10×)) and high Ovo solution (4 mL inhibitor stock solution and 1 mL High Ovo (5×)). After washing, the cells were centrifuged at 350 g for 5 min, and the resulting cell pellet was resuspended in cold 0.02% BSA. Single-cell RNA sequencing library preparation was performed using the Chromium Single Cell 3′ Reagent V3 kit, following the manufacturer's specifications. Barcoded libraries were sequenced on the Illumina NovaSeq platform with 150 bp paired-end reads, achieving an average depth of approximately 30,000 reads per cell.

SHARE-seq library preparation and sequencing

SHARE-seq libraries were prepared as previously described (Ma et al., 2020). Briefly, cells were fixed using 0.2% formaldehyde and permeabilized. For joint measurements of single-cell chromatin accessibility and expression (scATAC- and scRNA-seq), cells were first transposed by Tn5 transposase to mark regions of open chromatin. The mRNA was reverse transcribed using a poly(T) primer containing a unique molecular identifier (UMI) and a biotin tag. Permeabilized cells were distributed in a 96-well plate to hybridize well-specific barcoded oligonucleotides to transposed chromatin fragments and poly(T) cDNA. Hybridization was repeated three times to expand the barcoding space and ligate cell barcodes to cDNA and chromatin fragments. Reverse crosslinking was performed to release barcoded molecules. cDNA was separated from chromatin using streptavidin beads, and each library was prepared separately for sequencing. Libraries were sequenced on the Illumina NovaSeq platform (read 1: 50 cycles, read 2: 50 cycles, index 1: 99 cycles, index 2: 8 cycles).

QUANTIFICATION AND STATISTICAL ANALYSIS

10× single-cell RNA-sequencing data processing and analysis

All single-cell RNA-sequencing (scRNA-seq) samples were processed individually. Sequencing reads were aligned to a combined human and mouse reference transcriptome (GRCh38_and_mm10-2020-A) using Cell Ranger (v7.2.0) with default parameters to generate gene-by-cell count matrices. Downstream analyses were performed in R using Seurat (v4.0.1). The data were filtered to retain only genes annotated to the human genome. Genes detected in at least three cells were retained, and cells were retained if they had 200-7,000 detected features and less than 10% of reads mapping to mitochondrial genes. For 7D-patterned neurons and ADNP neuron samples, doublets were removed using DoubletFinder (v2.0.4)

with default parameters. Although the initial Cell Ranger metrics for the WTC11 homozygous ADNP sample were acceptable, only 3,212 cells remained after applying the uniform quality-control criteria, corresponding to a retention rate of 27.6%, compared with 68.0%–83.5% for the other datasets. The retained cells also formed a distinct sample-specific cluster, raising concern that their transcriptional profile reflected technical variation rather than biological genotype effects. This sample was therefore excluded from downstream comparative analyses. The corresponding WTC11 heterozygous sample was retained. Per-sample cell counts and quality-control metrics, including those for the excluded sample, are reported in Table S1.

After preprocessing, samples were merged and normalized using natural-log transformation, and counts were scaled by a factor of 10,000. Principal component analysis (PCA) was conducted on the 2,000 most variable features (FindVariableFeatures()). The first 20 principal components were used to construct the K-nearest-neighbor graph; the first 30 principal components were used for the ADNP analyses. Cells were clustered using the Louvain algorithm (FindClusters()) and visualized using UMAP embeddings. Clusters were manually annotated with cell types using a curated marker list (Table S2). When necessary, samples were integrated using the FastMNN algorithm (IntegrateLayers()). Cell-cycle- and ribosomal-associated genes were excluded from variable-feature selection and therefore did not contribute to PCA, neighbor-graph construction, clustering, or UMAP generation. Differential expression analysis between clusters or samples was performed using the Wilcoxon rank-sum test using only genes expressed by a minimum of 3–5% of cells. Method/cluster specificity genes were identified as previously described (Mullins et al., 2021). Briefly, genes with non-unique names or not expressed in any cells were filtered out. Gene expression was scaled to 1 million unique molecular identifiers per method or cluster. Gene expression specificity was calculated by dividing the expression of each gene in each method/cluster by the total expression of all genes in that method/cluster, resulting in values from 0 to 1, with values closer to 1 indicating high specificity. The top 10% of the most highly specific genes per method or cluster were used for gene expression analyses. Pseudotime trajectory analysis was performed using Slingshot (v2.8.0) or Monocle3 (v.1.3.7) software with default parameters. graph_test() in Monocle3 was used to determine genes that change with pseudotime.

To assess the similarity of PSC-derived GABAergic neurons to inhibitory neurons from different regions of the developing fetal brain, we utilized previously published scRNA-seq data from Bhaduri et al. (2021) encompassing weeks 18, 19, 20, and 25 of gestation across various brain regions (neocortex, striatum, thalamus, hypothalamus, ganglionic eminence, midbrain, and cerebellum). Additionally, hypothalamic neuron data from Herb et al. (2023) were included. All datasets were merged into a single Seurat object for comprehensive analysis. The combined dataset was first refined to focus on neurons expressing the inhibitory neuron markers GAD1/2 (log norm counts >0). We employed Seurat's FindTransferAnchors() and MapQuery() functions for cell mapping and comparison. Data normalization was performed using SCTransform.

To compare cell states across differentiation approaches, publicly available scRNA-seq datasets were independently reprocessed using Seurat. Gene-by-cell count matrices were log-normalized and scaled, and the top 2,000 highly variable genes were selected for dimensionality reduction. Principal component analysis (PCA) was performed using 50 components, followed by UMAP embedding (min.dist = 0.3). To assign lineage and cell-state identities, each dataset was mapped onto a reference atlas of the developing human brain (Feng et al., 2025). The reference dataset was processed using the same pipeline, except that 20 principal components were used for UMAP construction. Anchor-based mapping was performed using FindTransferAnchors() (reduction = "rpca", k.anchor = 20), followed by cell-type annotation with TransferData() (dims = 1:20, k.weight = 10). This strategy enabled consistent assignment of interneuron lineage and regional identities across differentiation methods. To assess reproducibility, the proportions of annotated interneuron populations were quantified for each iPSC line and differentiation method, and pairwise Pearson correlation coefficients of these proportions were computed across the three iPSC lines, across differentiation methods, and against the *in vivo* reference (Figure S5).

SHARE-seq data processing and analysis

SHARE-seq libraries were aligned using a published pipeline (https://github.com/masai1116/SHARE-seq-alignmentV2). Briefly, sequencing reads were trimmed and aligned to the hg38 genome using Bowtie2. Reads were demultiplexed using four sets of 8-bp barcodes in the index reads, allowing one mismatch per barcode. Reads mapping to the mitochondria and chrY were discarded. Duplicates were removed based on the read alignment position and cell barcodes. Open chromatin region peaks were called on merged samples using MACS2. Peaks overlapping with ENCODE blacklisted regions (https://sites.google.com/site/anshulkundaje/projects/blacklists) were filtered out. Peak summits were extended by 250bp on each side and defined as accessible regions. The fragment counts in peaks and TF scores were calculated using chromVAR. SHARE-RNA-seq reads were aligned to the hg38 genome using STAR. Reads were demultiplexed as described above for

SHARE-ATAC-seq. Aligned reads were annotated to both exons and introns using feature counts. PCR duplicates were removed based on UMIs and read alignment position. Cells that expressed >1,000 genes and <4% mitochondrial reads were retained. Seurat was used to preprocess the count matrix and cluster cells for scRNA-seq libraries as described above. For pseudotime trajectory analysis, hPSCs, NPC-like cells, and stressed cells were removed, and Slingshot was utilized with default parameters.

Inferring regulatory networks

To infer transcription factor (TF) regulon networks within our single-cell RNA-seq dataset, we utilized the SCENIC (Single-Cell Regulatory Network Inference and Clustering) (Aibar et al., 2017) protocol (v1.1.2), which employs the vsn-pipeline version orchestrated via Nextflow with the pyscenic (v0.12.1) container. First, Seurat objects were converted into loom files using the SCopeLoomR package (v0.13.0). Subsequently, the pyscenic module was executed 20 times using its default settings. Only regulons identified in at least 15 out of the 20 runs were included for further analysis.

Gene ontology, pathway enrichment and disease-vulnerable cell type analysis

We used Enrichr (Chen et al., 2013) or ClusterProfiler (with universe set to all expressed genes in dataset) to identify the cellular functions and biological processes of differentially expressed genes. To identify the cellular functions of differential peaks, we annotated the peaks to their nearest genes, followed by Enrichr analysis.

To determine the enrichment of genes implicated in autism spectrum disorder (ASD), we used the Simons Foundation Autism Research Initiative (SFARI) database to compile a list of 1,065 ASD-associated genes scored 0–2 or categorized as syndromic. Fisher's exact test was performed to determine the statistical significance of the top 10% of differentially expressed genes specific to PSC-iGABAs or patterned-iGABAs.

For a more comprehensive analysis of all neural-related diseases, we used the ToppGene Suite and selected the DisGeNET Curated database (Pinero et al., 2020) which includes a total of 11,181 annotations. DisGeNET integrates information on gene-disease associations from repositories, GWAS catalogs, animal models, and scientific literature. To control for multiple testing errors, a threshold of 0.05 was applied. We used a random sampling size of 5,000, considering only coding genes to ensure the results were not biased by the size of the gene list, and required a minimum feature count of at least two genes in our DEG list. Only disease associations with an FDR-adjusted p-value ≤ 0.05 (Benjamini-Hochberg for multiple hypothesis testing) were considered significant.

To assess disease vulnerability across neurons generated using our differentiation approaches, we integrated our scRNA-seq data with publicly available reference datasets from postmortem human brain tissue. Specifically, we used datasets from individuals with AD (Gabitto et al., 2024) and ASD (Velmeshev et al., 2019), which define interneuron populations reported to exhibit selective vulnerability in each disease context. Using a reference-mapping framework, we projected cells from these datasets onto our induced GABAergic neuron atlas. We then quantified the fraction of cells generated by each differentiation strategy that mapped to disease-vulnerable cell types, enabling comparative assessment of how well each approach captures interneuron populations implicated in AD- and ASD-related pathology.

WGCNA analysis

A total of 8,516 genes with an average expression level above 0.15 were selected for WGCNA analysis (Langfelder and Horvath, 2008; Zhang and Horvath, 2005). To construct the network, a soft thresholding is selected through scale independence: power = 4 for clusters 0 and 2 and power = 3 for cluster 1. Gene clustering and module assignments were visualized using a dendrogram, highlighting the hierarchical relationships between gene modules.

Statistical analyses

For each experiment, sample sizes, including the number of cells, independent differentiations, batches, and iPSC lines, are specified in the corresponding figure legends and STAR Methods. Statistical analysis was matched to the data structure as noted above in the method details section for single-cell RNA-seq and SHARE-seq experiments. Statistical analyses were performed in RStudio (RStudio Team, 2020) or GraphPad Prism 9. See figure legends for details on specific statistical tests run for each experiment. Statistical significance is represented by a star (*) and indicates a computed p-value <0.05. Graphs and plots were generated using Graphpad Prism or RStudio. Figures were generated using Adobe Illustrator and BioRender.

Stem Cell Reports, Volume *21*

Supplemental Information

Directing GABAergic neuronal identity from human iPSCs through developmental patterning

Ruiqi Hu, Linda L. Boshans, Yiran Tao, Bohan Zhu, Junlong Li, Xindi Li, Rui Dang, Marianna Liang, Peiwen Cai, Mark Youssef, Gizem Inak, Yingnan Song, Xuran Wang, Alexander M. Tsankov, Joseph D. Buxbaum, Peng Jiang, Zhiping P. Pang, Sai Ma, and Nan Yang

Figure S1. Generation of immature cortical GABAergic neurons from human PSCs using a directed differentiation protocol.
Related to Figure 1.

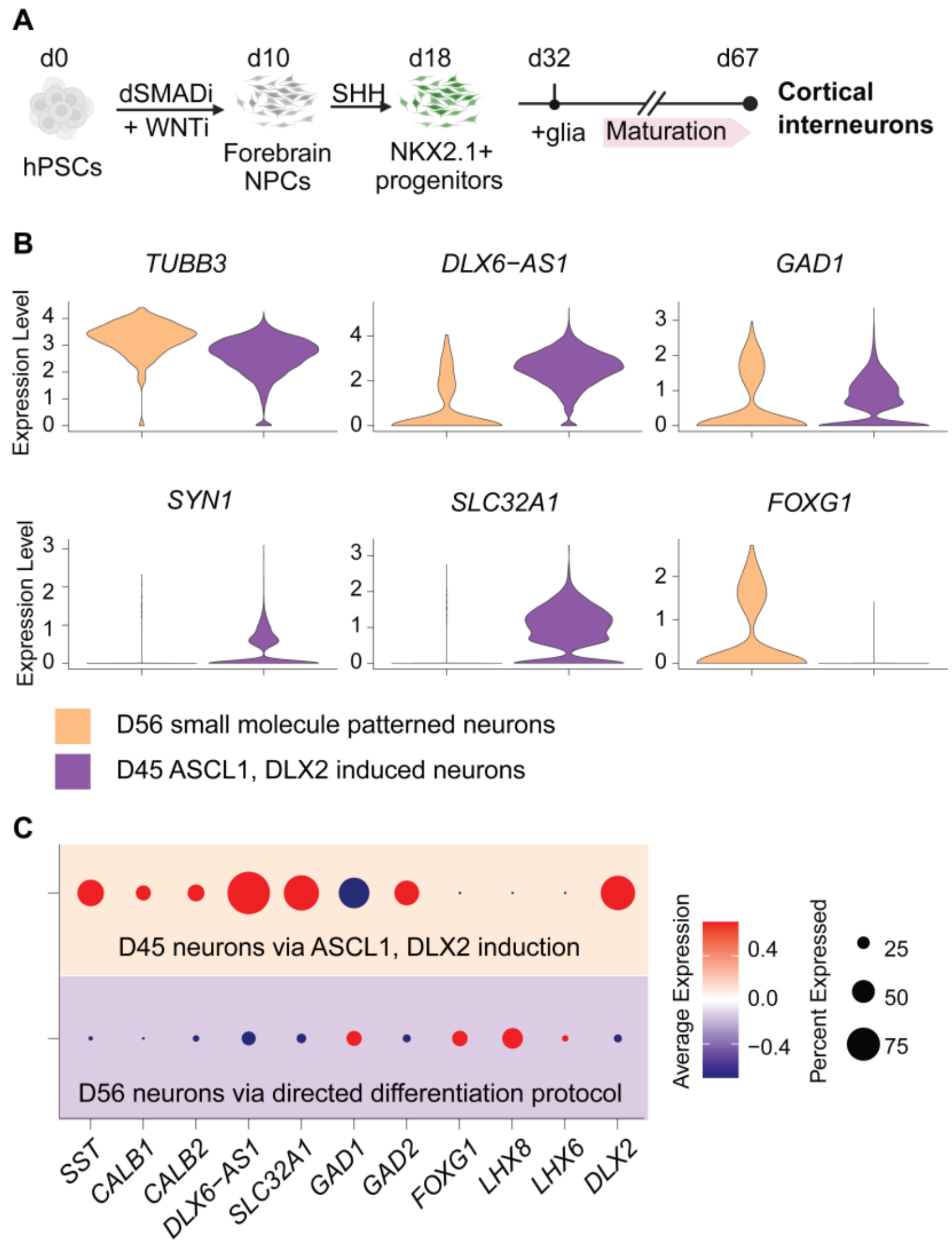

Figure S1. (**A**) Schematic overview and timeline of the small molecule method, followed by single-cell transcriptomics analysis. (**B-C**) Violin plots (**B**) and dot plots (**C**) show expression levels of neuronal identity and maturation-related marker genes in GABAergic neurons generated using either transcription factors (TFs), i.e., ASCL1 and DLX2, or small molecules.

Figure S2. Induction of neuronal fate using ASCL1/DLX2 and patterning factors.
Related to Figure 1.

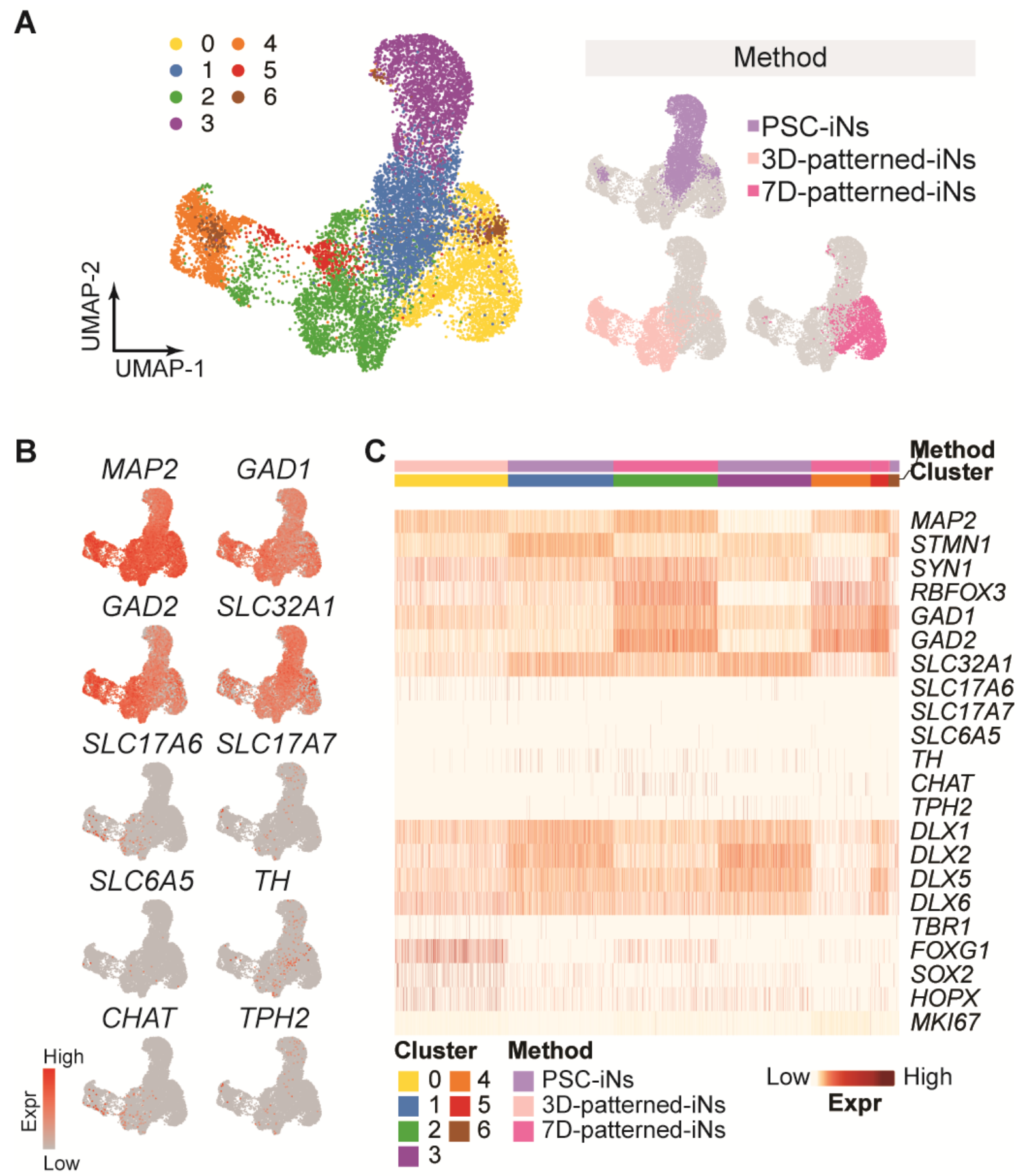

Figure S2. (**A**) UMAP embedding of 12,876 cells, color-coded by cluster identity (left panel) and differentiation method (right panel). (**B**) Feature plots for neuron subtype-specific markers. (**C**) Heatmap of cell type-specific marker gene expression across clusters. See also Table S1.

Figure S3. Expression of neuronal subtype, maturation, and brain regionality marker genes in GABAergic-induced neurons.

Related to Figure 1.

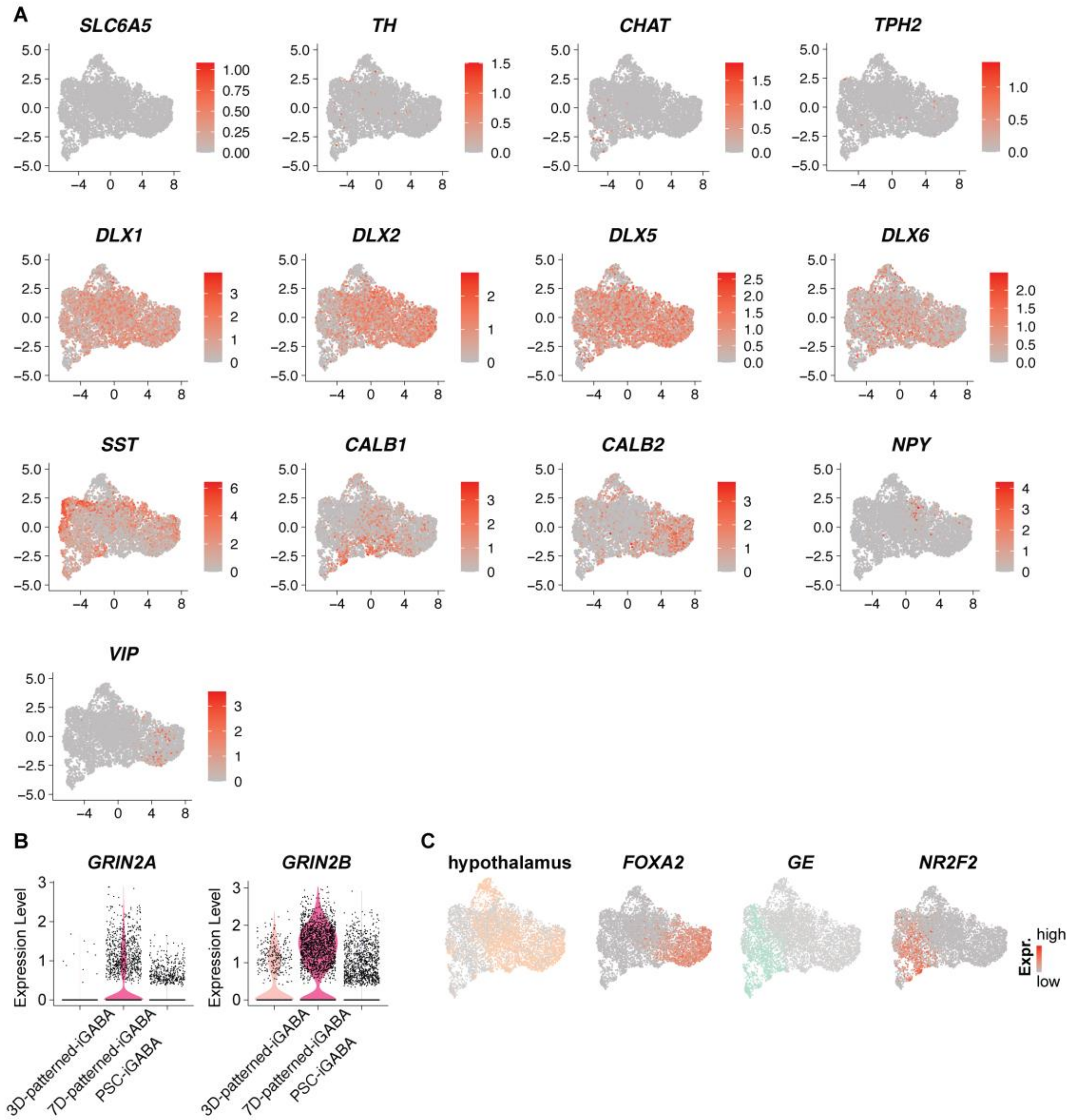

Figure S3. (A) Feature plots showing the expression of additional neuronal subtype marker genes in GABAergic-induced neurons. **(B)** Violin plots displaying the expression levels of NMDA receptor subunits in GABAergic neurons generated using different methods. **(C)** UMAP visualization depicting the respective brain regions and relevant TFs. See also Figure 1.

Figure S4. Comparison of interneuron subtype composition across differentiation methods.
Related to Figure 1.

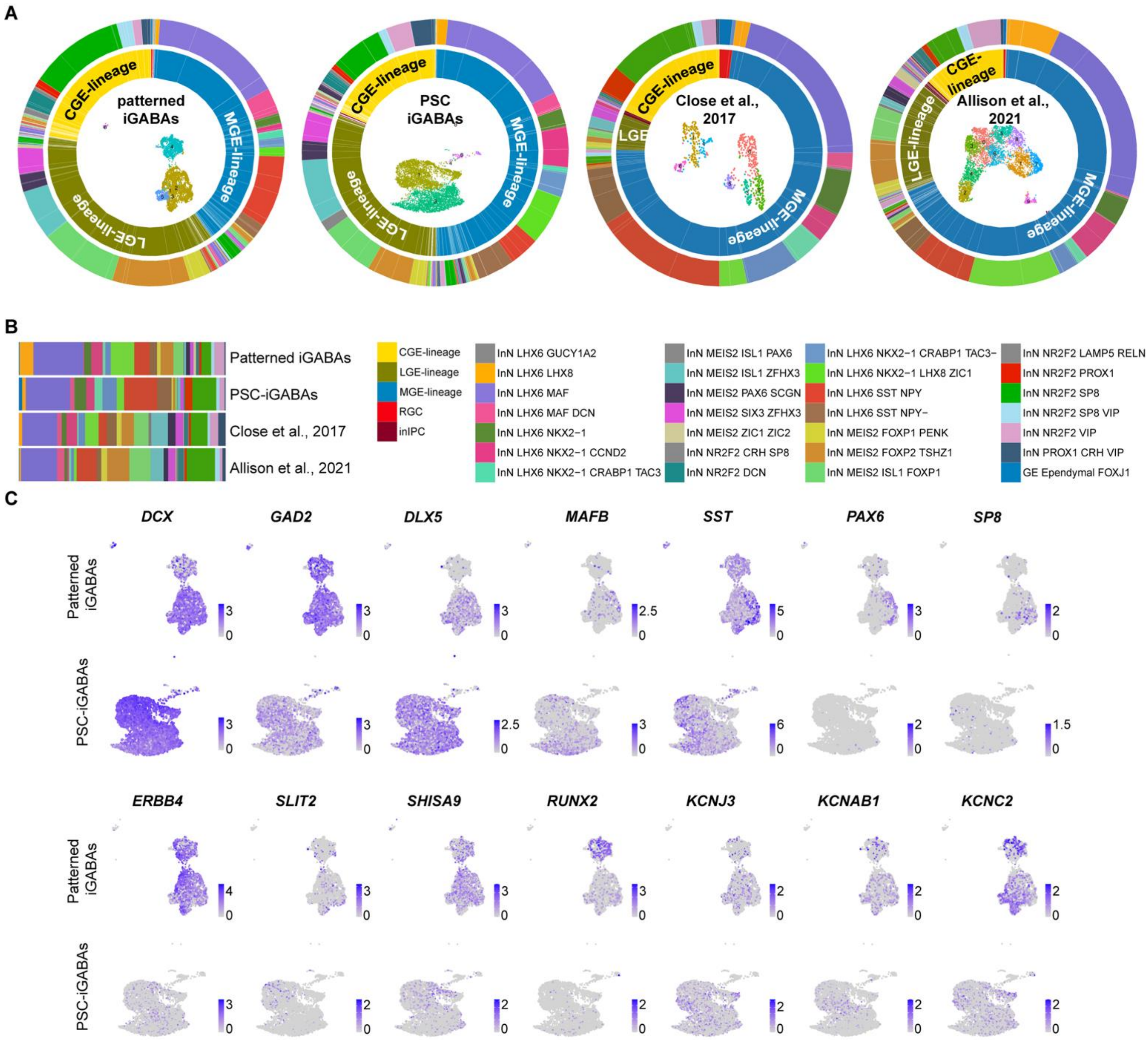

Figure S4. (**A**) Transcriptomic mapping of human PSC-derived interneurons generated using four protocols, patterned-iGABAs and PSC-iGABAs from this study, and patterning-factor–mediated differentiation datasets from Close et al. (2017) and Allison et al. (2021), to reference human brain interneuron datasets. The outer rings indicate the relative abundance of mapped interneuron subtypes. (**B**) Proportional representation of major interneuron lineage groups (MGE, CGE, LGE) across the different methods, highlighting their differing patterning tendencies. (**C**) Feature plots showing expression of representative GABAergic and interneuron-subtype marker genes (top: *DCX, GAD2, DLX5, MAFB, SST, PAX6, SP8*) and fast-spiking/PV-lineage–associated genes (bottom: *ERBB4, SLIT2, SHISA9,*

RUNX2, and the voltage-gated potassium-channel subunits *KCNJ3, KCNAB1, KCNC2*) in patterned-iGABAs and PSC-iGABAs. See also Figure 1.

Figure S5. Quantitative similarity and cross-line reproducibility of GABAergic subtype composition.
Related to Figures 1 and 2.

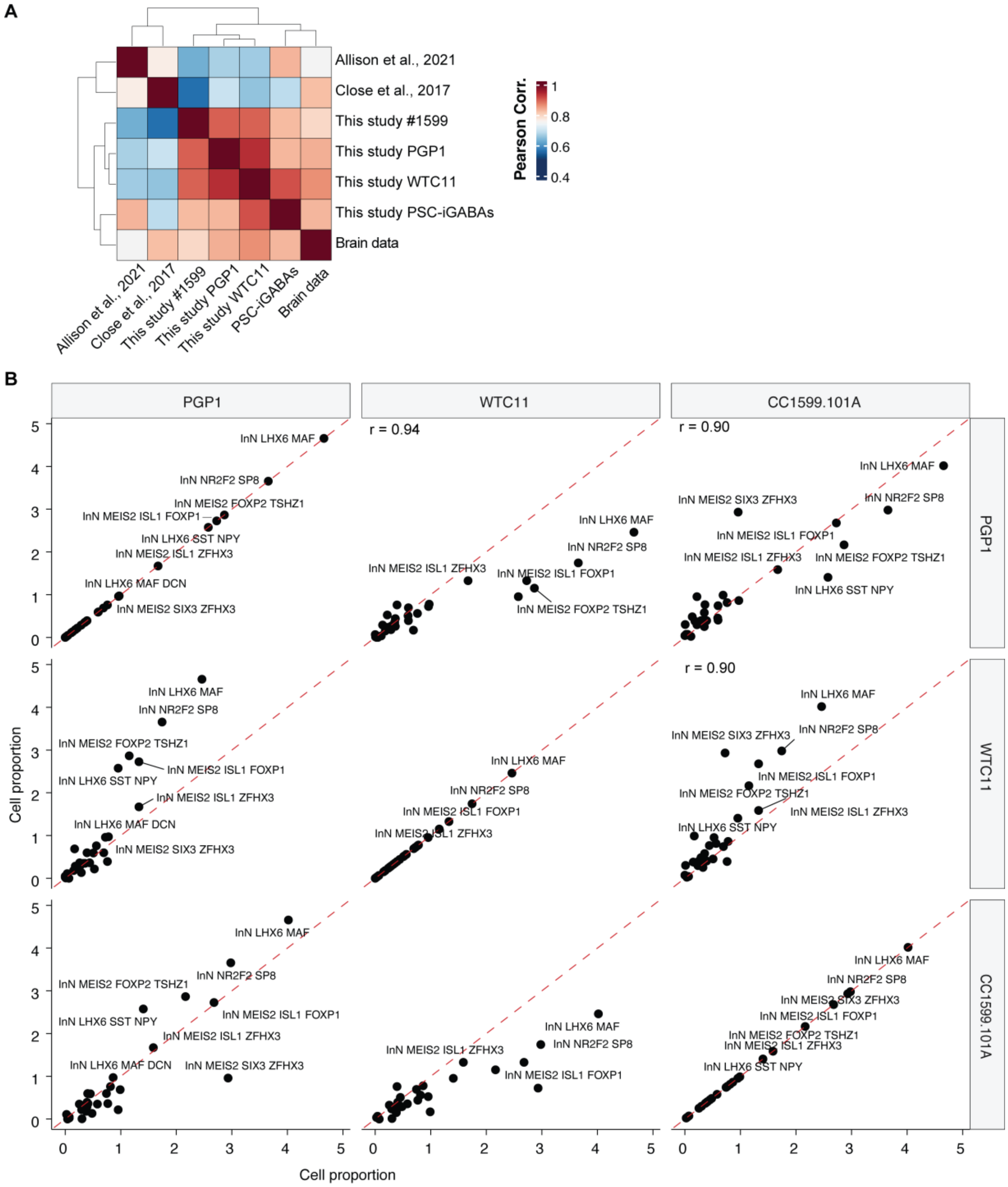

Figure S5. (**A**) Hierarchically clustered heatmap of pairwise Pearson correlations between interneuron subtype proportions, computed across GABAergic neurons generated in this study (patterned-iGABAs and PSC-iGABAs), two published protocols (Close et al., 2017; Allison et al., 2021), three iPSC lines

(PGP1, WTC11, #1599 (CC1599.101A)), and the human brain interneuron reference dataset (Feng et al., 2025). Warmer colors indicate higher correlation (greater similarity); patterned-iGABAs show the closest correspondence to the in vivo brain reference among the in vitro conditions. (**B**) Pairwise comparison of interneuron subtype proportions ("cell proportion") among the three genetically distinct iPSC lines (PGP1, WTC11, CC1599.101A). Each point represents one GABAergic subtype; the dashed line denotes identity (y = x). Pearson correlation coefficients are shown for each line pair (r = 0.90-0.94), indicating highly reproducible subtype composition across genetic backgrounds.

Figure S6. Functional comparison of patterned-iGABA and TF-only PSC-iGABA neurons.
Related to Figure 2.

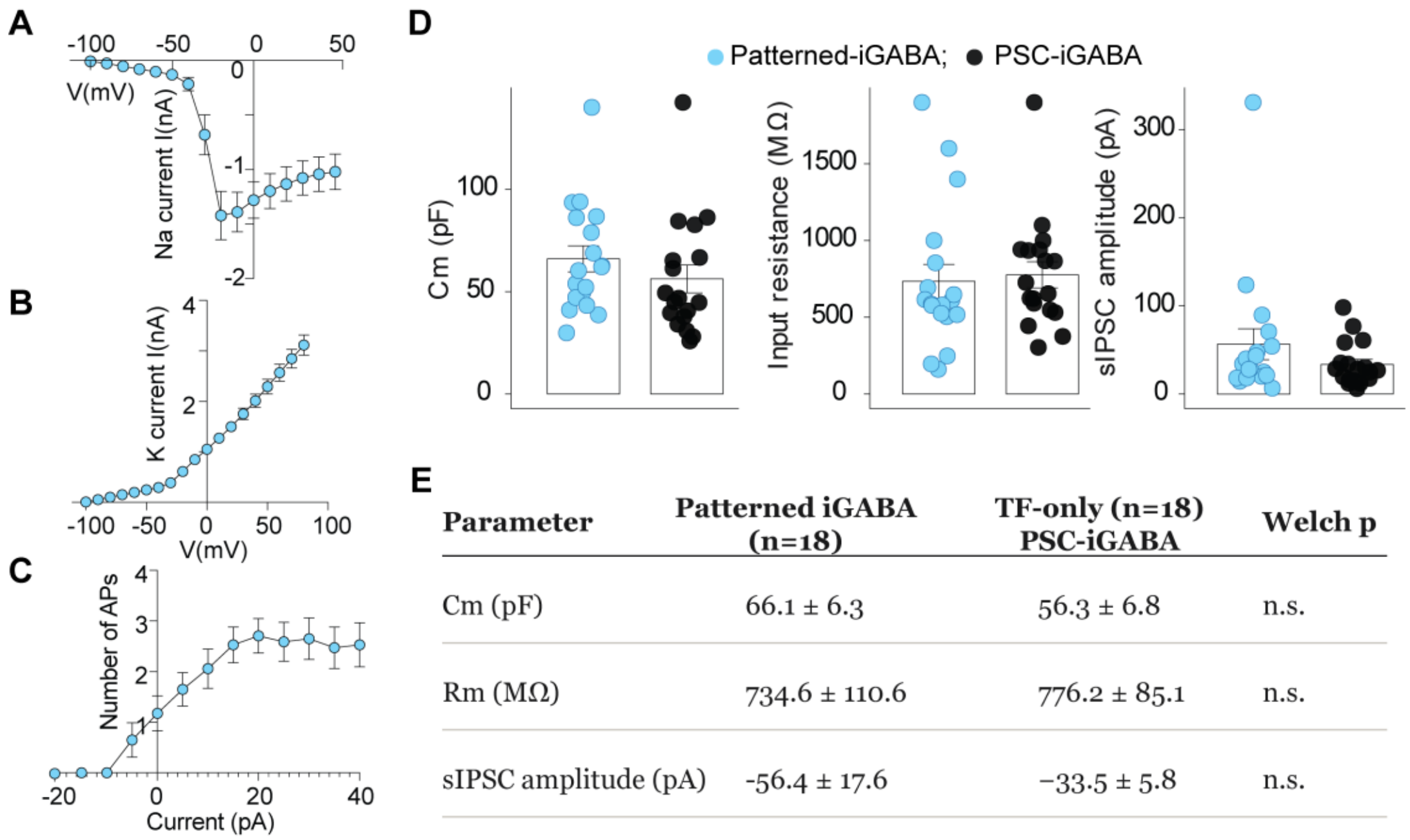

Parameter	Patterned iGABA (n=18)	TF-only (n=18) PSC-iGABA	Welch p
Cm (pF)	66.1 ± 6.3	56.3 ± 6.8	n.s.
Rm (MΩ)	734.6 ± 110.6	776.2 ± 85.1	n.s.
sIPSC amplitude (pA)	-56.4 ± 17.6	−33.5 ± 5.8	n.s.

Figure S6. Whole-cell patch-clamp recordings were performed on patterned-iGABA neurons after ~5 weeks of maturation on astrocytes (day 35). (**A**) Voltage-clamp current-voltage (I-V) relationship of voltage-gated sodium currents; peak inward current is plotted against step command potential (n = 20 cells). Neurons exhibited fast, voltage-dependent inward currents characteristic of functional voltage-gated Na^+ channels. (**B**) Voltage-clamp I-V relationship of voltage-gated potassium currents; outward current is plotted against command potential (n = 20 cells). (**C**) Current-clamp input–output relationship: number of action potentials evoked by depolarizing current injections of increasing amplitude (n = 20 cells). Patterned-iGABA neurons fired repetitive, current-graded action potentials, confirming intrinsic excitability. (**D**) Comparison of intrinsic and synaptic properties between patterned-iGABA neurons (blue) and TF-only PSC-iGABA neurons (black); the latter generated with ASCL1/DLX2 alone and recorded by the same experimenter under matched conditions. Left, membrane capacitance (Cm); middle, input resistance (Rm); right, spontaneous IPSC (sIPSC) amplitude (shown as magnitude). Bars, mean ± SEM; circles, individual neurons. (**E**) Summary of the parameters in (**D**): mean ± SEM, number of cells, and P values (Welch's unpaired two-tailed t-test). No significant differences were detected between the two protocols for any measured parameter (n.s., P > 0.05), indicating similar basic membrane and inhibitory synaptic properties under the conditions tested.

Figure S7. In vivo survival and neuronal differentiation of transplanted patterned-iNs.
Related to Figure 3.

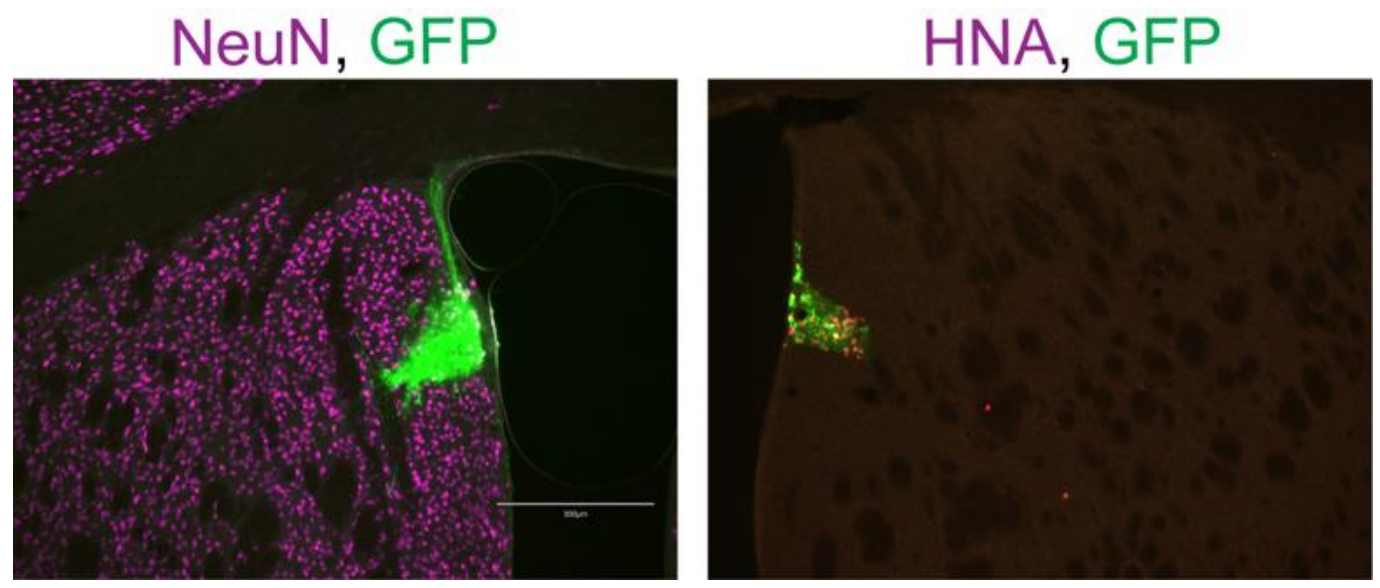

Figure S7. At 5 months post-transplantation, immunohistochemical analysis revealed that the majority of transplanted human cells, identified by human nuclear antigen (HNA) and GFP expression, differentiated into NeuN$^+$ neurons. Most engrafted cells were detected in subcortical areas adjacent to the injection site, including the lateral septum and striatum. Scale bar: 300μm. These findings support long-term survival and neuronal differentiation of patterned-iNs after neonatal xenotransplantation. See also Figure 3.

Figure S8. Disease gene enrichment in GABAergic PSC-iGABAs and patterned-iGABAs.
Related to Figure 1.

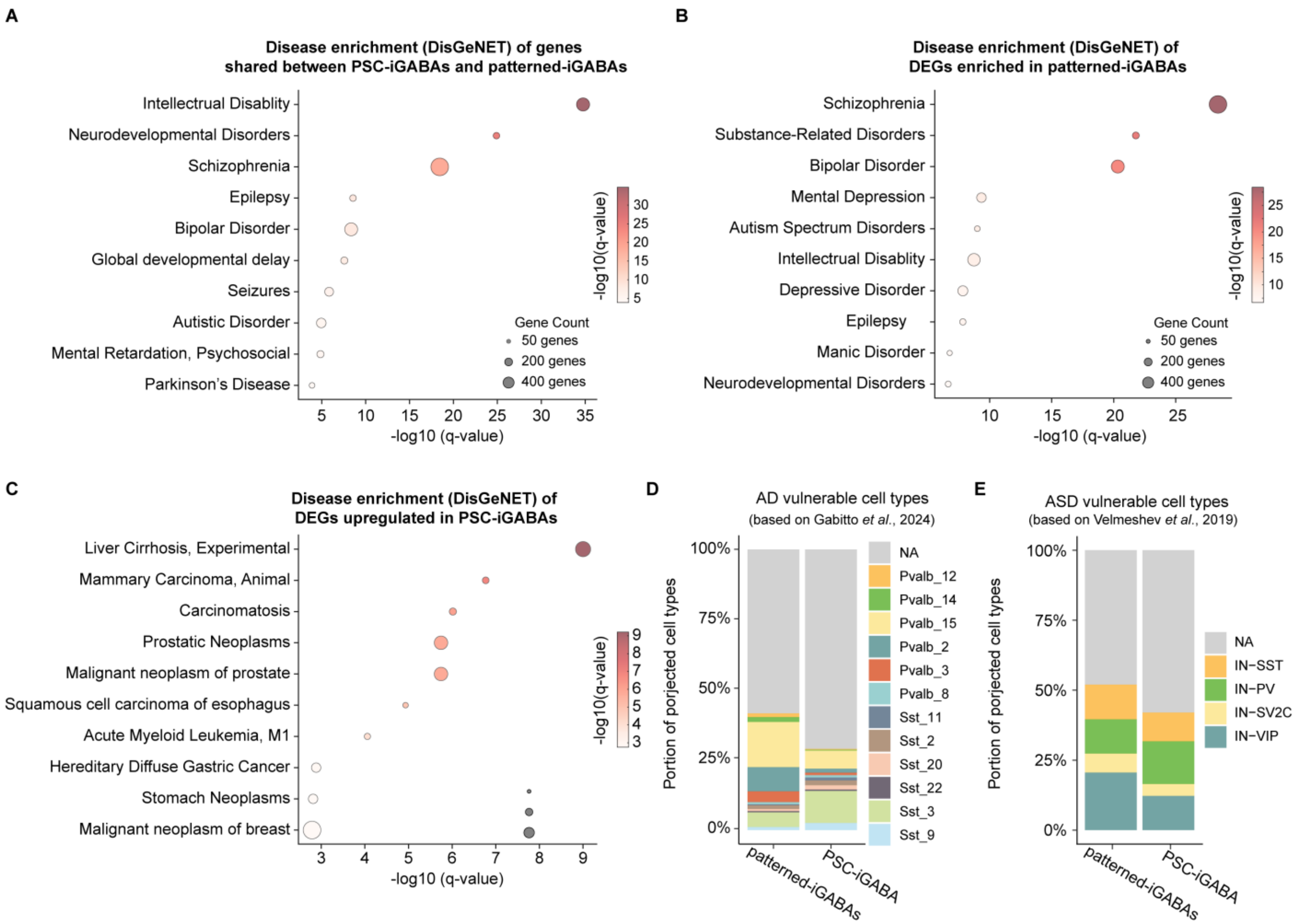

Figure S8. (A–C) Disease enrichment analysis of differentially expressed genes (DEGs) using the DisGeNET platform (Pinero et al., 2020). **(A)** Diseases associated with DEGs shared between PSC-induced GABAergic neurons (PSC-iGABAs) and patterned induced GABAergic neurons (patterned-iGABAs). **(B)** Diseases enriched in DEGs specific to patterned-iGABAs. **(C)** Diseases enriched in DEGs specific to PSC-iGABAs. The x-axis represents the gene count for each disease, and dot size indicates the number of genes associated, while color intensity reflects –log10(p-value) enrichment significance. **(D, E)** Assessment of disease-vulnerable neuronal subtypes represented in the two differentiation methods. **(D)** Cell-type overlap analysis of GABAergic neuron subtypes generated by patterned-iNs and PSC-iNs with AD-vulnerable cell types (based on Gabitto et al., 2024). **(E)** Similar analysis comparing subtype overlap ASD-vulnerable interneuron types (based on Velmeshev et al., 2019). These results demonstrate that both differentiation strategies can generate interneuron subtypes implicated in neurodevelopmental and neurodegenerative disorders, providing a foundation for disease modeling applications.

Figure S9. Gene regulatory network (GRN) analysis centered on method-specific modulators. *Related to Figure 4.*

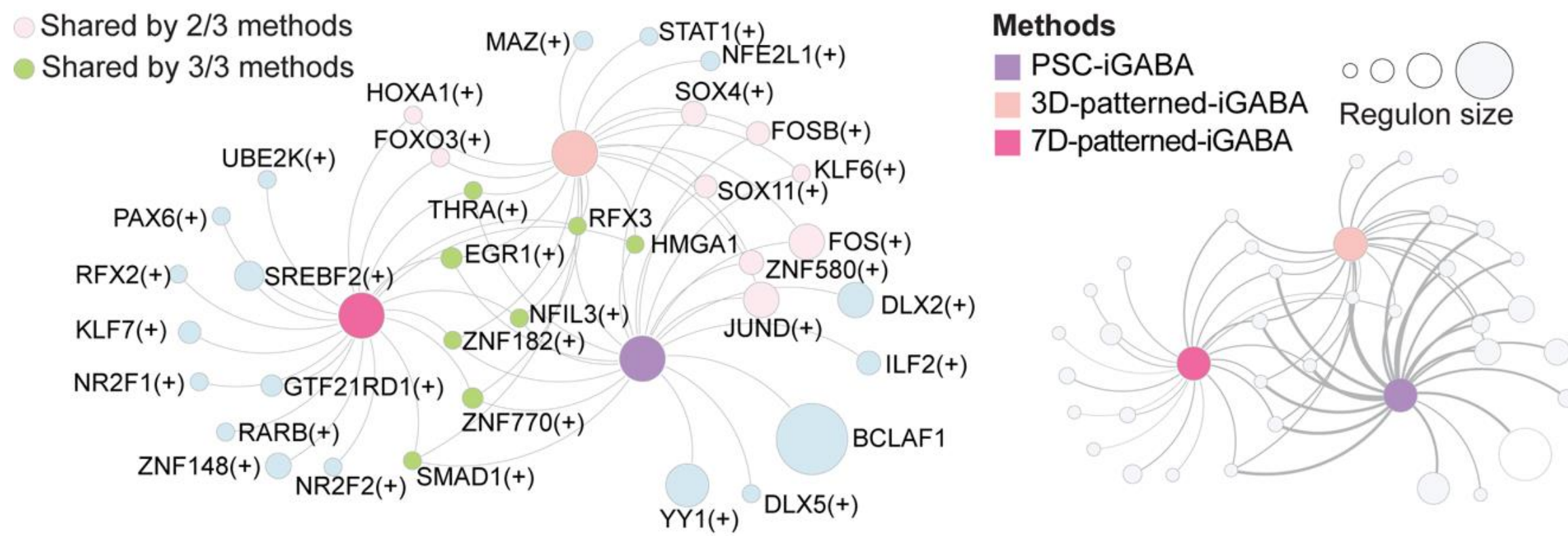

Figure S9. Left: Regulons are color-coded based on uniqueness, with node size proportional to regulon size, indicating the number of target genes regulated by each TF. Right: The same GRN with edge thickness represents the strength of the association between method modulators and regulons. See also Figure 4.

Figure S10. Differential expression of neural progenitor cell genes and their induction along the differentiation trajectory of PSC-iNs and patterned-iNs.
Related to Figure 5.

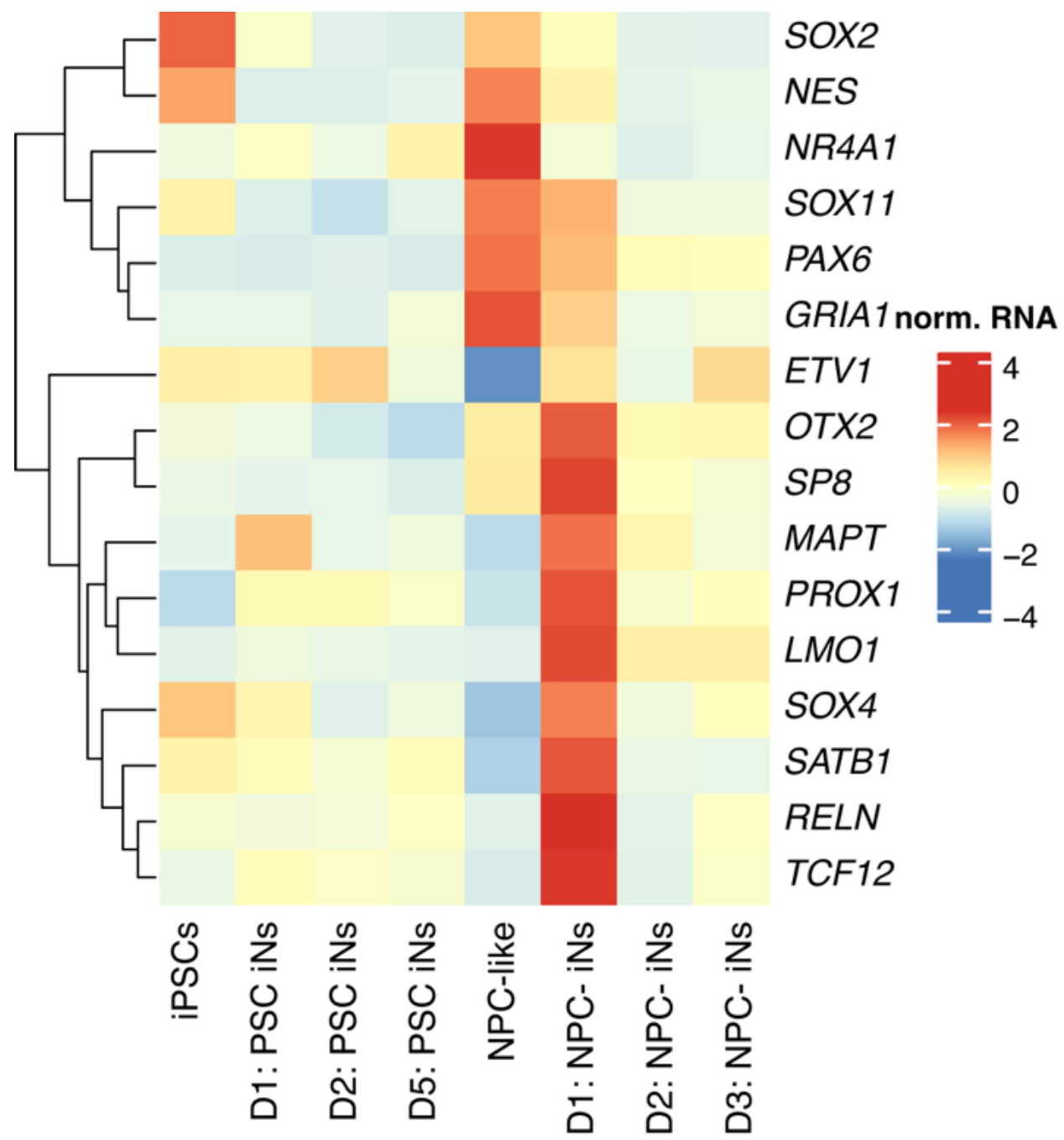

Figure S10. Heatmap showing gene expression across pseudobulk samples, aggregated from individual differentiation stages along the PSC-iNs and patterned iNs trajectory. See also Figure 5.

Figure S11. Non-interneuron lineage genes are minimally induced in GABAergic PSC-iNs and patterned-iNs.
Related to Figure 5.

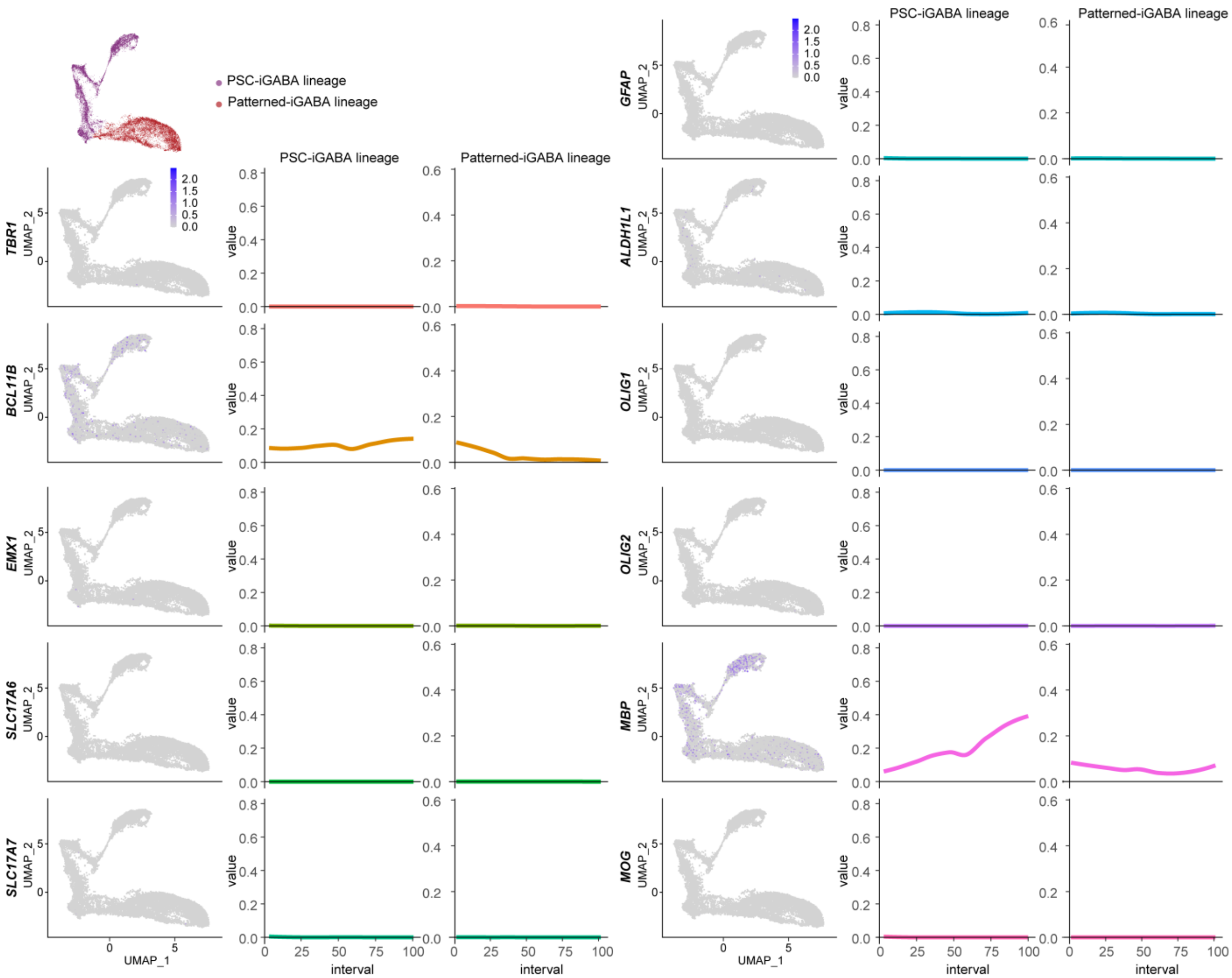

Figure S11. UMAP and pseudotime trajectory plots showing expression dynamics of non-GABAergic lineage markers in PSC-derived GABAergic neurons (PSC-iGABAs) and patterned induced GABAergic neurons (patterned-iGABAs). Expression of genes associated with alternative lineages, including *GFAP*, *ALDH1L1* (astrocyte), *TBR1*, *BCL11B*, *EMX1*, *SLC17A6*, *SLC17A7* (excitatory neurons), and *OLIG1*, *OLIG2*, *MBP*, *MOG* (oligodendrocyte), was analyzed across the differentiation trajectory. Expression of these non-interneuron markers remained low or undetectable across both lineages, indicating minimal contamination by alternative cell fates. Notably, MBP showed mild induction in the PSC-iGABA lineage but not in patterned-iGABAs, suggesting a slightly greater propensity for glial gene activation in PSC-derived cells. These data support the lineage specificity of both differentiation protocols, with rare off-target induction of non-interneuron identities. See also Figure 5.

Figure S12. Generation of iPSC lines with HA-tagged wild-type ADNP or ADNP$^{p.Tyr719*}$
Related to Figure 6.

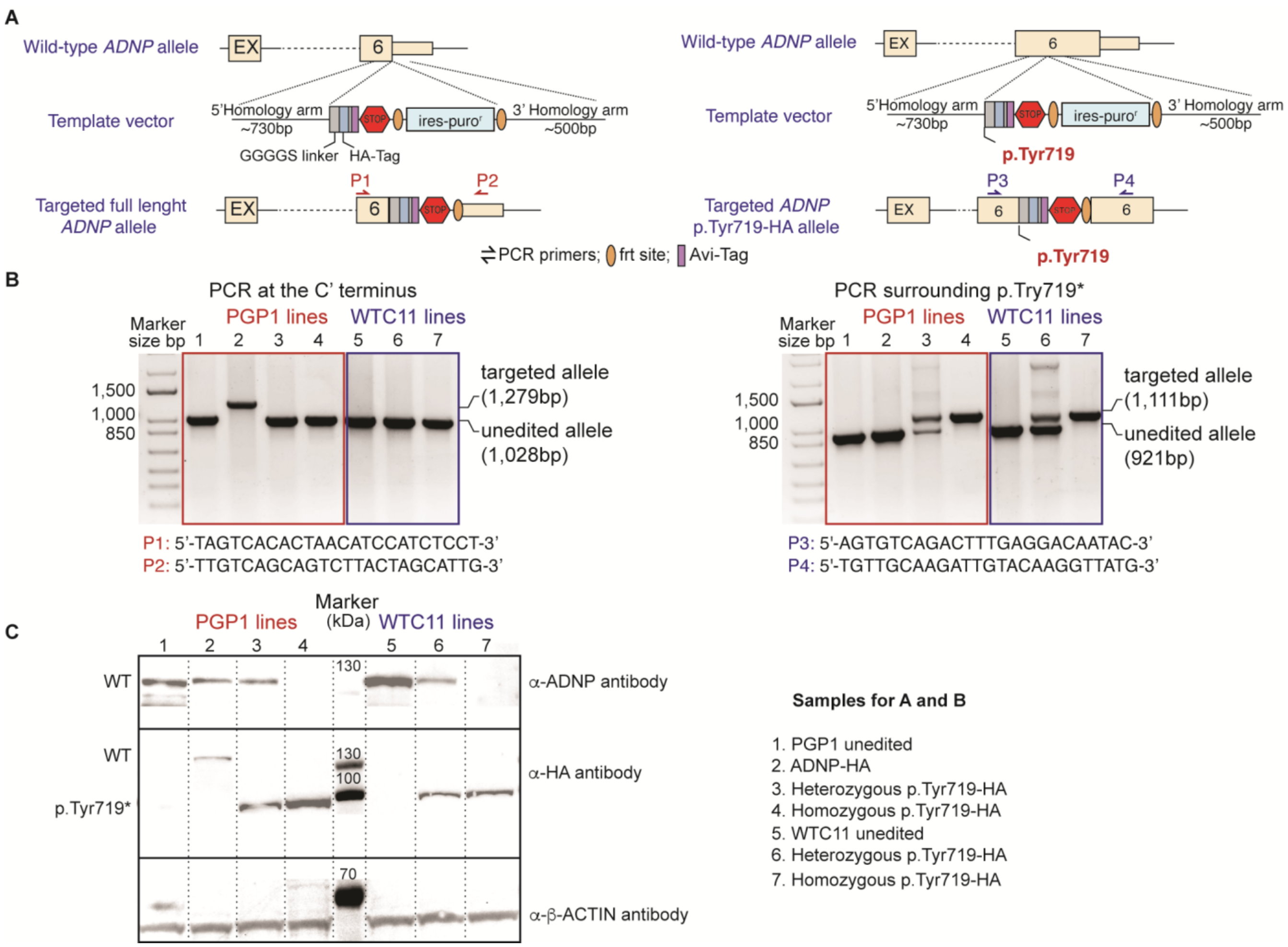

Figure S12. (**A**) Schematic representation of the generation of HA-tagged wild-type ADNP (left) and ADNP$^{p.Tyr719*}$ (right) iPSC lines. The targeting vectors included two homology arms flanking a selection cassette containing an internal ribosomal entry site (IRES), a puromycin resistance gene (puro), and a polyadenylation signal. The selection cassette was flanked by two flippase recognition target (FRT) sites, and FlipE recombinase was expressed to remove the selection cassette. (**B**) Validation of homologous recombination by PCR. The HA-tagged wild-type allele (left) produced a band of approximately 1.2 kb, while the wild-type allele produced a band of approximately 1 kb. The HA-tagged ADNPp.Tyr719* allele produced a band of approximately 1.1 kb, and the unedited allele produced a band of approximately 900 bp. Primer sequences are provided. (**C**) Immunoblotting analysis confirmed the expression of full-length ADNP and ADNP$^{p.Tyr719*.}$ β-ACTIN was used as the loading control. See also Figure 6.

Figure S13. ***ADNP$^{p.Tyr719*}$*** **mutation disrupts neurogenesis.**
Related to Figure 6.

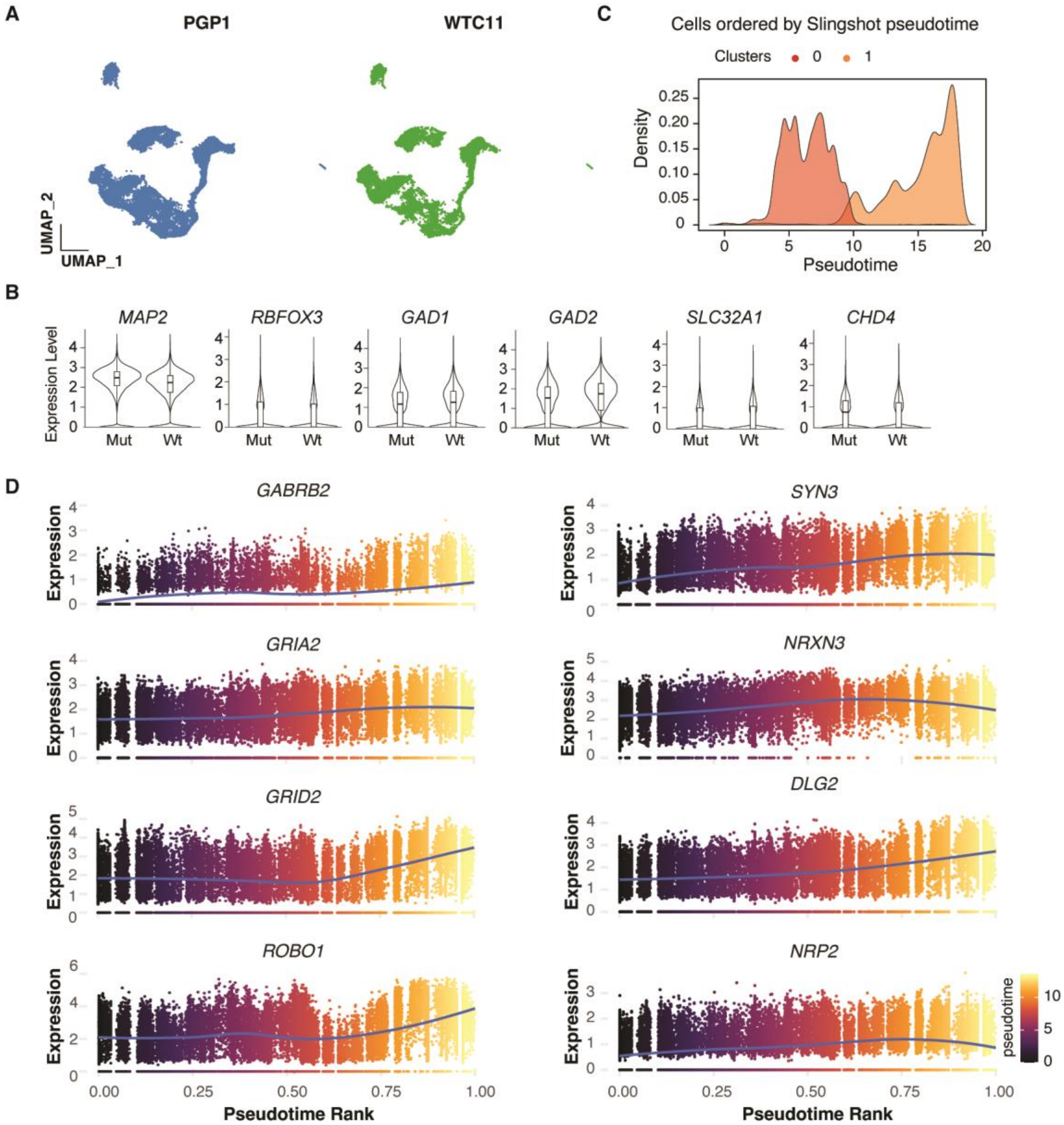

Figure S13. (**A**) Combined UMAP visualization of cells derived from different donor iPSCs. (**B**) Violin plots displaying the expression levels of pan-neuron and GABAergic neuron marker genes. (**C**) Inferred pseudotime analysis showing the progression from cluster 0 to cluster 1. The panel shows the density of each cluster across pseudotime. (**D**) Pseudotime expression plots show an increasing trend in genes related to neurite growth and synapse formation as cells progress along the pseudotime trajectory. See also Figure 6.

Figure S14. WGCNA reveals disruption of gene expression networks involved in neuronal development and function in mutant cells.
Related to Figure 6.

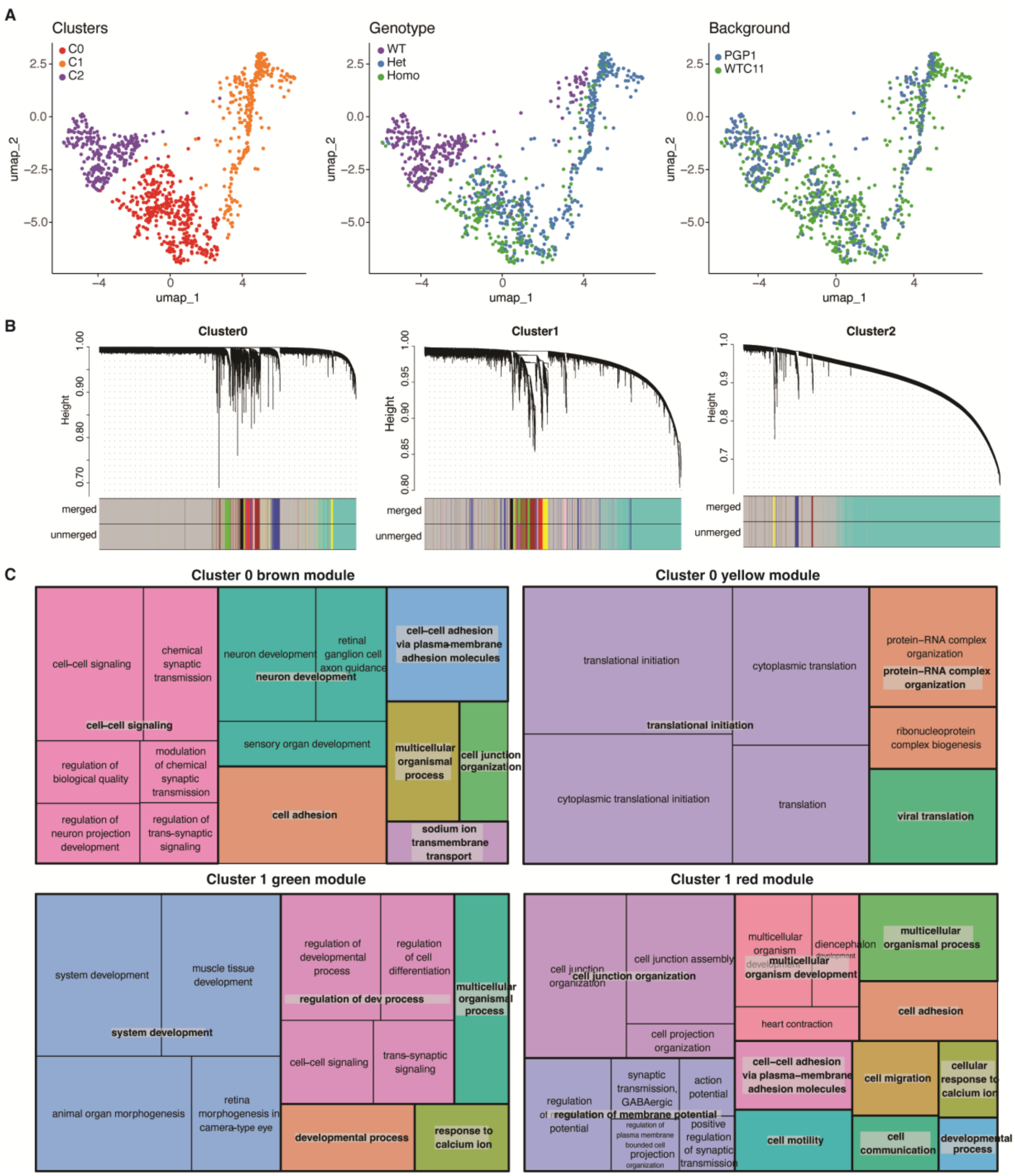

Figure S14. **(A)** Metacells maintain the cluster structure and accurately represent the genotypes and iPSC donor identities. **(B)** Gene expression modules identified within clusters 0, 1, and 2. **(C)** Treeplots displaying GO terms for biological processes associated with different modules in clusters consisting of mutant cells. See also Figure 6.

REFERENCES

Allison, T., Langerman, J., Sabri, S., Otero-Garcia, M., Lund, A., Huang, J., Wei, X., Samarasinghe, R.A., Polioudakis, D., Mody, I., et al. (2021). Defining the nature of human pluripotent stem cell-derived interneurons via single-cell analysis. Stem Cell Rep 16, 2548-2564. https://doi.org/10.1016/j.stemcr.2021.08.006.

Close, J.L., Yao, Z., Levi, B.P., Miller, J.A., Bakken, T.E., Menon, V., Ting, J.T., Wall, A., Krostag, A.R., Thomsen, E.R., et al. (2017). Single-Cell Profiling of an In Vitro Model of Human Interneuron Development Reveals Temporal Dynamics of Cell Type Production and Maturation. Neuron 93, 1035-1048.e5. https://doi.org/10.1016/j.neuron.2017.02.014.

Feng, X., Gao, Y., Chu, F., Shan, Y., Liu, M., Wang, Y., Zhu, Y., Lu, Q., and Li, M. (2025). Cortical arealization of interneurons defines shared and distinct molecular programs in developing human and macaque brains. Nat Commun 16, 672. https://doi.org/10.1038/s41467-025-56058-8.

Gabitto, M.I., Travaglini, K.J., Rachleff, V.M., Kaplan, E.S., Long, B., Ariza, J., Ding, Y., Mahoney, J.T., Dee, N., Goldy, J., et al. (2024). Integrated multimodal cell atlas of Alzheimer's disease. Nat Neurosci 27, 2366-2383. https://doi.org/10.1038/s41593-024-01774-5.

Pinero, J., Ramirez-Anguita, J.M., Sauch-Pitarch, J., Ronzano, F., Centeno, E., Sanz, F., and Furlong, L.I. (2020). The DisGeNET knowledge platform for disease genomics: 2019 update. Nucleic Acids Res 48, D845-D855. https://doi.org/10.1093/nar/gkz1021.

Velmeshev, D., Schirmer, L., Jung, D., Haeussler, M., Perez, Y., Mayer, S., Bhaduri, A., Goyal, N., Rowitch, D.H., and Kriegstein, A.R. (2019). Single-cell genomics identifies cell type-specific molecular changes in autism. Science 364, 685-689. https://doi.org/10.1126/science.aav8130.
